# Genomic decoding of drug-resistant tuberculosis transmission in Thailand over three decades

**DOI:** 10.1038/s41598-025-15093-7

**Published:** 2025-08-13

**Authors:** Naphatcha Thawong, Prapaporn Srilohasin, Jody E. Phelan, Worawich Phornsiricharoenphant, Sissades Tongsima, Prapat Suriyaphol, Therdsak Prammananan, Kiatichai Faksri, Waritta Sawaengdee, Linfeng Wang, Woranich Hinthong, Martin L. Hibberd, Susana Campino, Sukanya Wattanapokayakit, Surakameth Mahasirimongkol, Angkana Chaiprasert, Taane G. Clark

**Affiliations:** 1 Department of Infection Biology, Faculty of Infectious and Tropical Diseases, London School of Hygiene and Tropical Medicine, London, WC1E 7HT UK; 2 Department of Medical Sciences, Ministry of Public Health, Nonthaburi, Thailand; 3 Faculty of Medicine, Siriraj Hospital, Mahidol University, Bangkok, 10700 Thailand; 4 Ministry of Higher Education, Science, Research and Innovation, National Science and Technology Development Agency, Bangkok, Thailand; 5 Faculty of Medicine, Khon Kaen University, Khon Kaen, Thailand; 6 Faculty of Epidemiology and Population Health, London School of Hygiene & Tropical Medicine, London, UK

**Keywords:** *Mycobacterium tuberculosis*, Tuberculosis, Transmission, Drug resistance, Genomics, Thailand, Genetics, Microbial genetics, Bacterial genetics, Diseases, Infectious diseases, Tuberculosis

## Abstract

Thailand has a high burden of tuberculosis, with control efforts hindered by drug-resistant *Mycobacterium tuberculosis* (Mtb). The increasing use of whole-genome sequencing (WGS) of Mtb offers valuable insights for clinical management and public health surveillance. WGS can be used to profile drug resistance, identify circulating sub-lineages, and trace transmission pathways or outbreaks. We analysed WGS data from 2,005 Mtb isolates collected across Thailand from 1994–2020, including 816 retrieved and 1,189 newly sequenced samples, with most isolates being multidrug-resistant (MDR-TB). Most isolates are lineage two strains (78·3%), primarily the Beijing sub-lineage (L2.2.1). Drug resistance profiling revealed substantial isoniazid and rifampicin resistance, and 67·3% classified as MDR-TB. Phenotypic and genotypic drug susceptibility testing showed high concordance (91·1%). Clustering analysis identified 206 transmission clades (maximum size 288), predominantly with MDR-TB, especially in Central and Northeastern regions. One cluster (n = 22) contains the *ddn* Gly81Ser mutation, linked to delamanid resistance, with some members pre-dating drug roll-out. In the largest cluster (n = 288), containing isolates spanning two decades, we applied transmission reconstruction methods to estimate a mutation rate of 1·1 × 10^–7^ substitutions per site per year. Overall, this study demonstrates the value of WGS in uncovering TB transmission and drug resistance, offering key data to inform better control strategies in Thailand and elsewhere.

## Introduction

Tuberculosis (TB), caused by the bacterium *Mycobacterium tuberculosis* (Mtb), remains a persistent and formidable global health challenge, causing 10·8 M cases and 1·1 M deaths in 2023 alone. Despite significant advancements in medical science and public health initiatives^1^, the WHO South-East Asia region (WHO SEA), which is home to around one-fourth of the world’s population, has more than 45% burden of annual TB incidence. Thailand had amongst the highest rates of TB incidence (157/100 K population, ~ 113 K cases) in 2023, with an increase by 4·4% from 2022, and combined with prevalent drug resistance, will make World Health Organization (WHO) “End TB” targets difficult to achieve. Resistance levels to frontline rifampicin (RR-TB) and isoniazid (HR-TB) drugs, together called multi-drug resistance (MDR-TB), are high across WHO SEA (172 K cases; 8·4/100 K population). Worryingly, extensively drug-resistant TB strains (XDR; MDR + fluoroquinolones + group A resistance) and pre-XDR (MDR + fluoroquinolones) forms exist, providing a progression of resistance and limiting treatment options. In response, recent WHO guidelines suggest a 6-month regimen comprising bedaquiline, pretomanid, linezolid and moxifloxacin (BPaLM) to reduce both treatment duration and non-compliance levels due to drug toxicity. However, bedaquiline resistance is increasing, and with anti-TB drugs being costly with long durations (> 6-months), personalised treatment based on Mtb resistance knowledge is crucial^2^.

Successful worldwide efforts to decrease TB burden have focused on advanced algorithms for early diagnosis, appropriate therapy choice and active case finding. However, these approaches have not been applied systematically across WHO SEA^3^. Whilst diagnostics endorsed for TB and drug resistance detection (e.g., Xpert MTB/RIF, XDR) as part of the WHO’s “End TB” strategy are rapid compared to laboratory phenotypic drug susceptibility tests (pDSTs), they are costly and do not capture all genetic mutations required for precise management of advanced drug resistance forms. For example, the new Xpert XDR cartridge will not detect bedaquiline, linezolid, clofazimine and delamanid resistance, and does not cover some useful first-line drugs (e.g., rifabutin, ethambutol). Recent successes in TB treatment decision-making in developed countries have been led by advances in next generation sequencing technologies (NGS; e.g., Illumina, Oxford Nanopore Technologies (ONT))^4^, with increasing opportunities to use these directly from sputum or DNA from limited Mtb culture (MGIT), in near real time, at decreasing costs^5^. Whole genome (WGS) and targeted gene amplicon (AMP-SEQ) sequencing data generated using NGS can be used to profile Mtb for drug resistance and strain-types (sub-lineages), as well as infer transmission events or outbreaks through sequence similarity^5–7^, facilitated through advances in health informatics (e.g., TB-Profiler software)^6^.

In UK, the UK Health Security Agency (UKHSA) and some hospitals now use NGS-based characterisation as a clinical standard of care for TB management^8^. Thailand is seeking to adopt NGS as part of clinical care and surveillance, with government investment in genomics capacity. Recently, the WHO released the “genotype to phenotype” interpretation of NGS data^7,9^, but this approach needs to be adopted by National Tuberculosis Control Programmes (NTPs). NTP Guidelines developed in Thailand recommend using Mtb WGS to investigate cases involving clusters of TB patients or suspected outbreaks^10^, but to date the sample sizes have been small^11–13^. Challenges to wider adoption include limited funding, the need for sustained capacity strengthening in bioinformatics and laboratory infrastructure, and the complexity of data analysis and interpretation. Addressing these barriers is critical for integrating WGS into routine TB control efforts and realising its full potential for surveillance and outbreak investigation in the region. Genomics studies of Mtb from Thailand have revealed a dominance of lineage 2 strains^11^, and interactions with host genetics^14^. Our study analysed a large Mtb sample set from Thailand (n = 2,005; spanning 1994 to 2020), including 1,189 newly sequenced isolates, most isolates in the dataset were classified as MDR-TB, aims to identify mutations associated with drug resistance and uncover evidence of transmission. By establishing a baseline analysis of circulating strains, we seek to assist infection control teams embarking on using WGS to assist clinical and surveillance activities.

## Methods

### Sequence data

This study includes 2,005 M*. tuberculosis* whole-genome sequences obtained through convenience sampling in Thailand, predominantly from patients with drug-resistant TB, including MDR-TB and XDR-TB forms. Of these, 1,189 (59.3%) were recently gathered from Siriraj Hospital (years 2017–2020), a specialist TB hub for nationwide care, and 816 were retrieved from the European Nucleotide Archive (ENA) database (1994–2016)^11,15^. All sequence data were generated using the Illumina sequencing platform (see Table S1 for ENA accession numbers). Metadata, when available, included the year of collection, location, and phenotypic drug susceptibility tests (pDSTs) results. The pDSTs were conducted as part of routine TB laboratory processes using the standard agar proportion method on Lowenstein-Jensen medium. The number of isolates tested varied depending on the study, and the drugs included in this analysis were isoniazid (INH), rifampicin (RIF), ethambutol (EMB), streptomycin (STR), levofloxacin (LFX), moxifloxacin (MFX), amikacin (AMK), kanamycin (KAN), and linezolid (LZD). Pre-XDR and XDR have been defined under new WHO definitions^16^. For some isolates where XDR-TB could not be confirmed due to missing linezolid (LZD) and bedaquiline (BDQ) data, we classified them as pre-extensively drug-resistant-plus TB (Pre-XDR+ TB). The new study was approved by the Mahidol University ethics committee (SIRB 510/2561, MOPH EC 16/2562, Makarak EC30/2561).

### Bioinformatic and phylogenetic analysis

All raw sequence files were aligned to the H37Rv reference genome (accession number NC_000962.3) using bwa-mem software. High-quality single nucleotide polymorphism (SNP) and insertion/deletion (indel) variants were called using samtools and GATK software and merged into a variant FASTA file using fastq2matrix. To ensure high-quality variant calls, we applied the following quality control criteria to all samples: ≥ 90% of reads mapped, median sequencing depth ≥ 30-fold, and < 10% of the genome with coverage below tenfold, based on summary statistics from the bamstat output. Additionally, variants were retained only if the alternate allele frequency was ≥ 75%, and *pe/ppe* gene regions were not excluded, but results from these regions were interpreted with caution due to known mapping challenges^17^. TB-Profiler software (v6.2.2) was used to determine isolate sub-lineages and genotypic drug resistance across 18 antibiotics^6^. The TB-Profiler drug resistance database incorporates mutations identified in the WHO catalogue as being associated with drug resistance in the *Mycobacterium tuberculosis* complex^9^. Phylogenetic trees were constructed from the multi-sample FASTA file, using the maximum likelihood method implemented in IQ-TREE software (v2.2.6) with a set of 75,060 genome-wide SNPs. The trees were subsequently visualised using iTOL (v6). The geographic distribution of isolates was mapped using QGIS software (v3.36.0-Maidenhead). For the transmission analyses, pairwise SNP distances were calculated using the snp-dists tool within the fastq2matrix pipeline. A Gaussian Mixture Model (GMM) was applied to the distribution of pairwise SNP distances between isolates to identify natural groupings in the data. This unsupervised clustering approach enabled the determination of an appropriate SNP distance cut-off for defining putative transmission clusters, accounting for the population structure and time span of our dataset. This method avoids the need for an arbitrary SNP threshold and allows for context-specific identification of transmission links. Timed phylogenetic trees were constructed for the largest clusters using BEAST2 (v2.7.6)^18^, applying a strict molecular clock, a coalescent constant population model, and a General Time Reversible (GTR) substitution model. The Markov chain Monte Carlo (MCMC) analysis was run for 100 M iterations, sampling every 10,000^th^ step. Temporal signals were assessed using TempEst, which showed a positive correlation between genetic divergence and sampling time and a moderate root-to-tip correlation (R^2^ = 0.5241). Analysis settings were based on approaches described previously^19^. A resulting maximum clade credibility (MCC) tree was annotated using Tree annotate software (v2.7.6). The Transphylo package (v1.4.10) was used to determine the direction of any transmission events using the default settings. The transmission graph was inferred and formatted using the tgv web tool^20^. The frequency of SNP and indel variants is compared to a global dataset (n = 50,723) curated in the TB-Profiler database.

### Statistical analysis

Logistic regression models were employed to determine associations between transmissibility outcomes (whether an isolate was part of a transmission cluster), lineage and genotypic drug resistance profiles. Additionally, SNP loci associated with transmissibility were assessed using linear mixed models implemented in the Genome-wide Efficient Mixed Model Association (GEMMA) software (v0.98.5). This approach, related to genome-wide association study (GWAS) methods, accounted for the kinship matrix, incorporating SNPs, lineage, and drug resistance to control for relatedness among isolates^21^. Associations between geographical and SNPs distances of isolates were assessed using non-parametric Kruskal–Wallis tests and Spearman’s correlations. Statistical analyses were performed in R software (4.4.1).

## Results

### Study population

**Fig. 1:**
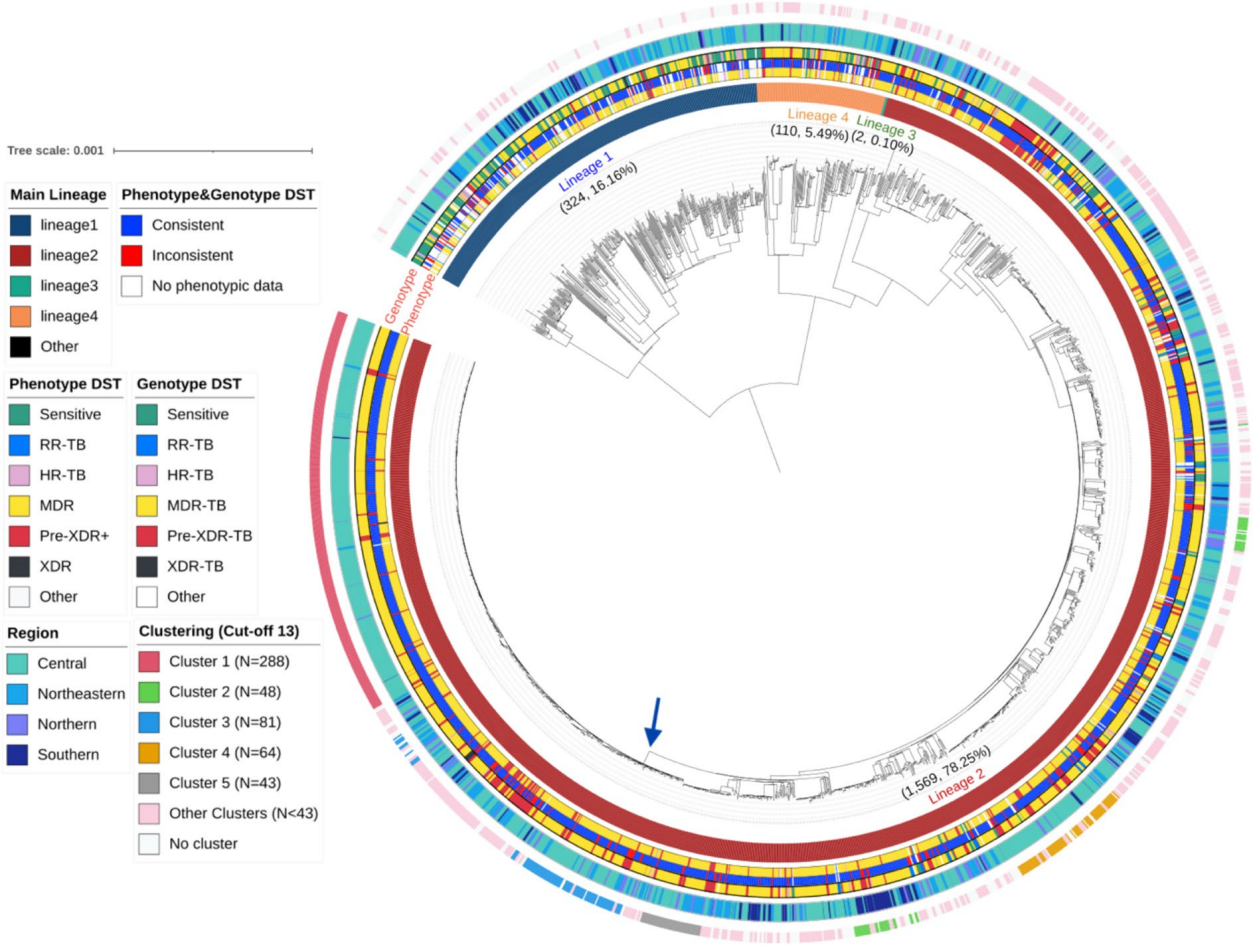
Phylogenetic Tree of 2,005 Mtb isolates from Thailand, 1994–2020. The scale bar indicates 0.01 substitutions per site SNP. The outer ring highlights isolates within the transmission cluster, defined by an SNP distance cut-off of 13. Genotypic DSTs refer to WGS-based drug resistance profiles. Blue arrow indicates a cluster of highly similar Mtb isolates within lineage L2 (n = 531).

A total of 2,005 Mtb samples from Thailand, collected between 1994 and 2020, were included in this study (Table 1). A high number of Mtb isolates (838/2005, 41·8%) were collected between 2013 and 2017, spanning 4 regions of Thailand (Central, Northeastern, Northern, Southern). Most samples originated from the Central region (1299/2005, 64·8%), with Kanchanaburi province contributing the largest number (558/2005, 27·8%). This dataset includes a high proportion of drug-resistant TB cases. Based on Thailand’s national TB reports, 6,992 laboratory-confirmed MDR/RR-TB cases were reported between 2014 and 2020^22^. Our dataset includes 594 MDR/RR-TB isolates from the same period, covering approximately 8.5% of the national burden during those years. The majority of Mtb strain-types were lineage L2 (East-Asian; 1569/2005, 78·3%), followed by L1 (Indo-Oceanic, 324/2005, 16·2%), L4 (Euro-American, 110/2005, 5·5%) and L3 (East-African-Indian, 2/2005, 0·1%). The most prevalent sub-lineage, L2.2.1 (Beijing strain-type) accounted for most isolates (1317/2005, 65.7%). Across the 2,005 isolates, 75,060 high-confidence SNPs were identified, and the resulting phylogenetic tree revealed the expected grouping of isolates by (sub-)lineage (Fig. 1). A principal component analysis (PCA) using the SNPs supported the lineage-based clustering patterns in the phylogenetic tree (Figure S1).

**Table 1:** Demographic data and genotypic drug-resistant Profiles of 2,005 TB cases, Thailand, 1994–2020.

Characteristics		N	%
Year	1994–2002	45	2·2
	2003–2007	378	18·9
	2008–2012	592	29·5
	2013–2017	838	41·8
	2018 +	152	7·6
Region	Central	1299	64·8
	Northeastern	379	18·9
	Northern	145	7·2
	Southern	182	9·1
Province	Kanchanaburi	558	27·8
	Bangkok	297	14·8
	Nakhon Ratchasima	108	5·4
	Buri Ram	104	5·2
	Other	938	46·8
Drug resistance	Sensitive	233	11·6
	RR-TB	39	1·9
	HR-TB	53	2·6
	MDR-TB	1349	67·3
	Pre-XDR TB	293	14·6
	XDR-TB	5	0·3
	Other	33	1·7
Anti-TB drug resistance	INH	1686	84·1
	RIF	1693	84·4
	EMB	1095	54·6
	STR	1212	60·5
	PZA	812	40·5
	LFX	305	15·2
	MFX	305	15·2
	AMK	108	5·4
	KAN	128	6·4
	ETO	875	43·6
	BDQ	14	0·7
	LZD	0	0
Lineage	L2	1569	78·2
	L1	324	16·2
	L4	110	5·5
	L3	2	0·1

RR-TB Rifampicin-Resistant tuberculosis; HR-TB isoniazid-monoresistant; MDR-TB, Multidrug-Resistant; Pre-XDR TB Pre-Extensively Drug-Resistant; XDR-TB Extensively Drug-Resistant; INH Isoniazid; RIF Rifampicin; EMB Ethambutol; STM Streptomycin; PZA Pyrazinamide; LFX, Levofloxacin; MFX, Moxifloxacin; AMK Amikacin, KAN Kanamycin; ETO Ethionamide; PAS Para-aminosalicylic acid; CAP Capreomycin.

### Genotypic drug resistance

**Fig. 2:**
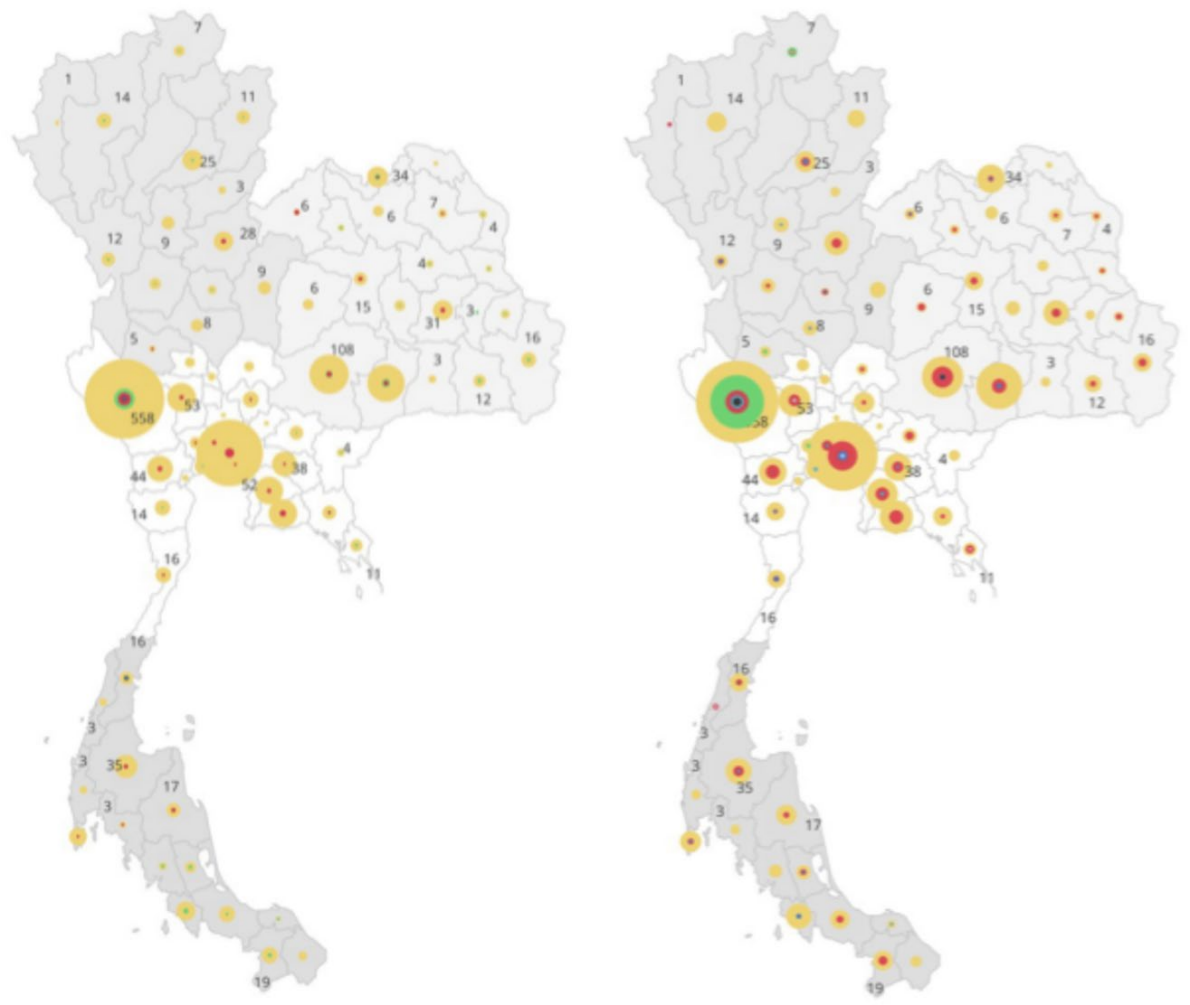
Geographic Distribution of Mtb Lineages and Genotypic Drug resistance profiles in Thailand (N = 2,005 isolates). The size of the diagrams corresponds to the number of isolates; however, the size of smaller diagrams has been adjusted to enhance visibility, as some provinces reported very few TB cases.

Genotypic drug resistance profiling (Table 1) revealed that 88·4% of isolates (1772/2005) were resistant to at least one anti-TB drug, with the highest resistance observed for isoniazid (1686/2005, 84·1%), rifampicin (1693/2005, 84·4%), and streptomycin (1212/2005, 60·5%). WGS-based profiling indicated that 67·3% (1349/2005) of isolates were classified as MDR-TB, 14·6% (293/2005) as pre-XDR-TB, 11·6% (233/2005) as sensitive, and 0·2% (5/2005) as XDR-TB (Table 1). The geographic distribution of lineages and genotypic drug resistance categories across Thailand, suggested that MDR-TB was present across most regions (Fig. 2).

The most common mutations linked to drug resistance were *katG* Ser315Thr (71·6%, isoniazid), *rpsL* Lys43Arg (46·4%, streptomycin), *rpoB* Ser450Leu (46·0%, rifampicin), *ethA* c.639_640delGT (26·6%, ethionamide), *embB* (Gly406Asp) (16·0%, ethambutol), *pncA* Ile31Thr (16·0%, pyrazinamide), and *embB* Met306Val (14·4%, ethambutol). All mutations have been observed in the TB-Profiler database, which includes global isolates (N = 50,723). However, there were differences in allele frequencies. For example, the *pncA* Ile31Thr mutation, linked to pyrazinamide resistance, was over 288 times more frequent than in the global dataset. Similarly, the *ethA* (c.639_640delGT) mutation, associated with ethionamide resistance, occurred at a frequency more than 140 times higher than in the global collection (Table 2). A detailed catalogue of identified mutations associated with drug resistance is presented (Table S2). The degree of resistance to drugs may be underestimated, especially as functional genotype–phenotype mutations are being found and may need to be included in updates to databases. For example, the *ddn* Gly81Ser mutation has recently been associated with delamanid resistance^23^. This mutation was identified in 22 isolates (1.1%), including 6 Mtb isolates from Kanchanaburi (2005–2017) in the ENA database^11,15^, and 16 isolates collected between 2007 and 2014 from Kanchanaburi, Bangkok, Ratchaburi, Suphan Buri, and Chachoengsao -  all located in or around the central region of Thailand.

**Table 2:** Summary of Anti-TB Drug Resistance Mutations in 2,005 Isolates.

Anti-TB drugs	Gene name	Changes	Our study N = 2,005	Our study (%)	Global (%) N = 50,723^1^
INH	*katG*	Ser315Thr	1436	71·62	26·55
	*inhA*	c.−777C > T	182	9·08	4·06
	*inhA*	Ser94Ala	29	1·45	0·62
	*katG*	Ser315Asn	25	1·25	0·53
RIF	*rpoB*	Ser450Leu	923	46·03	18·80
	*rpoB*	His445Tyr	209	10·42	1·21
	*rpoB*	Asp435Val	114	5·69	2·30
	*rpoB*	His445Asp	107	5·34	1·04
EMB	*embB*	Gly406Asp	320	15·96	0·78
	*embB*	Met306Val	261	13·02	7·73
	*embB*	Met306Ile	236	11·77	5·94
STR	*rpsL*	Lys43Arg	931	46·43	13·49
	*rpsL*	Lys88Arg	74	3·69	2·55
	*rrs*	n.514A > C	34	1·70	2·53
	*gid*	c.102delG	30	1·50	1·25
PZA	*pncA*	Ile31Thr	289	14·41	0·05
	*pncA*	Ile90Ser	31	1·55	0·05
	*pncA*	c.−11A > G	24	1·20	0·90
LFX	*gyrA*	Asp94Gly	119	5·94	4·30
	*gyrA*	Ala90Val	88	4·39	3·15
	*gyrA*	Asp94Ala	39	1·95	1·18
MFX	*gyrA*	Asp94Gly	119	5·94	4·30
	*gyrA*	Ala90Val	88	4·39	3·15
	*gyrA*	Asp94Ala	39	1·95	1·18
AMK	*rrs*	n.1401A > G	98	4·89	4·71
	*eis*	c.−14C > T	8	0·40	0·48
KAN	*rrs*	n.1401A > G	98	4·89	4·71
	*eis*	c.−10G > A	17	0·85	0·72
	*eis*	c.−14C > T	8	0·40	0·48
ETO	*ethA*	c.639_640delGT	534	26·63	0·18
	*inhA*	c.−777C > T	182	9·08	4·06
	*inhA*	Ser94Ala	29	1·45	0·62
PAS	*folC*	Ser150Gly	91	4·54	0·31
	*thyX*	c.−16C > T	76	3·79	0·75
	*folC*	Glu40Gly	29	1·45	0·17
CAP	*rrs*	n.1401A > G	98	4·89	4·71
	*tlyA*	c.52_53dupCG	3	0·15	< 0·001

There could be > 1 mutation associated with resistance to a single drug. ^1^The global percentage of anti-TB drug resistance mutations identified by TB-Profiler database; INH, Isoniazid; RIF, Rifampicin; EMB, Ethambutol; STR, Streptomycin; PZA, Pyrazinamide; LFX, Levofloxacin; MFX, Moxifloxacin; AMK, Amikacin, KAN, Kanamycin; ETO, Ethionamide; PAS, Para-aminosalicylic acid; CAP, Capreomycin.

### Concordance with phenotypic DST

Phenotypic DST assays were performed on a subset of the samples (n = 1,826) (Table S3). Isoniazid exhibited the highest resistance rate at 95·6% (1745/1826), followed by rifampicin at 94·7% (1730/1826), streptomycin at 61·8% (1113/1801), ethambutol at 44·0% (727/1653), and levofloxacin at 12·2% (199/1602). All other resistance rates were below 10%, with moxifloxacin at 8·6% (95/1101), kanamycin at 7·5% (127/1687), amikacin at 6·9% (81/1,172), and linezolid at 0·4% (4/1,097). Overall, 1,522 isolates (75·9%) were classified as MDR-TB, followed by 201 isolates (10·0%) with Pre-XDR+ TB, and 59 isolates (2·9%) being pan-sensitive. XDR-TB was identified in 3 isolates (0·2%) (Table S3).

The overall concordance rate between phenotypic DSTs and genotypic drug resistance was 91·1% (11,522/12,640 tests). Concordance exceeded 88% for all drugs except ethambutol (71·7%), with rates of 96·1% for isoniazid, 96·9% for rifampicin, 97·5% for amikacin, 91·9% for levofloxacin, 89·7% for streptomycin, 96·8% for kanamycin, and 88·6% for moxifloxacin (Table S4). Assuming phenotypic DST results as the gold standard, the sensitivity and specificity for isoniazid and rifampicin, which are critical for defining MDR-TB, were approximately 96%. The remaining drugs demonstrated high levels of sensitivity and specificity, ranging from 70 to 99%, with ethambutol showing a notably lower specificity of 60·4%.

Despite the potential for DST errors, we identified 88 mutations that may account for cases of phenotypic resistance with genotypic susceptibility. These include 24 mutations linked to isoniazid (*katG* 22, *aphC* 2), 7 to rifampicin (*rpoB* 5, *rpoC* 2), 16 to ethambutol (*embA* 7, *embB* 8, *embC* 1), and 19 to streptomycin (*gid* 11, *rpsL* 2, *rrs* 6) (Table S5). Some isolates with known drug resistance mutations also harboured additional mutations that might act as compensatory mechanisms (e.g., *rpoB* Lys37Arg, *rpoB* Met655Thr; *rpoC* Gly1198Ser). At the composite resistance level (e.g., MDR-TB), the concordance between phenotypic DSTs and genotypic profiles was high at 87·4% (1589/1819) (Table S6). Most discordances (181/230, 78·7%) were linked to variations in MDR-TB classification, particularly cases where genotypic profiling identified mutations meeting the new definition of (pre-)XDR-TB (114/181). Instances of discordance where DST indicated susceptibility, but genotypic profiles suggested resistance (8/59) likely reflected phenotypic errors. These cases included several well-established resistance mutations, such as *embB* Gly406Asp (ethambutol), *gyrA* Asp94Ala (moxifloxacin), and *eis* c.−10G > A (kanamycin) (Table S7).

### Transmission cluster analysis

The phylogenetic tree revealed clusters of Mtb isolates within the same lineage that were highly similar, and we sought to investigate whether these are indicative of transmission events (Fig. 1). An analysis of the pairwise SNP distance distribution across 2,005 isolates (median 452 SNPs; range: 0 to 2,369 SNPs) (Figure S2) suggested a cut-off of 13 SNPs to define potential transmission links. At this cut-off, we identified 206 transmission clusters (total n = 1,265 (63·1%); median size: 2, range: 2–288), including 190 clusters with 2–9 isolates, 15 clusters with 10–100 isolates, and one large cluster containing 288 isolates (Figure S3**; **Table S8). Using a 5-SNP cutoff found a similar number of transmission clusters (201). As expected, each cluster was homogenous for sub-lineage. The most prevalent sub-lineage was L2.2.1 (131/206 clusters; total n = 1,317), followed by L1.1.1 (10/206 clusters; total n = 159). The 1,265 Mtb isolates in clusters were identified in every region of Thailand, with the majority classified as MDR-TB and pre-XDR-TB (1,178/1,265, 93·1%). The clusters span several years (median 2: range 1–18 years), with a notable presence from 2005 to 2017. Within the set of 16 clusters comprising 10 or more isolates (Table S8), distinct geographical distributions were observed. For example, one cluster includes 22 isolates, all classified as L2.2.1 and pre-XDR-TB, originating from the central region, specifically Kanchanaburi, Ratchaburi, Suphanburi, and Bangkok, with samples collected between 2005 and 2017, and having the *ddn* Gly81Ser mutation. Another cluster consists of 48 isolates classified as L2.1.1, predominantly from southern Thailand, with 43 (93·8%) identified as MDR-TB; these samples were collected from 2001 to 2020. Additionally, a cluster of L4.2.2 contains 12 isolates, mainly from the central region, with 9 (75%) classified as MDR-TB and 3 (25%) as pre-XDR-TB, with samples collected from 2003 to 2017. Overall, most clusters included isolates from both the newly sequenced and older public datasets (Figure S4), highlighting the persistence of lineages L1, L2, and L4 over time. To further explore the geographical spread of these clusters, we assessed the correlation between geographic and SNP distances (Figure S5). The analysis showed a small but significant positive trend (Kruskal–Wallis P < 0·001) with a weak positive correlation (Spearman’s rho = 0·046).

A logistic regression analytical approach was used to identify factors associated with an increased risk of being in a transmission cluster. Compared to lineage L1, being an isolate in L2 (odds ratio [OR] 7·41, 95% CI [5·34, 10·29], p < 0·001) or L4 (OR 2·03, 95% CI [1·23, 3·36], p < 0·05) had increased odds. Whilst, compared to sensitive strains, those with MDR-TB (OR 5·82, 95% CI [3·93,8·61], p < 0·001) or pre-XDR-TB (OR 10·98, 95% CI [6·72,17·94], p < 0.001) were significantly associated with an increased risk of clustering. Further, those isolates from Central and Northeastern regions were more likely to cluster compared to the Northern region (OR > 2·5, p < 0·001) (Table S9).

Through a genome-wide analysis using common SNPs (minor allele frequency (MAF) > 0·01), we identified 4 loci that were significantly associated with being in transmission clusters, located in the genes *katG* (Rv1908c), *folC* (Rv2447c), *ppe8* (Rv0355c), and *folK* (Rv3606c) (P < 0·001) (Table S10). Among loci with odds ratios greater than one, we detected the *katG* Ser315Thr mutation - the most common INH resistance mutation^24^ as most clusters are linked to at least MDR-TB (OR 7·94, 95% CI [6·39,9·87], p < 0·00001). The *folC* (*Rv2447c*) S150G mutation, involved in the production of para-aminobenzoic acid (pABA) in the folate biosynthetic pathway^25^, was found in lineages L2 and L4 (OR 6·5, 95% CI [3·13,13·51], p < 0.00001). Additionally, the *ppe8* (*Rv0355c*) V2309I mutation was also associated with clustering (OR 2.81, 95% CI [1·05,8·28], p < 0.05), this gene linked to adaptations in response to host defence mechanisms^26^. These mutations were observed across multiple clusters, including *katG* Ser315Thr (147 clusters), *folC* S150G (6 clusters), and *ppe8* V2309I (5 clusters) (Figure S4). Like *folC*, the *folK* (*Rv3606c*) locus is associated with folate metabolism. While the *folK* Gln47His mutation has an odds ratio below one, there is no strong evidence of compensatory effects (Figure S4).

### Investigation of the largest cluster

**Fig. 3:**
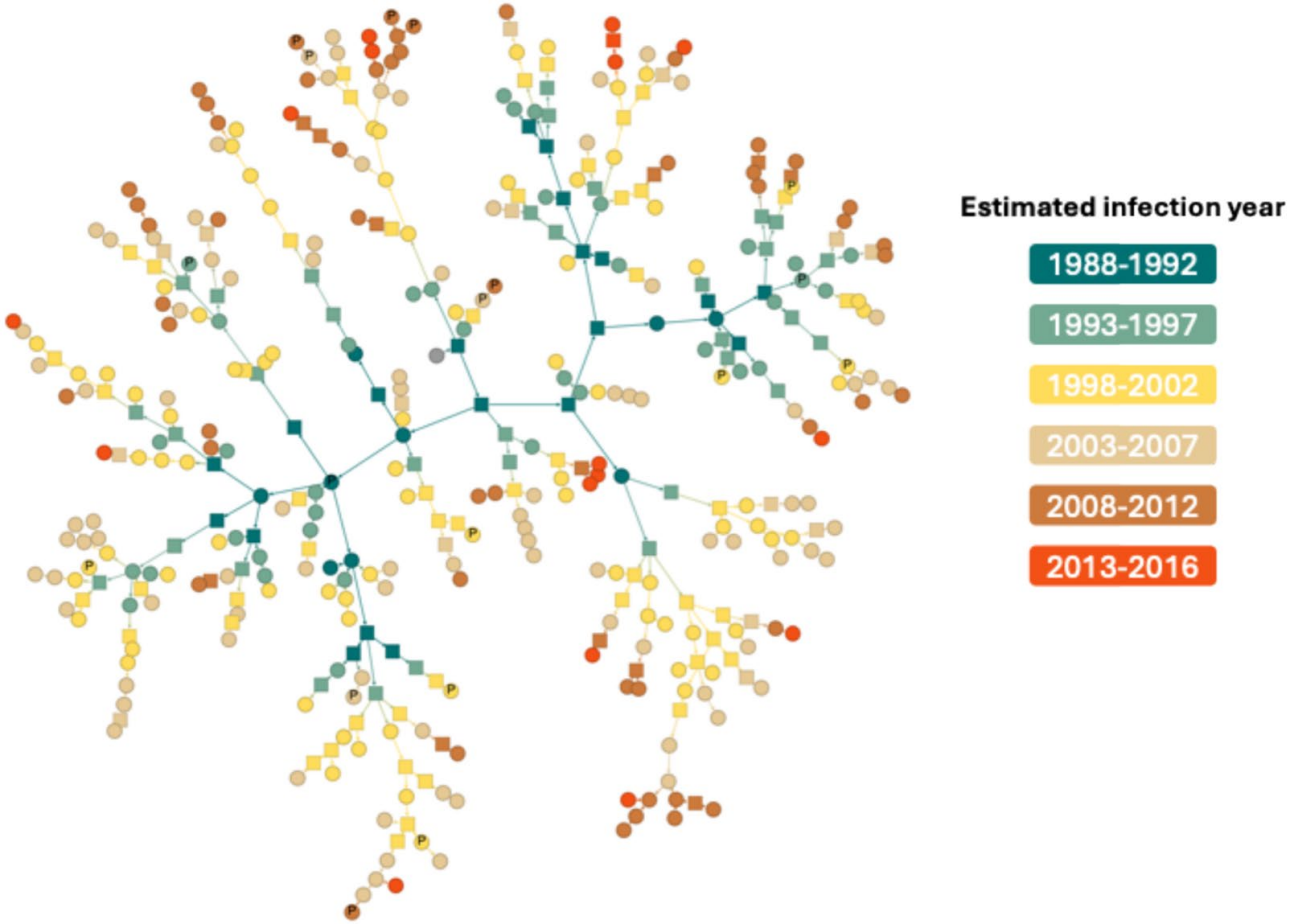
The maximum a posteriori (MAP) transmission tree generated from TransPhylo results, showing the transmission dynamics of TB cases. Nodes represent hosts, coloured according to the estimated infection year intervals: 1988 (1988–1992), 1993 (1993–1997), 1998 (1998–2002), 2003 (2003–2007), 2008 (2008–2012), and 2013 (2013–2016). Squares denote unsampled cases, while grey nodes represent the initial infection source. Nodes labelled with “P” represent Pre-XDR-TB cases, while unlabelled circles indicate MDR-TB cases.

The time-calibrated phylogenetic tree of the largest cluster was analysed using BEAST2 software. This cluster comprises 288 isolates, all belonging to sublineage L2.2.1, with 96·9% originating from the central region and 93·8% classified as MDR-TB. The geographic distribution is shown in Figure S6, and additional details for clusters with ≥ 10 isolates are provided in Table S8. This analysis estimated the mutation rate to be 1·10 × 10^–7^ substitutions per site per year (95% highest posterior density (HPD) interval: 9·89 × 10^–8^ to 1·22 × 10^–7^), which corresponds to ~ 0·48 substitutions per genome per year (HPD: 0·44 to 0·53), and in keeping with estimates for L2 from other settings^27,28^. The estimated time of the most recent common ancestor is 1989 (95% HPD: 1984–1994), which is plausible given the known roll-out of the anti-TB drugs. All parameters had an effective sample size score greater than 200. The TransPhylo package was used to reconstruct the transmission history among cases, offering detailed insights into the source of infections within the studied population. Using maximum a posteriori estimates, we identified the most probable transmission pathways (Fig. 3), which revealed the evolution of Mtb, including from MDR-TB to pre-XDR-TB, through time. Based on this reconstruction, the model inferred a total of 420 transmission events within the largest cluster, of which 132 (31.4%) were attributed to unsampled individuals. These unsampled cases likely represent individuals who were infected but not captured in our dataset due to limitations in retrospective sampling, diagnostic coverage, or sequencing availability. (Figure S7). By applying a probability threshold of > 0.2, the large cluster was subdivided into 83 smaller sub-clusters. This breakdown facilitated more detailed investigation of each sub-cluster, allowing us to visualise potential transmission pathways (i.e., who-infected-whom), integrate drug resistance profiles and collection years, and assess epidemiological plausibility using available metadata to guide further contact investigations by infection control teams (Figure S8). We also compared the Thai clusters to a reference set of 154 representative global Beijing strains^29^, revealing notable similarities (Figure S9).

## Discussion

The WHO classifies Thailand as a high-burden TB country. This study presents a WGS analysis of 2,005 Mtb isolates from Thailand, spanning three decades (1994–2020), to examine circulating strains, drug resistance profiles, and transmission dynamics. The dataset includes isolates from previous studies^11,15^ and new data from Siriraj Hospital, with most samples originating from cases involving drug resistant TB, of which over 60% classified as MDR-TB. The frequency distribution of randomly recruited Mtb isolates in Thailand predominantly comprises L1 and L2^13^. Our findings reveal a high prevalence of L2, particularly the Beijing sub-lineage (L2.2.1). This aligns with prior studies reporting that L2 is the main lineage related to clusters of outbreaks and drug-resistant isolates in Thailand, while L1 is more prevalent in general Mtb cases^13^. Although our comparative analysis with global MDR clusters is limited, our findings indicate that the dominant MDR clusters in Thailand align with the global expansion of L2, sharing similar underlying resistance mutations. A more detailed phylogenetic and cluster comparison with global MDR genomes^6^ would be valuable in future studies to explore this further. The geographic patterns observed suggest that specific lineages may be driving local transmission, especially in densely populated regions such as the Central area.

The genotypic drug resistance profiles generated by TB-Profiler software had high concordance with phenotypic DST, confirming its reliability. Since most isolates were MDR-TB, resistance rates detected through both phenotypic DST and genotypic resistance profiling were highest for isoniazid and rifampicin. Discrepancies between phenotypic and genotypic approaches may arise due to DST laboratory errors, limitations in WGS’s ability to capture certain mutations, or gaps in the drug resistance mutation database used for annotation. We identified 88 low-frequency mutations that explained phenotypic resistance and genotypic sensitivity inconsistencies, including 66 linked to four drugs: isoniazid (24), rifampicin (7), ethambutol (16), and streptomycin (19). These mutations, confirmed using phenotypic DST, were not lineage-specific and occurred at low frequencies in a dataset of 50,723 global Mtb samples. As such, they should be considered for inclusion in the TB-Profiler database. At the DR profile level, samples initially classified as MDR-TB by phenotypic DST were re-categorised genotypically in over 4·9% of cases to a lower resistance level (sensitive, RR-TB, or HR-TB) and in 7·5% of cases to a higher resistance level (pre-XDR-TB or XDR-TB). This reclassification is a crucial consideration, as treatment for each type of TB is specific, with distinct drug regimens and durations^30^. Inappropriate treatment could lead to further complications. A particularly concerning finding was the presence of the *ddn* Gly81Ser mutation, which is associated with delamanid resistance^23^, even in isolates predating the drugs rollout. Intrinsic resistance to delamanid and pretomanid - both sharing a similar mechanism of action -could undermine the effectiveness of the latest BPaLM regimen, especially as bedaquiline resistance continues to rise. Although this mutation may be lineage-associated, its functional impact on drug resistance has been experimentally validated, supporting its biological relevance. These findings emphasise the importance of using WGS data to identify mutations potentially associated with drug resistance, even in cases where phenotypic DST results indicate sensitivity.

The phylogenetic analysis of Mtb isolates revealed distinct clusters within the same lineage, suggesting potential transmission chains. Using a pairwise SNP distance cut-off of 13, we identified 206 transmission clusters comprising 1,265 isolates. These clusters ranged from small groups (190 clusters with 2–9 isolates) to larger networks (16 clusters with 10–100 isolates), including one exceptionally large cluster of 288 isolates, all highly homogenous for sub-lineage. The presence of transmission clusters in every region of Thailand, coupled with a substantial proportion of MDR-TB and pre-XDR-TB cases, underscores the widespread nature of drug-resistant TB across the country. Clusters spanned several years, with the majority arising between 2005 and 2017, reflecting ongoing transmission. Due to the convenient sampling method, it is possible that other isolates existed outside the given collection years. Clusters with higher percentages in more recent years (e.g., 2012–2017) may indicate ongoing transmission. It suggests that certain strains have persisted over time, reflecting continuous transmission of similar strains across years.

The study found that lineage, drug resistance, and geographic factors were significantly associated with the transmission dynamics of Mtb. Lineage L2 exhibited the highest transmissibility and adaptability, showing the strongest odds of clustering. Although less frequently involved in outbreaks than L2, L4 still presented an increased risk of clustering^31^, with both being modern strains. Drug resistance, particularly MDR-TB and pre-XDR-TB, further increased the risk of clustering. Geographic factors also played a key role, with isolates from the Central and Northeastern regions having higher risk of clustering. These findings can inform the design of tailored interventions aimed at high-risk areas and populations.

Using a GWAS approach, three loci in *katG*, *folC*, and *ppe8* were identified as associated with clustering or transmissibility, suggesting a potential increase in Mtb fitness and transmission. These loci have not been reported in similar analyses in Asia with comparable lineages^32,33^. Both the *katG* and *folC* genes are associated with drug resistance^25,32^. However, studies suggest that the increased transmission risk of drug-resistant tuberculosis is driven more by delays in diagnosis and treatment than by the inherent transmissibility of resistant strains^34^. The *ppe8* locus exhibits complexity, with deletions at the gene’s start in L1 strains and within the encompassed RD304 region in L2 strains^4^. Although Mtb GWAS can be used to identify associations between SNPs and transmissibility, some suggest incorporating host genomics into the analysis, such as through genome-to-genome approaches, for more comprehensive results^14^.

We identified a large cluster of 288 isolates and employed time-calibrated phylogenetic analysis using BEAST2 to reconstruct the outbreak’s transmission history from WGS data. We subsequently used TransPhylo to infer transmission patterns within the studied population. It enabled us to estimate the number of unsampled cases, revealing a hidden burden of TB transmission and suggesting that undetected infections may sustain ongoing transmission chains. To improve confidence in our inferences, we filtered out transmission probabilities below 0·2, reflecting the varied, convenience-based nature of sampling. Consequently, we could more reliably focus on smaller transmission clusters. Occasionally, we observed transmission patterns that appeared counterintuitive, such as transmission from Pre-XDR-TB to MDR-TB cases or from more recent years to earlier years (e.g., 2008 to 2006). These anomalies may reflect unsampled transmission links or uncertainties in determining the actual timing of infection. Specifically, our analysis relied on the year of sample collection rather than the precise year of infection, which can introduce discrepancies. By continually integrating updated WGS data, we can enhance the monitoring of patients and their transmission networks, thereby strengthening efforts to contain and prevent the spread of transmission clusters.

While our study shows that WGS is useful for studying drug resistance and TB transmission, several challenges remain before its routine implementation in TB control programs. These include the high cost of sequencing, delays in turnaround time in clinical settings, and the requirement for specialised personnel and infrastructure to support genomic data analysis and interpretation. Furthermore, effective integration into national TB surveillance systems necessitates standardised protocols, real-time data sharing, and clear guidance on how to act on genomic findings. Despite these challenges, WGS provides valuable insights by identifying undetected cases, transmission clusters, and resistance-associated mutations. These insights can support more targeted contact investigations, track the spread of high-risk strains, and inform treatment strategies in regions with ongoing transmission. Future efforts should aim to enhance WGS accessibility, streamline analytical workflows, and integrate host and pathogen genomic data to improve our understanding of TB transmission and support precision public health interventions.

In conclusion, this study provides a comprehensive analysis of WGS data from Mtb isolates in Thailand, offering valuable insights into circulating strains, drug resistance profiles, and transmission dynamics, particularly among drug-resistant TB cases. The findings highlight the critical role of integrating genomic and epidemiological data to deepen our understanding of Mtb and to guide targeted interventions for TB control. However, WGS alone cannot address all TB-related challenges. Sustained efforts combining WGS with strengthened public health strategies, enhanced surveillance systems, and multifaceted interventions are essential to effectively combat TB in Thailand and beyond.

## Supplementary Information

Supplementary Information 1.

Supplementary Information 2.

## Data Availability

Raw sequencing data are available in the European Nucleotide Archive (ENA). A complete list of accession numbers is provided in Table S1.
