# Supplementary material for: Genomic decoding of drug-resistant tuberculosis transmission in Thailand over three decades
Source: Sci Rep. 2025 Aug 13;15:29617. doi: 10.1038/s41598-025-15093-7 (PMC12344131; doi:10.1038/s41598-025-15093-7)
Supplement: Supplementary file 1 — Supplementary Information 1. [file 41598_2025_15093_MOESM1_ESM.pdf]

Supplementary Information

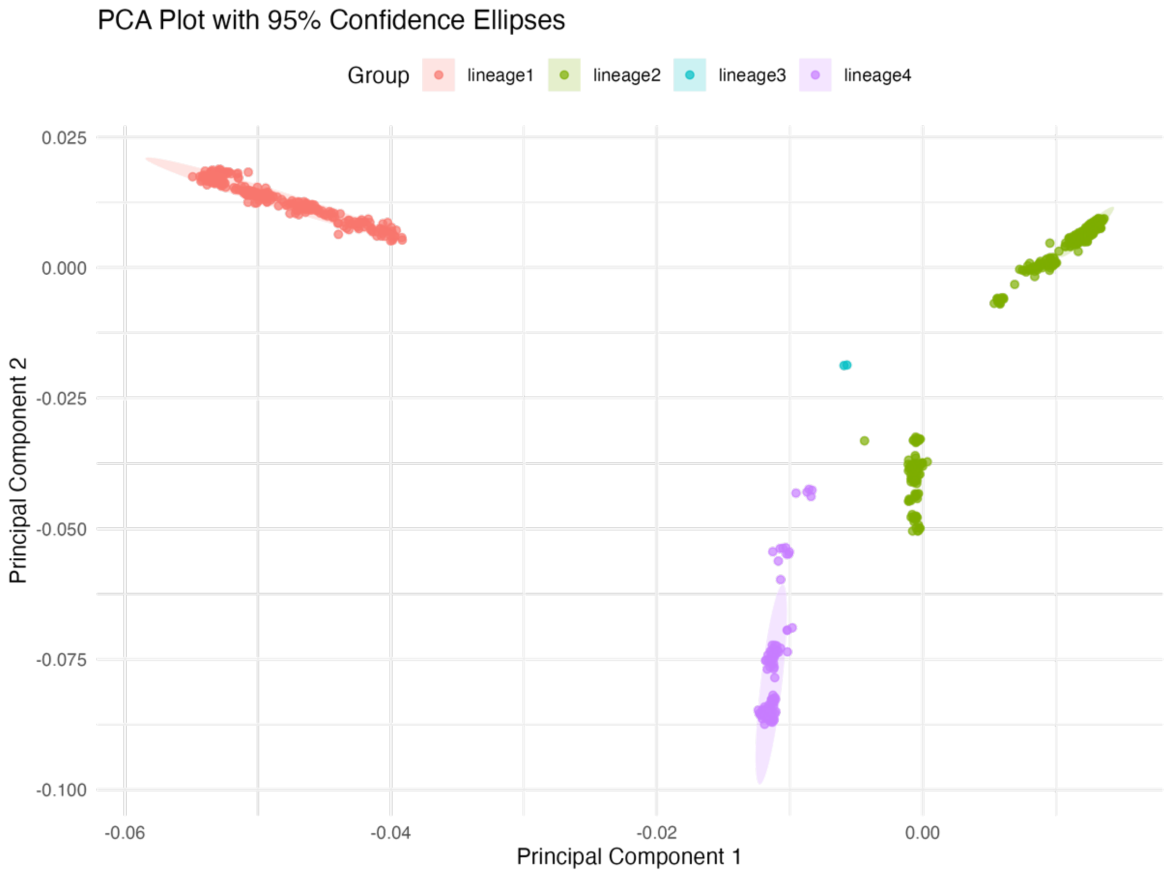

Figure S1. Principal component analysis (n=2,005)

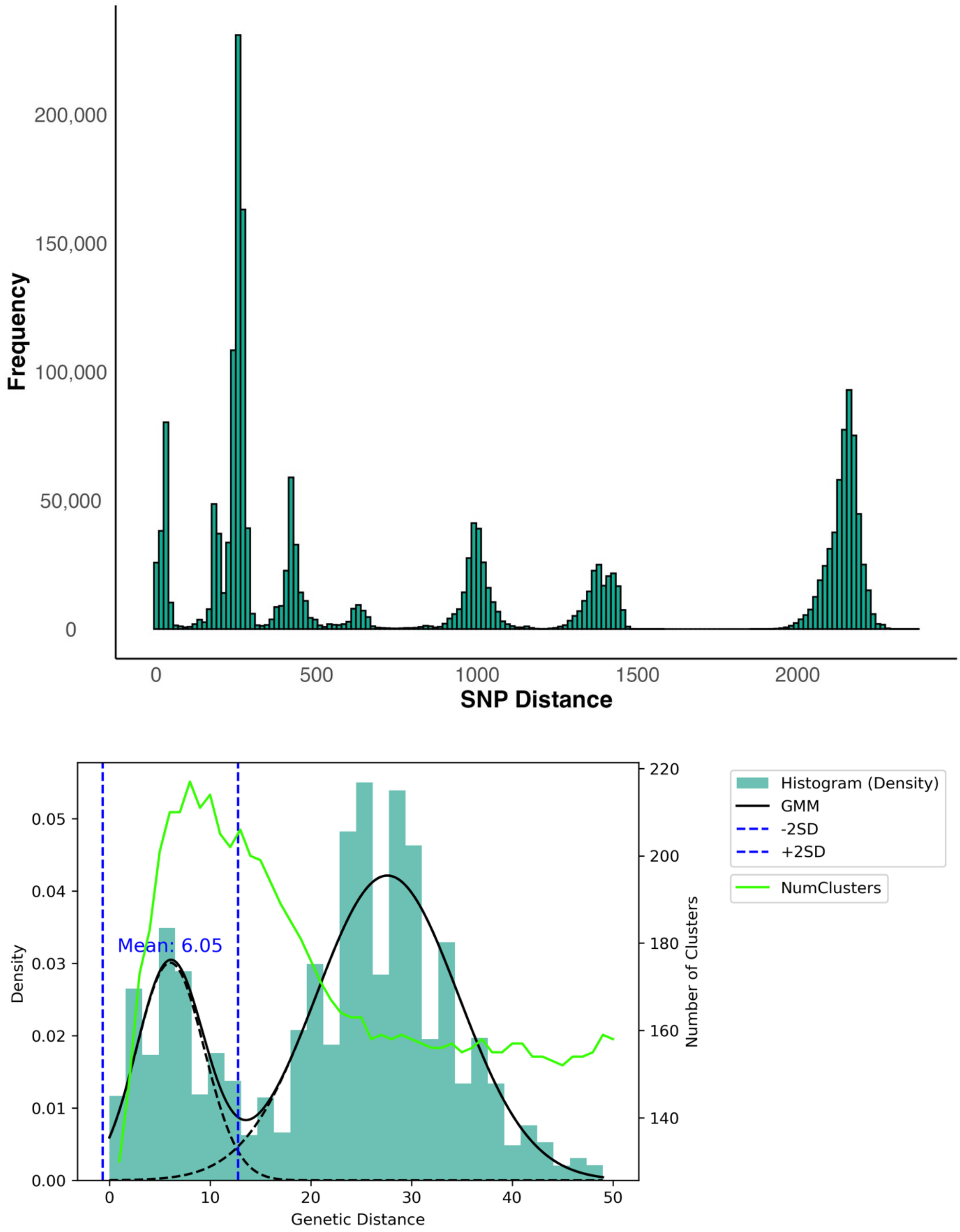

Figure S2. (top). Histogram of SNP Distances. (bottom). Distribution of Pairwise SNP Distances Less Than 50. GMM components are indicated. Dashed lines mark the boundaries separating the two components. The Gaussian Mixture Model (GMM) was used to estimate the means and standard deviations (SD) for these distributions. For the first component, the mean ± 2 SD was calculated as 6.05 ± 6.71. Utilising the mean plus 2 SD, we established a cutoff for clustering in the main analysis, resulting in a cutoff value of approximately 13 (estimated from 12.76).

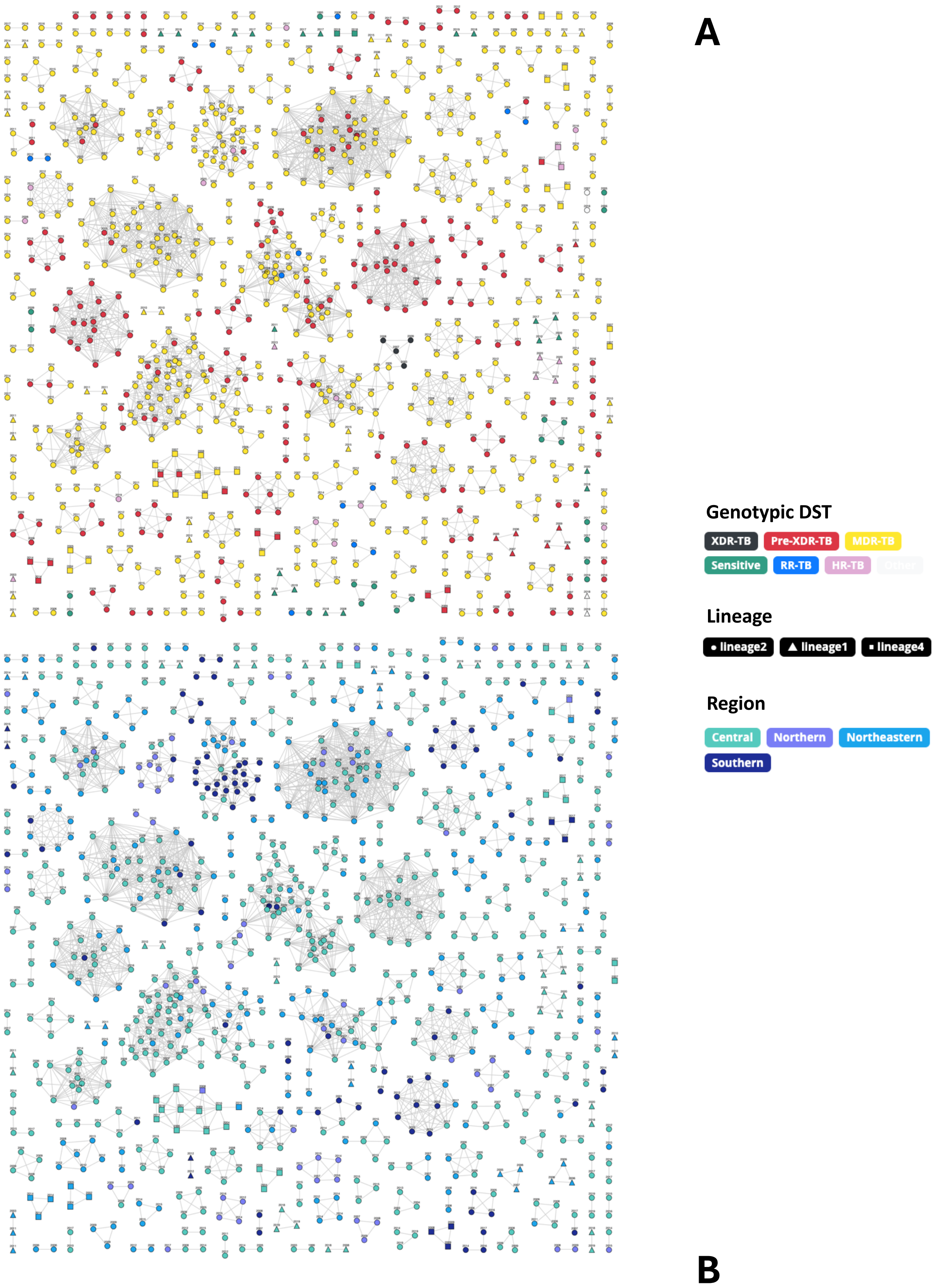

Figure S3. Transmission clusters were identified using an SNP distance cut-off value of ≤13, excluding the largest cluster (288 isolates). (A) Clusters coloured by genotypic drug resistance profile results and (B) clusters coloured by region of Thailand. The shape of each node represents different lineages.

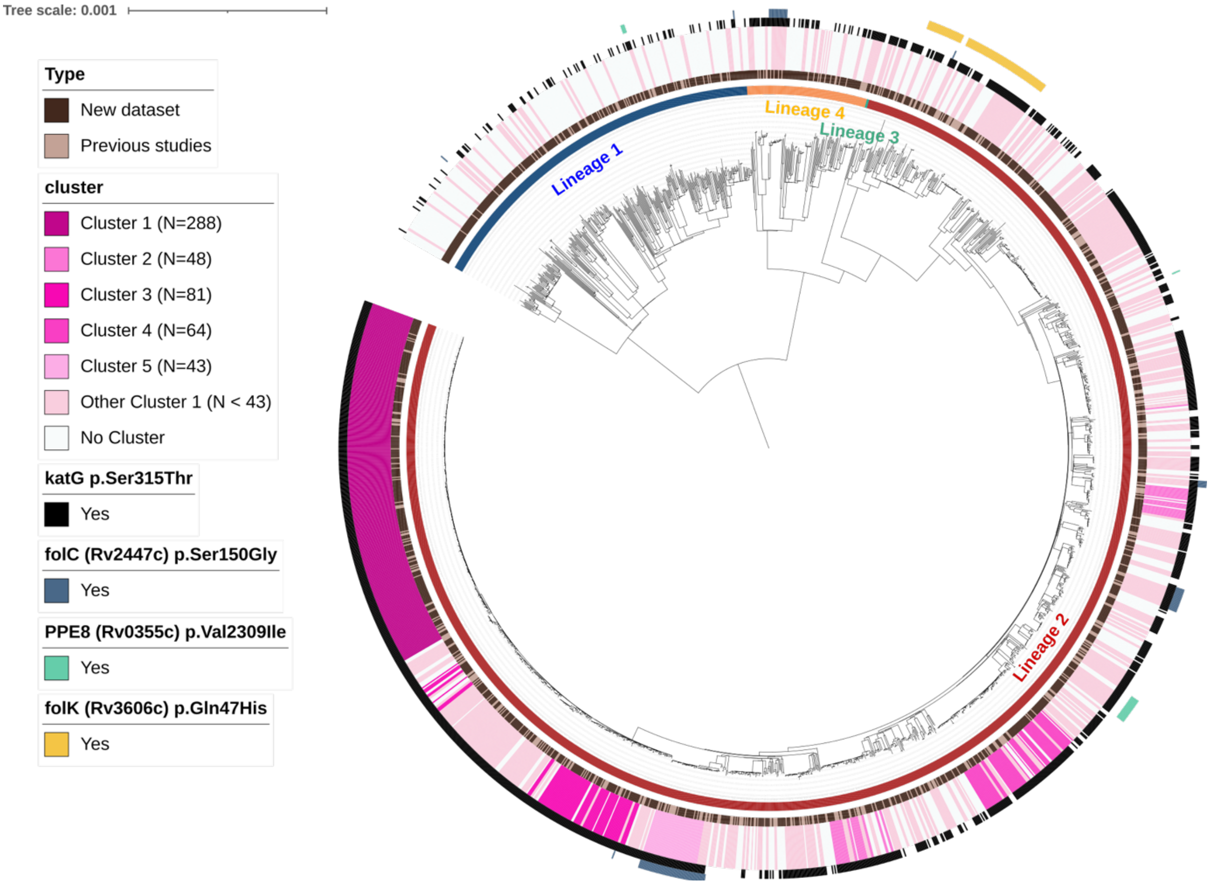

Figure S4. Phylogenetic Tree of 2,005 Mtb Isolates from Thailand, 1994–2020. The scale bar indicates 0.01 substitutions per site SNP. The tree displays data types and the presence or absence of the *katG* Ser315Thr, *folC* Ser150Gly, *ppe8* Val2309Ile and *folk* Gin47His mutations, within each cluster.

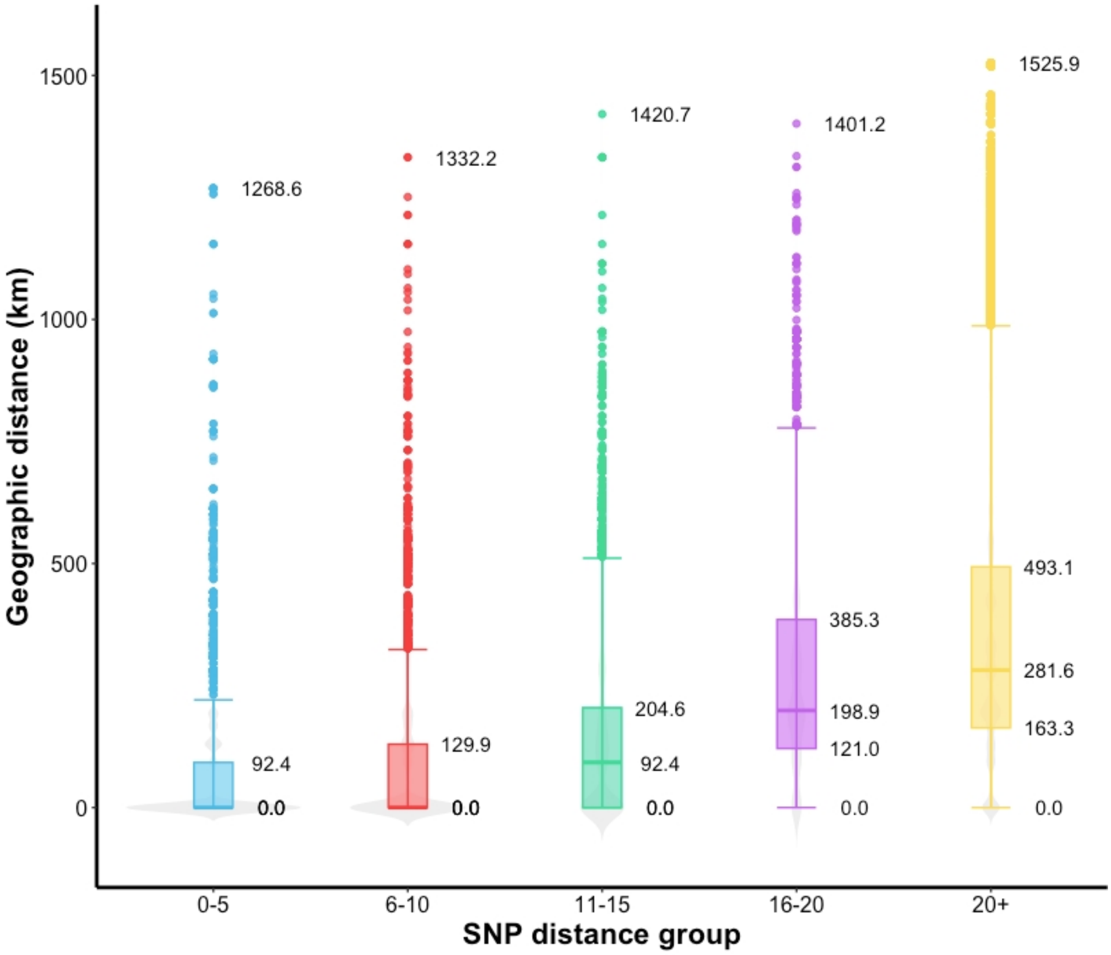

Figure S5. The boxplot displays the relationship between SNP and geographic distance.

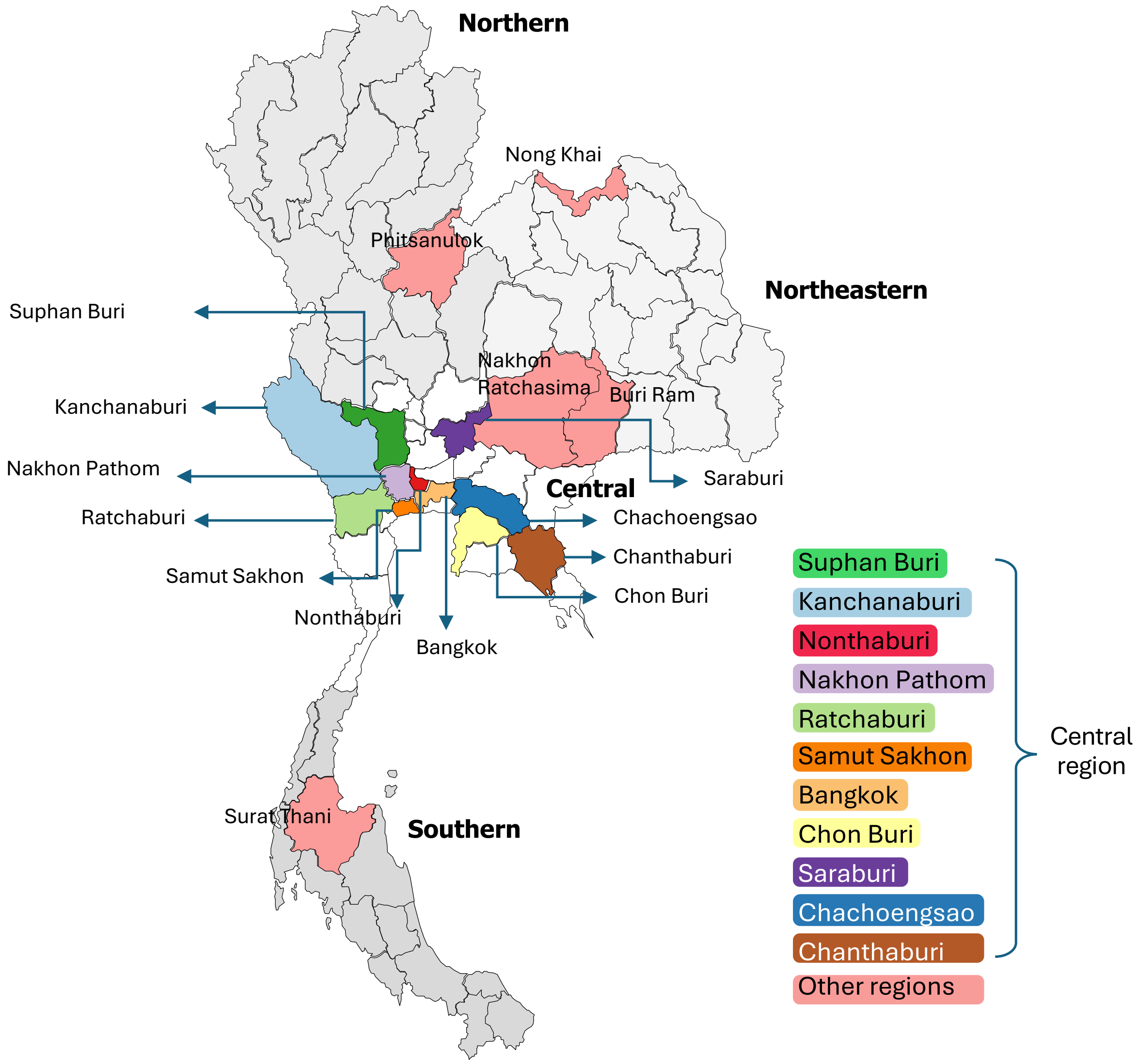

Figure S6. Distribution of the clusters of Mtb isolates in the largest cluster (SNP distance ≤13, N = 288 isolates), Thailand

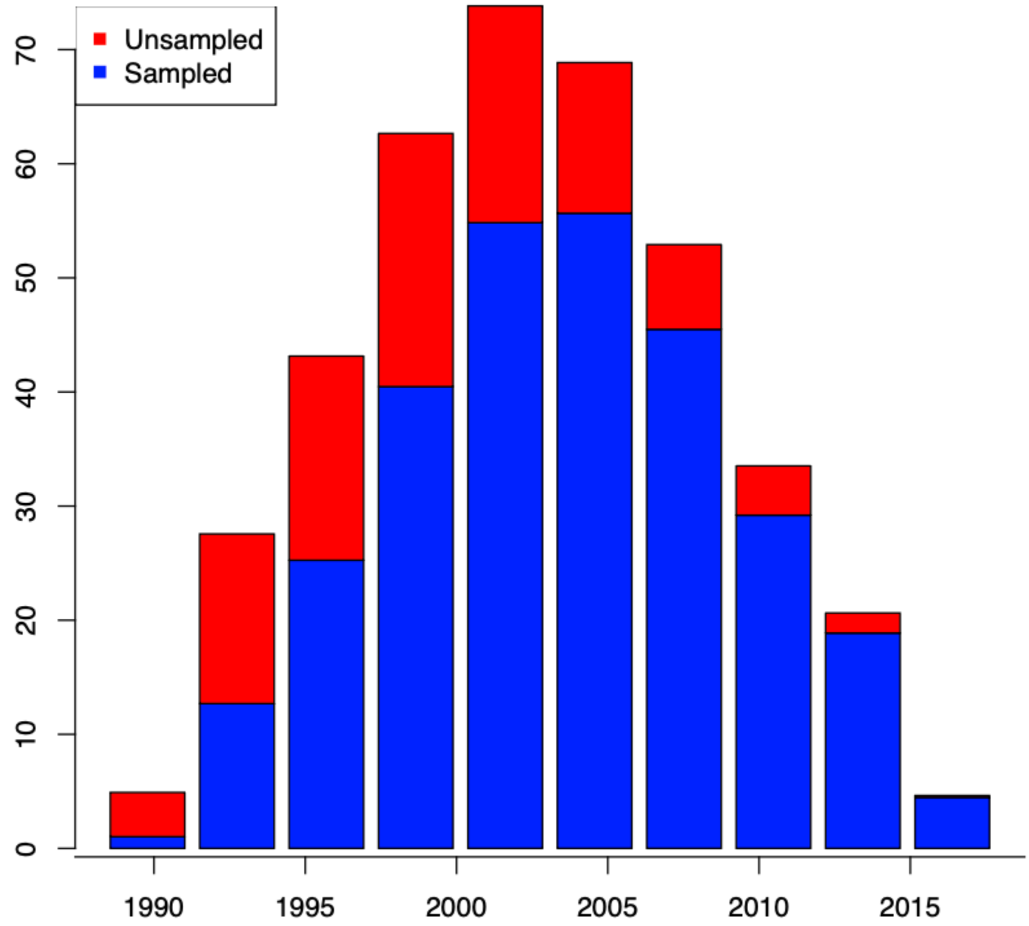

Figure S7. Histogram of sampled (n=288) and unsampled TB cases by year based on the combined posterior transmission tree for the largest cluster. TransPhylo was used to reconstruct transmission trees based on the dated phylogenies. The number of unsampled individuals was estimated as part of the model's posterior distribution, using default priors for sampling and generation intervals. The inferred number of unsampled cases represents those individuals necessary to explain the observed transmission patterns among sampled cases.

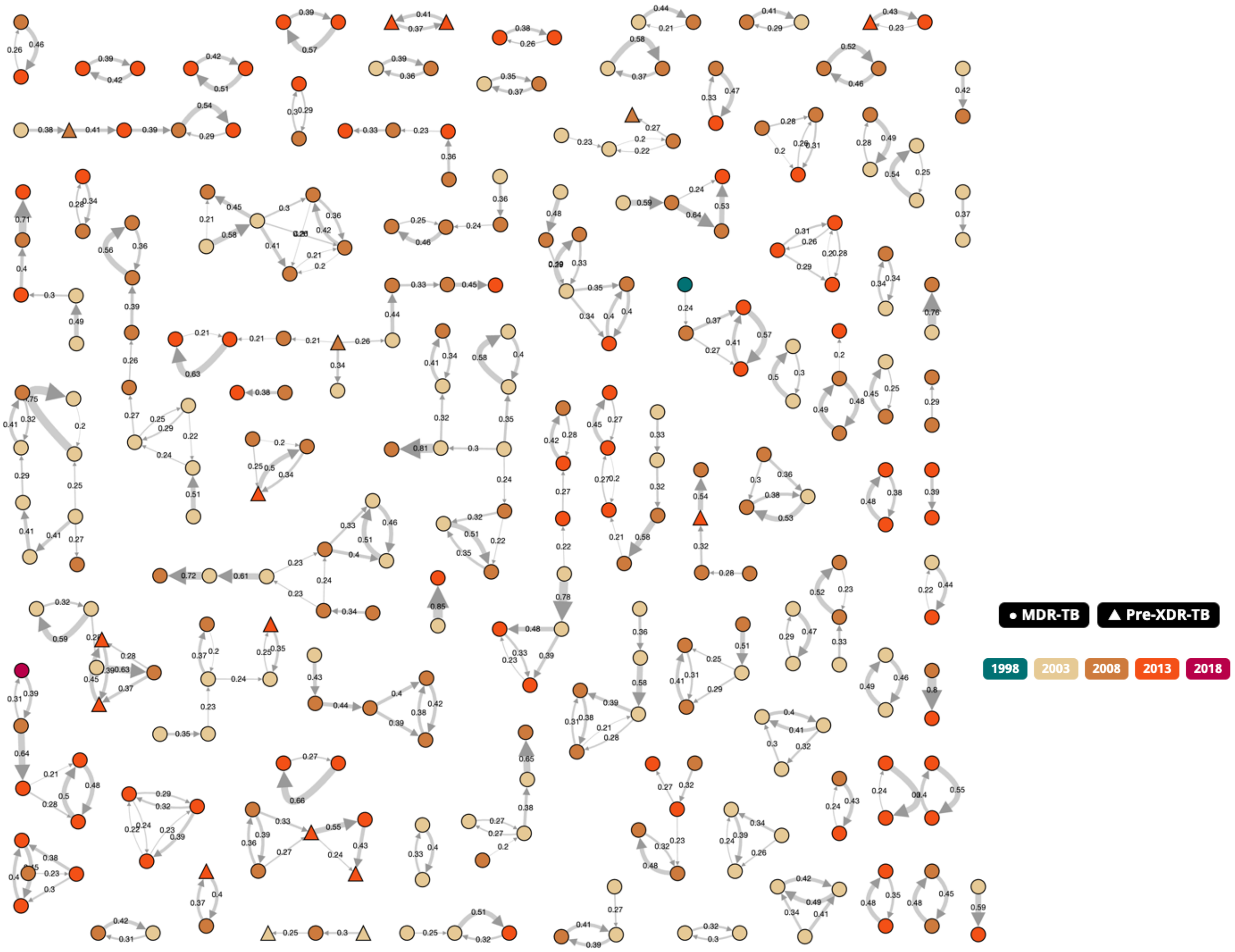

Figure S8. The transmission cluster graph depicts relationships between Mtb cases with a transmission probability greater than 0.2. Arrows indicate the direction of transmission, with arrow size reflecting the likelihood of one host infecting another. Node colors correspond to the year of sample collection: 1998 (1998-2002), 2003 (2003-2007), 2008 (2008-2012), 2013 (2013-2017), and 2018 (2018-2019).

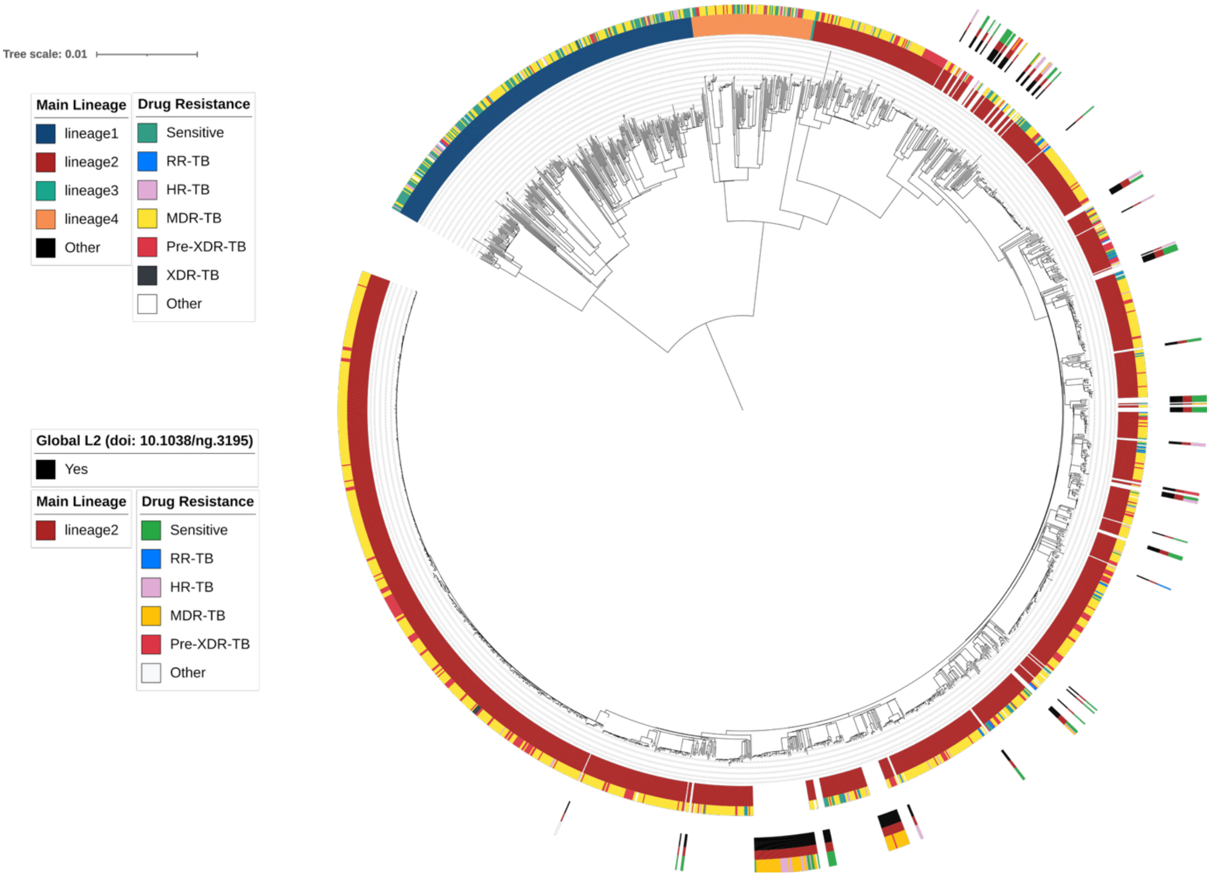

Figure S9. Phylogenetic Analysis of Thai *Mycobacterium tuberculosis* Isolates and Global Lineage 2 Strains. This circular maximum likelihood phylogenetic tree illustrates the relationships among Thai M. tuberculosis isolates (n=2,005) and a global reference set of 154 representative Lineage 2 (Beijing) strains from a previous study [DOI: 10.1038/ng.3195].

Table S2. List of drug resistance mutations

Drugs	Gene name	Changes (Count)
AMK	*eis*	c.-14C>T (8)
	rrs	n.1401A>G (99), n.1484G>T (1)
BDQ	*mmpR5*	Ser68Gly (1), Leu117Arg (5), Arg156* (2), c.198dupG (5), c.466dupC (1)
CAP	*rrs*	n.1401A>G (99), n.1484G>T (1)
	tlyA	c.363_364dupGC (1), c.52_53dupCG (3)
CFZ	*mmpR5*	Ser68Gly (1), Leu117Arg (5), Arg156* (2), c.198dupG (5), c.466dupC (1)
Cs	*alr*	Leu113Arg (3), Met343Thr (2)
	ald	c.436_437dupGC (4), c.464delG (3)
DLM	*fbiC*	c.989_990dupGC (1), c.2565_*55delGGCCTAGCCCCGGCGACGATGCCGGGTCGCGGGATGCGGCCCGTTGAGGAGCGGGGCAATCT (1)
EMB	*embA*	c.-16C>T (15), c.-16C>G (3), c.-12C>T (47), c.-11C>A (15), Asp4Asn (1)
	embB	Phe285Leu (1), Met306Val (261), Met306Ile (233), Met306Leu (21), Met306Thr (1), Tyr319Ser (32), Asp328Tyr (32), Asp328Gly (3), Tyr334His (2), Ser347Ile (1), Asp354Ala (20), Val369Ala (1), Pro404Ser (2), Glu405Asp (3), Gly406Asp (320), Gly406Ala (28), Gly406Cys (18), Gly406Ser (8), Gln497Arg (78), Gln497Pro (6), Gln497Lys (6), Glu504Asp (1), Arg507Lys (1), Ala659Thr (8), Met1000Arg (1), His1002Arg (5), Asp1024Asn (31)
ETO	*ethA*	c.-152_766del (2), Met1 (Start loss) (1), His22Pro (1), c.32dupG (1), Trp45* (3), Trp69* (1), Thr88Ile (3), c.110delA (8), c.139_293del (1), c.140delT (4), c.163_506del (1), c.168_681del (1), Glu223Lys (3), Trp228* (1), Thr232Ala (3), Tyr235* (1), Cys253* (3), Gln269* (1), c.280delA (1), Trp289* (1), Arg292* (1), Lys309* (1), Ile338Ser (1), c.341delA (10), Thr342Lys (1), Gln347* (2), Gln359* (5), Gln360* (1), c.391delT (1), Leu397Arg (3), c.413_419dupGCGGCTA (1), c.439delT (1), Glu445* (5), Trp455* (1), c.456delC (4), c.481_482delAT (1), c.489dupT (3), c.489_531delTCCGCAGCACTGGCCCGAGGACCTCGACTACGACGCTAAGAAC (3), c.551dupG (1), c.594delC (1), c.608_1137del (1), c.628dupA (1), c.639_640delGT (534), c.672_673dupGC (1), c.704_707delACAC (4), c.741_742insC (3), c.752dupG (3), c.754_755dupGC (2), c.771dupA (1), c.815delT (2), c.1031delT (1), c.1034delA (3), c.1047delT (1), c.1054delG (5), c.1080_1159delAGTGGACATCACCACGACGATGGCCTACAAGGGCATGATGCTTTCCGGCATCCCCAACATGGCCTACACGGTTGGCTACA (1), c.1123delT (1), c.1203delT (1), c.1290delC (1), c.1307dupC (1), c.1341delC (2), c.1355_1356dupGT (1), c.1361delC (1), c.1392dupC (1), c.1407delG (1), c.1431dupT (1), c.24_25insT (1)
ETO	*ethR*	Phe110Leu (1)
	inhA	c.-779G>T (2), c.-777C>T (182), c.-770T>C (15), c.-770T>A (4), c.-154G>A (16), Ile21Thr (4), Ser94Ala (29), Ile194Thr (7)
INH	*ahpC*	c.-81C>T (4), c.-74G>A (1), c.-54C>T (3), c.-52C>T (6), c.-48G>A (9), Pro2Ser (1)
	inhA	c.-779G>T (2), c.-777C>T (182), c.-770T>C (15), c.-770T>A (4), c.-154G>A (16), Ile21Val (5), Ile21Thr (4), Gly40Trp (1), Ser94Ala (29), Ile194Thr (7)
	katG	c.-7239_992del (1), c.-5295_*5855del (1), c.-2345_1109del (1), Val1Leu (1), c.40_47delGGAGCCGC (1), Asp94Gly (2), Ala110Val (1), Gly121Val (1), Gln127Pro (1), Asn138Ser (2), Asn138His (1), Ser140Asn (1), c.152delA (1), Met176Ile (1), Met257Ile (3), Gly279Asp (4), Gln295Pro (1), Asp311Gly (1), Ser315Thr (1435), Ser315Asn (25), Ser315Arg (3), Ser315Ile (2), Ser315Gly (2), Thr326Met (2), Tyr337Phe (1), Gln352* (1), Leu378Pro (3), Thr380Ile (1),

Drugs	Gene name	Changes (Count)
		Ala424Gly (21), c.603_*4483del (2), Trp728Cys (1), c.1060dupA (1), c.1752dupT (1)
KAN	*eis*	c.-14C>T (8), c.-12C>T (2), c.-10G>A (17), c.-8C>A (1)
	rrs	n.1401A>G (99), n.1484G>T (1)
LFX	*gyrA*	Gly88Ala (5), Ala90Val (88), Ser91Pro (18), Asp94Gly (121), Asp94Ala (39), Asp94Asn (20), Asp94His (11), Asp94Tyr (11), Asp94Val (3)
	gyrB	Arg446Cys (3), Arg446His (1), Ser447Phe (1), Asp461Ala (2), Gly470Cys (2), Asp494Ala (8), Asn499Asp (1), Glu501Asp (4), Ala504Val (4)
MFX	*gyrA*	Gly88Ala (5), Ala90Val (88), Ser91Pro (18), Asp94Gly (121), Asp94Ala (39), Asp94Asn (20), Asp94His (11), Asp94Tyr (11), Asp94Val (3)
	gyrB	Arg446Cys (3), Arg446His (1), Ser447Phe (1), Asp461Ala (2), Gly470Cys (2), Asp494Ala (8), Asn499Asp (1), Glu501Asp (4), Ala504Val (4)
PAS	*folC*	Thr20Pro (1), Glu40Gly (29), Ile43Thr (20), Ile43Ser (2), Arg49Trp (10), Ser150Gly (91), Glu153Ala (11), Glu153Gly (2)
	thyA	c.-6493_*3719del (2), c.-6333_*2084del (1), c.-4473_*2082del (1), c.-4473_*2083del (1), c.-4188_*2026del (1), c.-2787_*2084del (3), c.-2608_*2083del (3), c.-2608_*2082del (1), c.-1233_*1246del (2), c.-683_*84del (1), c.-522_*3884del (1), Thr22Ala (23), Trp83* (5), Gly91Arg (1)
	thyX	c.-16C>T (77)
Pa	*fbiC*	c.989_990dupGC (1), c.2565_*55delGGCCTAGCCCCGGCGACGATGCCGGGTCGCGGGATGCGGCCCGTTGAGGAGCGGGGCAATCT (1)
PZA	*pncA*	c.2565_*55delGGCCTAGCCCCGGCGACGATGCCGGGTCGCGGGATGCGGCCCGTTGAGGAGCGGGGCAATCT (1), c.-2134_*13301del (1), c.-1908_*14025del (1), c.-1832_*2695del (1), c.-1644_*7del (1), c.-1103_*3980del (1), c.-1092_*625del (2), c.-1081_314del (1), c.-1072_*3285del (2), c.-642_*3550del (2), c.-438_*27692del (1), c.-388_*2261del (1), c.-12T>C (3), c.-11A>G (25), c.-11A>C (19), Met1 (Start loss) (12), Ala3Glu (1), Leu4Ser (3), Leu4Trp (3), Ile5Ser (2), Ile6Thr (2), Val7Gly (2), Asp8Ala (5), Val9Gly (1), Gln10Pro (12), Gln10* (2), Gln10Lys (1), Asp12Ala (6), Asp12Asn (4), Phe13Leu (1), Cys14Tyr (6), Cys14Trp (1), Cys14Gly (1), Gly17Asp (1), Ser18* (1), Leu19Arg (3), Leu19Pro (3), Val21Gly (2), Leu27Pro (8), Ala28Asp (1), Ile31Thr (290), Ile31Ser (2), Leu35Pro (2), Glu37* (1), Tyr41* (2), Val44Gly (1), Ala46Val (2), Ala46Glu (1), Thr47Pro (11), Thr47Ala (1), c.47delG (1), Lys48* (1), c.48delT (1), Asp49Gly (5), Asp49Glu (1), c.50delG (1), His51Tyr (2), His51Pro (2), His51Arg (2), Pro54Ser (3), Pro54Leu (1), His57Arg (1), Ser59Pro (1), Thr61Pro (14), Pro62Leu (4), Asp63Gly (2), Asp63Ala (1), Ser66Pro (1), Ser67Pro (7), Trp68Arg (7), Trp68Cys (4), Trp68* (3), Trp68Gly (1), Pro69Arg (2), His71Arg (4), His71Asp (2), His71Tyr (2), Cys72Tyr (3), Cys72Arg (2), Thr76Pro (8), Thr76Ile (4), c.80_81insTT (1), c.80_89delTGGCCCGCGC (1), His82Asp (1), Ile90Ser (31), Ile90Asn (1), Phe94Leu (8), Phe94Ser (2), Lys96Glu (6), Lys96Thr (5), Lys96Arg (4), Lys96Asn (1), Gly97Ser (3), Gly97Asp (2), Gly97Cys (2), Gly97Arg (1), Thr100Pro (18), Tyr103* (4), Tyr103His (4), Ser104Arg (9), Gly105Val (6), Gly105Asp (3), Phe106Leu (2), Phe106Ser (1), Gly108Arg (8), Trp119Arg (3), Trp119Cys (1), Trp119Ser (1), Trp119Gly (1), Glu127* (4), Val128Gly (1), Val131Gly (1), Gly132Ser (1), Gly132Ala (1), Ile133Thr (2), Ala134Val (1), Thr135Pro (6), Asp136Gly (3), His137Pro (1), Cys138Tyr (2), Cys138Ser (1), Cys138Arg (1), Val139Gly (13), Val139Ala (5), c.139delA (3), Val139Leu (1), Gln141Pro (13), Gln141* (2), Thr142Ala (5), Thr142Lys (2), Thr142Pro (1), Thr142Met (1), Ala143Pro (1), Ala146Val (2), Leu151Ser (5), Leu151* (1),

Drugs	Gene name	Changes (Count)
		c.152_161delACATCGACCC (1), Arg154Gly (2), c.154dupA (1), Val155Gly (2), Leu156Pro (1), Leu159Pro (1), Leu159Arg (1), Thr160Pro (5), Val163Ala (7), Ser164Pro (1), c.166_167dupGA (1), c.171_172insA (1), Leu172Pro (2), Leu172Arg (2), Glu174* (2), Met175Thr (1), Val180Ala (1), Glu181* (2), Leu182Ser (1), c.220_229delAGCGGTACTC (1), c.237_240dupGGAC (1), c.244dupC (2), c.249delC (4), c.287dupA (1), c.294dupC (2), c.295delT (1), c.296delA (1), c.321dupA (1), c.354delTinsGA (1), c.364_365dupCA (1), c.377_389delATGAGGTCGATGT (1), c.386_394delATGTGGTCG (4), c.390_391dupGG (5), c.391dupG (10), c.393dupC (1), c.407dupA (5), c.407_408insC (2), c.416_417delTG (2), c.429delC (1), c.446delA (2), c.449dupG (6), c.459delC (4), c.470_471insA (1), c.486_487insA (1), c.493_496dupGCCG (1), c.519_520dupGG (2), c.559T>C (1)
RIF	*rpoB*	Val170Phe (19), Ala286Val (3), Thr400Ala (10), Phe424Leu (1), Gln429His (1), Leu430Pro (6), Leu430Arg (3), Leu430Val (1), Gln432Pro (10), Gln432Lys (7), Gln432Glu (5), Gln432Leu (1), Met434Val (2), Asp435Val (115), Asp435Tyr (26), Asp435Phe (11), Asp435Glu (6), Asp435Ala (5), Asp435Gly (4), Asp435Asn (1), Asp435Cys (1), Asp435Ile (1), Asp435Leu (1), Asp435Ser (1), Asn437His (4), Asn437Asp (2), Pro439Ser (1), Ser441Leu (21), Ser441Gln (2), Ser441Met (1), Leu443Trp (1), Thr444Pro (1), His445Tyr (209), His445Asp (107), His445Arg (56), His445Leu (33), His445Pro (6), His445Gln (5), His445Gly (4), His445Cys (4), His445Asn (3), His445Thr (1), His445Val (1), His445Ser (1), Lys446Gln (1), Arg448Gln (2), Ser450Leu (924), Ser450Trp (18), Ser450Phe (4), Ser450Tyr (3), Ser450Gln (1), Ser450Cys (1), Ala451Gly (1), Leu452Pro (71), Pro454His (2), Glu460Gly (7), Ile491Phe (13), Arg552Cys (3), Glu761Asp (2), c.1295_1300delAATTCA (2), c.1296_1304delATTCATGGA (5), c.1297_1299dupTTC (7), c.1297_1305dupTTCATGGAC (3), c.1299_1305delCATGGACinsT (2), c.1300_1305delATGGAC (5), c.1301_1303dupTGG (1), c.1303_1305delGAC (3), c.1303_1308delGACCAG (1), c.1306_1311delCAGAAC (1), c.1312_1314delAAC (17), c.1335_1337delCAA (1)
	rpoC	Gly332Arg (8), Phe452Ser (1), Ile491Thr (10), Leu527Val (8)
STM	*gid*	c.-834_*101del (1), c.-559_171del (1), Tyr22* (2), Leu26* (1), Gly28* (1), c.31_248del (7), c.52_59dupCTTGCTCG (1), Ser70Asn (1), Gly73Ala (22), Pro75Arg (1), Leu79Ser (7), c.87delC (2), c.87dupC (1), Glu92* (1), c.102delG (30), c.102dupG (1), c.115delC (9), Gln125* (1), Gln127* (1), Glu173* (3), Arg213* (1), c.254delA (2), c.294delC (1), c.311dupT (1), c.340_343delATCG (2), c.346dupC (1), c.351delG (18), c.351dupG (3), c.371dupT (2), c.373delC (2), c.386delG (3), c.477delG (1), c.518dupA (1), c.541delT (1), c.570delA (3), c.575dupG (1)
	rpsL	Lys43Arg (931), Lys88Arg (74), Lys88Met (3), Lys88Thr (2), Lys88Gln (1)
	rrs	n.513C>T (2), n.514A>C (34), n.514A>T (3), n.517C>T (21), n.878G>A (3), n.888G>A (1), n.905C>A (6), n.905C>G (1), n.906A>G (6), n.907A>C (5), n.907A>T (2)

AMK Amikacin; BDQ Bedaquiline; CAP Capreomycin; CFZ Clofazimine; Cs Cycloserine; DLM Delamanid; EMB Ethambutol; ETO Ethionamide; INH Isoniazid; KAN Kanamycin; LFX Levofloxacin; MFX Moxifloxacin; PAS Para-aminosalicylic acid; PZA Pyrazinamide; RIF Rifampicin; STM Streptomycin

Table S3. Phenotypic data (laboratory DSTs)

Characteristics		Phenotypic			Genotypic (N=2,005)	
		Max. N=1813	Number	%	Number	%
Drug resistance	Sensitive	-	59	2.94	233	11.62
	RR TB	-	4	0.20	39	1.95
	HR TB	-	19	0.95	53	2.64
	MDR TB	-	1522	75.91	1349	67.28
	Pre-XDR TB	-	201	10.02	293	14.61
	XDR TB	-	3	0.16	5	0.25
	Other	-	11	0.55	33	1.65
	Not Done	-	186	9.28	-	-
Anti-TB drug* (Resistance)	INH	1811	1732	95.64	1686	84.09
	RIF	1813	1720	94.87	1693	84.44
	EMB	1653	727	43.98	1095	54.61
	STM	1801	1113	61.8	1212	60.45
	PZA	ND	-	-	812	40.50
	LFX	1602	199	12.42	305	15.21
	MFX	1101	95	8.63	305	15.21
	AMK	1172	81	6.91	108	5.39
	KAN	1687	127	7.53	128	6.38
	ETO	ND	-	-	875	43.64
	BDQ	ND	-	-	14	0.70
	LZD	1097	4	0.36	0	0

RR-TB, Rifampicin-Resistant; HR-TB, isoniazid-monoresistant; MDR-TB, Multidrug-Resistant; Pre-XDR TB, Pre-Extensively Drug-Resistant; XDR-TB, Extensively Drug-Resistant; INH, Isoniazid; RIF, Rifampicin; EMB, Ethambutol; STM, Streptomycin; PZA, Pyrazinamide; LFX, Levofloxacin; MFX, Moxifloxacin; AMK, Amikacin, KAN, Kanamycin; ETO, Ethionamide; PAS, Para-aminosalicylic acid; CAP, Capreomycin; * The number of isolates that underwent phenotypic DST varied for each drug.

Table S4. Performance Comparison of laboratory phenotypic DST and WGS-based genotypic resistance

Drug	Phenotypic Resistance N (%)		Phenotypic Sensitive N (%)		ND	Concordance rate (%)	Sens (%)	Spec (%)	PPV (%)	NPV (%)
	Geno R	Geno S	Geno R	Geno S						
INH	1,665 (83.04)	67 (3.34)	4 (0.20)	75 (3.74)	194 (9.68)	96.08	96.13	94.94	99.76	52.82
RIF	1,668 (83.19)	52 (2.59)	5 (0.25)	88 (4.39)	192 (9.58)	96.86	96.98	94.62	99.70	62.86
STM	1,056 (52.67)	57 (2.84)	128 (6.38)	560 (27.93)	204 (10.17)	89.73	94.88	81.4	89.19	90.76
EMB	627 (31.27)	100 (4.99)	367 (18.30)	559 (27.88)	352 (17.56)	71.75	86.24	60.37	63.08	84.83
AMK	59 (2.94)	22 (1.10)	7 (0.35)	1,084 (54.06)	833 (41.55)	97.53	72.84	99.36	89.39	98.01
KAN	100 (4.99)	27 (1.35)	27 (1.35)	1,533 (76.46)	318 (15.86)	96.80	78.74	98.27	78.74	98.27
LFX	178 (8.88)	21 (1.05)	109 (5.44)	1,294 (64.54)	403 (20.10)	91.89	89.45	92.23	62.02	98.4
MFX	87 (4.34)	8 (0.40)	117 (5.84)	889 (44.34)	904 (45.09)	88.65	91.58	88.37	42.65	99.11

Geno Genotypic; R Resistance; S Sensitive; ND not determined; Spec specificity; Sens sensitivity; PPV Positive Predictive Value; NPV Negative Predictive Value; INH Isoniazid; RIF Rifampicin; EMB Ethambutol; STM Streptomycin; LFX Levofloxacin; MFX Moxifloxacin; AMK Amikacin, KAN Kanamycin

Table S5. High-confidence mutations predicted to be related to resistance to TB drugs.

Drug	Gene name	Change	Our study (N=2,005)				Global (N = 50,723) [*]			
			Phenotypic DST		DST group		Phenotypic DST		DST group	
			R	S	MDR	Sen	R	S	MDR	Sen
INH	*katG*	Pro232Ser	3				8		5	
	katG	Gly285Ala	2	1				1		
	katG	Gly121Ala	2		1		1	1		1
	katG	Leu707Phe	2	1				1		
	katG	Trp300Arg	1				2			1
	katG	Trp341Gly	2		1					
	katG	Phe483Ser	1							
	katG	Leu430Pro	1							
	katG	Leu333Pro	1			1	2			
	katG	Trp412Ser	1							
	katG	Arg385Pro	1				2		3	
	katG	Asp142Gly	1				10		1	4
	katG	Ala550Thr	1			1				
	katG	Ala245Glu	1							
	katG	c.-9A>T	1							
	katG	c.-7G>C	1							
	katG	Tyr64Cys	1				1		1	
	katG	Gln679Pro	1							
	katG	Leu662Pro	1		1					
	katG	Leu427Pro	1		1					1
	katG	Gly699Glu	1		1		5			
	katG	c.-1286_444del	2		2					
	ahpC	c.-72C>T	1				36	3	23	2
	ahpC	c.-51G>A	2		1		51	8	19	7
RIF	*rpoB*	c.1290_1298delGAGCCAATT	1							
	rpoB	c.1292_1300delGCCAATTCA	2			1				
	rpoB	Val496Met	1		1		16		9	
	rpoB	Lys37Arg	34		33		9	2	9	1
	rpoB	Met655Thr	11	0	10	1	1	49	1	47
	rpoC	Gly1198Ser	93	1	65	2	21	18	13	15
	rpoC	Lys445Arg	14		13		54		37	
EMB	*embA*	c.-43G>C	4	2	6		90	23	97	
	embA	Leu832Phe	2		2			1		
	embA	c.-16C>A	13	5	17		48	15	58	1
	embA	c.-15C>G	3		1		19	12	20	
	embA	Arg537Trp			1					
	embA	Gly814Glu	3	1	4				1	
	embA	Val613Gly	1		1					
	embB	Gln497His	7	1	8		17	5	18	
	embB	Ile303Leu	1	1	2		1		1	
	embB	Gln445Arg	1				54		23	
	embB	Asn399Thr	1		1		3	2	5	
	embB	Asn318Asp	1		1		1	1	3	
	embB	Asn296Tyr	1	1	2		2		1	

Drug	Gene name	Change	Our study (N=2,005)				Global (N = 50,723) *			
			Phenotypic DST		DST group		Phenotypic DST		DST group	
			R	S	MDR	Sen	R	S	MDR	Sen
	embB	Ala81Thr	1							
	embB	Gly443Cys	2		2					
	embC	Ala177Thr	1		1					
STM	*gid*	Val112Gly	1		1			1	3	1
	gid	Ala138Val	1	1	1		12	8	26	28
	gid	Arg21Pro	1	3	4		2	1	7	2
	gid	Leu91Arg	1				1		4	5
	gid	Leu90Pro	1	1	1			1	1	
	gid	Leu16Pro	1	1	2		1		3	2
	gid	Ile81Thr	1	1	2		1	1	4	9
	gid	Gly157Glu	1		1		1		6	1
	gid	Cys52Trp	1		1			2		2
	gid	Cys52Phe	1	1	2		3		3	2
	gid	Arg64Trp	1			1	1			2
	rpsL	Lys43Asn	4							
	rpsL	Arg86Gln	3		3				4	2
	rrs	n.16T>C	4		4		1		2	
	rrs	n.1401A>G	73	18	23		541	126	790	
	rrs	n.13A>G	3				2			
	rrs	n.23T>G	2	2	1				2	
	rrs	n.908A>C	2		2		11	1	17	1
	rrs	n.908A>T	1				1		1	

R Resistance; S Sensitive; MDR multidrug resistant; INH Isoniazid; RIF Rifampicin; EMB Ethambutol; STM Streptomycin; * The global and Thailand percentage of anti-TB drug resistance mutations identified by TB-Profiler database

Table S6. Discrepancies between laboratory phenotypic DSTs and WGS-based genotypic resistance profiles

		Phenotypic DST categories								
		Sens	RR- TB	HR- TB	MDR-TB	Pre-XDR-TB	XDR-TB	Other	ND	Total
Genotypic resistance	Sensitive	51	0	2	18	4	0	4	154	233
	RR -TB	1	5	0	30	1	0	1	1	39
	HR-TB	2	0	17	21	3	0	0	10	53
	MDR-TB	0	0	0	1331	9	3	1	5	1349
	Pre-XDR-TB	1	0	0	111	178	0	2	1	293
	XDR-TB	0	0	0	3	2	0	0	0	5
	Other	4	0	0	6	1	0	7	15	33
	Total	59	5	19	1520	198	3	15	186	2005

ND not determined; Sens sensitive; RR-TB Rifampicin-Resistant; HR-TB isoniazid-monoresistant; MDR-TB Multidrug-Resistant; Pre-XDR-TB Pre-Extensively Drug-Resistant; XDR-TB Extensively Drug-Resistant

Table S7. Anti-TB Drug Resistance Rates and Gene Changes (Resistance Rate < 50%; Total > 10 Mtb isolates)

Drug	Gene name	Changes	Phenotypic DSTs (N)		Total (N)	Resistance rate (%)
			R	S		
EMB	*embB*	Gly406Asp	128	156	284	45
KAN	*eis*	c.-10G>A	0	17	17	0
LFX	*gyrA*	Asp94Ala	15	20	35	43
	gyrA	Ser91Pro	8	10	18	44
MFX	*gyrA*	Ala90Val	14	40	54	26
	gyrA	Asp94Ala	4	22	26	15
	gyrA	Ser91Pro	5	10	15	33
STM	*gid*	c.102delG	9	12	21	43
	gid	c.351delG	4	11	15	27

EMB, Ethambutol; STM, Streptomycin; LFX, Levofloxacin; MFX, Moxifloxacin; AMK, Amikacin, KAN, Kanamycin

Table S8. Summary of Percentages of Lineage, genotypic drug resistance (DR) categories, and Region for Clusters with ≥10 Isolates (SNP Cut-off of 13)

Cluster No.		1	2	3	4	5	6	7	8	9	10	11	12	13	14	15	16
No. of isolates		288	81	64	48	43	42	36	22	19	18	16	12	11	11	10	10
Region (%)	Central	96.9	75.3	85.9	18.8	44.2	61.9	38.9	100	57.9	83.3	50	83.3	63.6	9.1	90	50
	Northeast	1.4	17.3	4.7	18.8	44.2	23.8	41.7	-	36.8	11.1	37.5	8.3	9.1	18.2	-	20
	Northern	1	4.9	4.7	18.8	11.6	4.8	11.1	-	-	5.6	12.5	8.3	9.1	-	10	-
	Southern	0.7	2.5	4.7	43.8	-	9.5	8.3	-	5.3	-	-	-	18.2	72.7	-	30
Sub-lineage		2.2.1	2.2.1	2.1	2.1.1	2.2.1	2.2.1	2.2.1	2.2.1	2.2.1	2.2.1	2.2.1	4.2.2	2.2.1	2.2.1.1	2.2.1.1	2.2.1
Drug Res. (%)	HR-TB	-	-	-	4.2	-	-	2.8	-	-	-	-	-	-	-	-	-
	MDR-TB	93.8	82.7	68.8	93.8	81.4	95.2	69.4	-	-	100	87.5	75	100	72.7	100	100
	Pre-XDR	6.3	17.3	28.1	2.1	18.6	4.8	16.7	100	100	-	12.5	25	-	27.3	-	-
	RR-TB	-	-	3.1	-	-	-	-	-	-	-	-	-	-	-	-	-
	XDR-TB	-	-	-	-	-	-	11.1	-	-	-	-	-	-	-	-	-
Collection Year	1999	-	-	1.6	-	-	-	-	-	-	-	-	-	-	-	-	-
	2000	-	-	1.6	-	-	-	-	-	-	-	-	-	-	-	-	-
	2001	-	1.2	0.0	2.1	-	-	-	-	-	-	-	8.3	-	-	-	10.0
	2002	0.3	1.2	1.6	8.3	-	-	-	-	-	5.6	-	8.3	-	-	-	0.0
	2003	2.8	3.7	7.8	0.0	-	4.8	2.8	-	-	0.0	6.3	0.0	-	-	-	0.0
	2004	5.6	1.2	3.1	0.0	-	0.0	0.0	-	10.5	0.0	0.0	8.3	-	-	-	0.0
	2005	6.6	4.9	7.8	6.3	-	0.0	2.8	9.1	0.0	5.6	0.0	0.0	9.1	-	-	0.0
	2006	7.3	6.2	4.7	6.3	7.0	0.0	2.8	4.5	5.3	5.6	0.0	8.3	0.0	-	-	0.0
	2007	12.8	9.9	3.1	6.3	2.3	0.0	8.3	13.6	0.0	0.0	6.3	8.3	0.0	-	-	10.0
	2008	6.6	7.4	9.4	8.3	2.3	0.0	11.1	22.7	0.0	0.0	12.5	8.3	9.1	-	10.0	0.0
	2009	11.1	3.7	7.8	4.2	0.0	9.5	5.6	9.1	10.5	0.0	12.5	8.3	18.2	-	0.0	0.0
	2010	6.3	2.5	9.4	8.3	0.0	9.5	2.8	0.0	0.0	11.1	0.0	0.0	0.0	-	10.0	0.0
	2011	8.3	3.7	6.3	2.1	4.7	0.0	16.7	4.5	0.0	5.6	6.3	8.3	0.0	-	10.0	0.0
	2012	3.1	7.4	4.7	2.1	4.7	14.3	8.3	22.7	5.3	11.1	12.5	0.0	9.1	27.3	10.0	20.0
	2013	3.1	4.9	1.6	0.0	4.7	0.0	2.8	0.0	5.3	11.1	6.3	0.0	18.2	9.1	0.0	10.0
	2014	8.7	8.6	7.8	10.4	27.9	11.9	11.1	4.5	26.3	11.1	12.5	0.0	27.3	18.2	10.0	30.0
	2015	5.2	17.3	10.9	14.6	18.6	14.3	2.8	0.0	10.5	11.1	6.3	8.3	0.0	18.2	30.0	10.0
	2016	3.1	2.5	4.7	12.5	14.0	14.3	11.1	4.5	15.8	11.1	0.0	16.7	0.0	9.1	0.0	0.0
	2017	8.7	13.6	6.3	6.3	14.0	21.4	11.1	4.5	10.5	11.1	18.8	8.3	9.1	18.2	20.0	10.0
	2019	0.3	-	-	0.0	-	-	-	-	-	-	-	-	-	-	-	-
	2020	-	-	-	2.1	-	-	-	-	-	-	-	-	-	-	-	-

- represents a zero count; RR-TB Rifampicin-Resistant; HR-TB isoniazid-monoresistant; MDR-TB Multidrug-Resistant; Pre-XDR Pre-Extensively Drug-Resistant; XDR-TB Extensively Drug-Resistant; L Lineage

Table S9. Logistic Regression Analysis of Factors Associated with Clustered isolates

Variable	OR	LCL	UCL	P-value
Lineage L4 vs. L1	2.03	1.23	3.36	*
Lineage L2 vs. L1	7.41	5.34	10.29	***
HR-TB vs. Sensitive	2.96	1.47	5.98	*
MDR-TB vs. Sensitive	5.82	3.93	8.61	***
Pre-XDR-TB vs. Sensitive	10.98	6.72	17.94	***
XDR-TB vs. Sensitive	11.30	1.00	126.89	*
Central vs. Northern region	2.94	2.02	4.29	***
Northeastern vs. Northern region	2.76	1.80	4.23	***

* P < 0.05; ***, P < 0.001; L Lineage; OR odds ratio; LCL 95% lower confidence limit; UCL 95% upper confidence limit; RR-TB rifampicin resistant; HR-TB, isoniazid-monoresistant; MDR-TB Multidrug-Resistant; Pre-XDR-TB Pre-Extensively Drug-Resistant; XDR-TB Extensively Drug-Resistant

Table S10. SNPs Associated with Clustered isolates (GEMMA, beta P-value < 0.001)

Gene	Ref	Alt	Position	Protein Change	beta	OR	LCL	UCL	P	Gene function
katG (Rv1908c)	C	G	2155168	Ser315Thr	0.09	7.94	6.39	9.87	***	a catalase-peroxidase, converts INH to an active form
folC (Rv2447c)	T	C	2747151	Ser150Gly	0.19	6.50	3.13	13.51	***	folate biosynthesis, involved in the activation of Para-aminosalicylic acid (PAS)
ppe8 (Rv0355c)	C	T	427755	Val2309Ile	0.22	2.81	1.05	8.28	*	adaptation to host defence mechanisms
folK (Rv3606c)	C	G	4048607	Gln47His	-0.30	0.65	0.43	0.99	*	dihydrofolate biosynthesis pathway

* P < 0.05; *** P < 0.00001; OR odds ratio; LCL 95% lower confidence limit; UCL 95% upper confidence limit

Table S11. Association between drug resistance, strain types, and Mtb genotype clusters (≤5 and 13 SNPs difference).

Variable	Cut-off 5		OR (95% CI)	Cut-off 13		OR (95% CI)
	**Cluster n=888**	**Non-Cluster n=1117**		**Cluster n=1265**	**Non-Cluster n=740**	
DR						
Sensitive	22 (9.44)	211 (90.56)	0.11 (0.07-0.17)*	43 (18.45)	190 (81.55)	0.10 (0.07-0.14)*
RR-TB	6 (15.38)	33 (84.62)	0.22 (0.09-0.54)*	14 (35.90)	25 (64.10)	0.32 (0.17-0.62)*
HR-TB	13 (24.53)	40 (75.47)	0.40 (0.21-0.75)	22 (41.51)	31 (58.49)	0.40 (0.23-0.70)*
**MDR-TB**	**664 (49.22)**	**685 (50.78)**	**1.87 (1.54-2.27)***	**936 (69.38)**	**413 (30.62)**	**2.25 (1.86-2.73)***
**Pre-XDR**	**175 (59.73)**	**118 (40.27)**	**2.08 (1.61-2.67)***	**242 (82.59)**	**51 (17.41)**	**2.78 (2.33-4.39)***
XDR-TB	4 (80.00)	1 (20.00)	5.05 (0.56-45.26)	4 (80.00)	1 (20.00)	2.34 (0.26-21.01)
Other	4 (12.12)	29 (87.88)	0.17 (0.06-0.48)*	4 (12.12)	29 (87.88)	0.08 (0.03-0.22)*
Main lineage						
L1	41 (12.65)	283 (87.35)	0.14 (0.10-0.20)*	62 (19.14)	262 (80.86)	0.09 (0.07-0.13)*
**L2**	**823 (52.45)**	**746 (47.55)**	**6.30 (4.75-8.34)***	**1158 (73.80)**	**411 (26.20)**	**8.66 (6.78-11.07)***
L4	24 (21.82)	86 (78.18)	0.33 (0.21-0.53)*	45 (40.91)	65 (59.09)	0.38 (0.26-0.57)*
Comparison lineage						
**L2 vs. L1**	-	-	**7.61 (5.41-10.72)***	-	-	**11.91 (8.83-16.05)***
**L2 vs. L4**	-	-	**3.95 (2.49-6.28)***	-	-	**4.07 (2.74-6.05)***
**L4 vs. L1**	-	-	**1.93 (1.10-3.37)***	-	-	**2.93 (1.83-4.68)***

*p-value < 0.001; OR odds ratio; L Lineage; Non-Cluster = non-clustered isolates; CI confidence interval
