# Supplementary material for: Genomic decoding of drug-resistant tuberculosis transmission in Thailand over three decades
Source: Sci Rep. 2025 Aug 13;15:29617. doi: 10.1038/s41598-025-15093-7 (PMC12344131; doi:10.1038/s41598-025-15093-7)
Supplement: Supplementary file 2 — Supplementary Information 2. [file 41598_2025_15093_MOESM2_ESM.pdf]

Table S1: Isolate data

No	Province	Region	Year	Lineage	DR	ENA accession	Alternative
1	Kanchanaburi	Central	2006	2.2.1	MDR-TB	ERR13666314	SRR5184980
2	Kanchanaburi	Central	2007	2.2.1	MDR-TB	ERR13666425	SRR5184978
3	Kanchanaburi	Central	2007	2.2.1	MDR-TB	ERR13665985	SRR5184977
4	Kanchanaburi	Central	2007	2.2.1	MDR-TB	ERR13665968	SRR5184976
5	Kanchanaburi	Central	2007	2.2.1	MDR-TB	ERR13666401	SRR5184975
6	Kanchanaburi	Central	2008	2.2.1	MDR-TB	ERR13666532	
7	Kanchanaburi	Central	2009	2.2.1	XDR-TB	ERR13666874	
8	Phitsanulok	Northern	2009	2.2.1	Pre-XDR-TB	ERR13666738	SRR5709931
9	Kanchanaburi	Central	2003	2.2.1	MDR-TB	ERR13666161	SRR1564305
10	Kanchanaburi	Central	2003	2.2.1	XDR-TB	ERR13666538	SRR5184989
11	Kanchanaburi	Central	2003	2.2.1	MDR-TB	ERR13666234	SRR5184988
12	Kanchanaburi	Central	2003	2.2.1	MDR-TB	ERR13666048	SRR5184987
13	Kanchanaburi	Central	2004	2.2.1	MDR-TB	ERR13666558	SRR5184986
14	Kanchanaburi	Central	2004	2.2.1	MDR-TB	ERR13666071	SRR5184985
15	Kanchanaburi	Central	2005	2.2.1	MDR-TB	ERR13666336	SRR5184983
16	Kanchanaburi	Central	2005	2.2.1	MDR-TB	ERR13666801	SRR5184982
17	Kanchanaburi	Central	2005	2.2.1	MDR-TB	ERR13666490	SRR5184981
18	Kanchanaburi	Central	2017	2.1	Pre-XDR-TB	ERR13666674	
19	Kanchanaburi	Central	2003	2.2.1	MDR-TB	ERR13666243	
20	Kanchanaburi	Central	2006	2.2.1	MDR-TB	ERR13666259	
21	Kanchanaburi	Central	2007	2.2.1	XDR-TB	ERR13666813	
22	Kanchanaburi	Central	2007	2.2.1	MDR-TB	ERR13666058	
23	Kanchanaburi	Central	2007	2.2.1	MDR-TB	ERR13666271	
24	Kanchanaburi	Central	2007	2.2.1	MDR-TB	ERR13666793	
25	Kanchanaburi	Central	2007	2.2.1	MDR-TB	ERR13666734	
26	Kanchanaburi	Central	2007	2.2.1	MDR-TB	ERR13666326	
27	Kanchanaburi	Central	2007	2.2.1	MDR-TB	ERR13666762	
28	Nong Khai	Northeastern	2004	2.2.1	MDR-TB	ERR13666200	
29	Kanchanaburi	Central	2007	2.2.1	MDR-TB	ERR13666666	
30	Kanchanaburi	Central	2008	2.2.1	MDR-TB	ERR13666588	
31	Kanchanaburi	Central	2008	2.2.1	MDR-TB	ERR13666770	
32	Kanchanaburi	Central	2008	2.2.1	MDR-TB	ERR13665965	
33	Kanchanaburi	Central	2011	2.2.1	MDR-TB	ERR13665992	
34	Kanchanaburi	Central	2008	2.1	Pre-XDR-TB	ERR13666845	SRR11715720
35	Kanchanaburi	Central	2009	2.2.1	MDR-TB	ERR13666361	
36	Kanchanaburi	Central	2009	2.2.1	MDR-TB	ERR13666830	
37	Kanchanaburi	Central	2004	2.2.1	MDR-TB	ERR13665900	
38	Kanchanaburi	Central	2007	2.2.1	MDR-TB	ERR13666606	
39	Kanchanaburi	Central	2008	2.2.1	MDR-TB	ERR13665981	
40	Kanchanaburi	Central	2009	2.2.1	MDR-TB	ERR13666185	
41	Kanchanaburi	Central	2009	2.2.1	MDR-TB	ERR13666399	
42	Kanchanaburi	Central	2009	2.2.1	MDR-TB	ERR13666515	
43	Kanchanaburi	Central	2005	2.2.1	MDR-TB	ERR13666752	
44	Kanchanaburi	Central	2011	2.2.1	MDR-TB	ERR13666154	
45	Kanchanaburi	Central	2004	2.2.1	Pre-XDR-TB	ERR13666309	
46	Kanchanaburi	Central	2010	2.2.1	MDR-TB	ERR13666263	
47	Kanchanaburi	Central	2005	2.2.1	MDR-TB	ERR13666720	
48	Kanchanaburi	Central	2006	2.2.1	MDR-TB	ERR13666442	
49	Kanchanaburi	Central	2007	2.2.1	MDR-TB	ERR13666809	
50	Kanchanaburi	Central	2011	2.2.1	MDR-TB	ERR13666341	

51	Kanchanaburi	Central	2011	2.2.1	MDR-TB	ERR13666484	
52	Kanchanaburi	Central	2011	2.2.1.1	MDR-TB	ERR13665982	
53	Kanchanaburi	Central	2011	2.2.1	MDR-TB	ERR13666713	
54	Kanchanaburi	Central	2012	2.2.1	MDR-TB	ERR13665871	
55	Kanchanaburi	Central	2012	2.2.1.1	MDR-TB	ERR13666700	
56	Kanchanaburi	Central	2012	2.2.1	MDR-TB	ERR13666735	
57	Kanchanaburi	Central	2012	2.2.1	MDR-TB	ERR13666545	
58	Kanchanaburi	Central	2012	2.2.1	MDR-TB	ERR13666407	
59	Kanchanaburi	Central	2012	2.2.1	MDR-TB	ERR13665970	
60	Kanchanaburi	Central	2012	2.2.1	MDR-TB	ERR13665988	
61	Kanchanaburi	Central	2012	2.2.1	MDR-TB	ERR13666410	
62	Yala	Southern	2002	2.2.1	MDR-TB	ERR13666139	
63	Bangkok	Central	2004	2.2.1	Pre-XDR-TB	ERR13666274	
64	Phatthalung	Southern	2013	1.1	RR-TB	ERR13666882	
65	Sakon Nakhon	Northeastern	2006	2.2.1	MDR-TB	ERR13666652	
66	Uthai Thani	Northern	2008	1.2.1.2.1	Sensitive	ERR13666553	
67	Nong Bua Lamphu	Northeastern	2002	2.2.1	MDR-TB	ERR13666551	
68	Kanchanaburi	Central	2007	2.2.1	MDR-TB	ERR13666708	
69	Bangkok	Central	2002	2.2.1	MDR-TB	ERR13665880	
70	Ratchaburi	Central	2011	2.2.1	MDR-TB	ERR13665923	
71	Saraburi	Central	2004	2.2.1	MDR-TB	ERR13666701	
72	Trang	Southern	2005	2.2.1	MDR-TB	ERR13666297	
73	Trang	Southern	2005	2.2.1	MDR-TB	ERR13666613	
74	Nong Khai	Northeastern	2008	1.1.1	MDR-TB	ERR13666170	
75	Buri Ram	Northeastern	2007	2.2.1	MDR-TB	ERR13666511	
76	Rayong	Central	2004	2.2.1	MDR-TB	ERR13666001	
77	Buri Ram	Northeastern	2007	2.2.1	MDR-TB	ERR13666680	
78	\akhon Ratchasima	Northeastern	2008	2.2.1	MDR-TB	ERR13666733	
79	Bangkok	Central	2009	2.2.1	MDR-TB	ERR13666398	
80	Nakhon Pathom	Central	2002	2.2.1	MDR-TB	ERR13666872	
81	Nan	Northern	2002	2.2.1	MDR-TB	ERR13666335	
82	Nan	Northern	2005	2.2.1	MDR-TB	ERR13666610	
83	Nan	Northern	2007	2.2.1	MDR-TB	ERR13666537	
84	Nan	Northern	2007	1.2.2.2	MDR-TB	ERR13666548	
85	Suphan Buri	Central	2003	2.2.1	MDR-TB	ERR13666640	
86	Nong Khai	Northeastern	2004	1.1.1	MDR-TB	ERR13666368	
87	Suphan Buri	Central	2010	2.2.1	MDR-TB	ERR13666586	
88	Nonthaburi	Central	2010	2.2.1	MDR-TB	ERR13666805	
89	Kanchanaburi	Central	2007	1.2.1.2.1	Sensitive	ERR13666568	
90	Kanchanaburi	Central	2007	1.2.1.2.1	Sensitive	ERR13666657	
91	Suphan Buri	Central	2004	2.2.1	MDR-TB	ERR13666306	
92	Si Sa Ket	Northeastern	2002	2.2.1	Pre-XDR-TB	ERR13666555	
93	Rayong	Central	2005	2.2.1	MDR-TB	ERR13666286	
94	Rayong	Central	2009	2.2.1	Pre-XDR-TB	ERR13666015	
95	Rayong	Central	2006	2.1	MDR-TB	ERR13666162	SRR11715723
96	Rayong	Central	2005	2.2.1	Pre-XDR-TB	ERR13666086	
97	Ratchaburi	Central	2005	2.2.1	Pre-XDR-TB	ERR13666039	
98	Lop Buri	Central	2006	2.2.1	Pre-XDR-TB	ERR13666549	
99	Sakon Nakhon	Northeastern	2006	2.2.1	Pre-XDR-TB	ERR13666534	
100	Sukhothai	Northern	2012	2.2.1	MDR-TB	ERR13666075	
101	Surat Thani	Southern	2014	2.2.1	Pre-XDR-TB	ERR13666453	
102	Nong Khai	Northeastern	2007	2.1	MDR-TB	ERR13666544	SRR11715715

103	Ratchaburi	Central	2009	2.2.1	MDR-TB	ERR13666221	
104	Ratchaburi	Central	2010	2.2.1	MDR-TB	ERR13666112	
105	Suphan Buri	Central	2006	2.2.1	Pre-XDR-TB	ERR13665939	
106	Suphan Buri	Central	2008	4.2.2	MDR-TB	ERR13666382	
107	Suphan Buri	Central	2008	2.2.1	MDR-TB	ERR13666468	
108	Kanchanaburi	Central	2003	1.1.1	Sensitive	ERR13666413	
109	Kanchanaburi	Central	2005	2.2.1	Other	ERR13666178	
110	Suphan Buri	Central	2010	2.2.1	Pre-XDR-TB	ERR13666053	
111	Kanchanaburi	Central	2008	1.1.3.1	Sensitive	ERR13666689	
112	Kanchanaburi	Central	2008	1.1.3.3	Sensitive	ERR13665931	
113	Kanchanaburi	Central	2008	1.1.3.3	Sensitive	ERR13666519	
114	Kanchanaburi	Central	2009	2.2.1	Sensitive	ERR13666319	
115	Kanchanaburi	Central	2009	1.1.3.1	Sensitive	ERR13666247	
116	Kanchanaburi	Central	2009	2.2.1	Other	ERR13666510	
117	Kanchanaburi	Central	2010	1.1.3.3	Sensitive	ERR13666469	
118	Kanchanaburi	Central	2010	2.2.1.2	Sensitive	ERR13666688	
119	Kanchanaburi	Central	2010	2.1	Sensitive	ERR13666795	SRR11715718
120	Kanchanaburi	Central	2010	4.4.2	HR-TB	ERR13666264	
121	Kanchanaburi	Central	2010	2.2.1	MDR-TB	ERR13665879	
122	Kanchanaburi	Central	2010	1.1.3.1	Sensitive	ERR13665928	
123	Kanchanaburi	Central	2011	2.1	Sensitive	ERR13666203	SRR11715717
124	Kanchanaburi	Central	2003	2.2.1	RR-TB	ERR13666560	
125	Kanchanaburi	Central	2004	2.2.1	Sensitive	ERR13666397	
126	Kanchanaburi	Central	2004	4.4.2	Sensitive	ERR13666563	
127	Kanchanaburi	Central	2007	1.1.3.1	Sensitive	ERR13666641	
128	Kanchanaburi	Central	2004	2.2.1	MDR-TB	ERR13666182	
129	akhon Si Thammara	Southern	2003	2.2.1	MDR-TB	ERR13666719	
130	Satun	Southern	2005	2.2.1	MDR-TB	ERR13666646	
131	Rayong	Central	2006	2.2.1	MDR-TB	ERR13666618	
132	Chon Buri	Central	2005	2.2.1	MDR-TB	ERR13666869	
133	Nonthaburi	Central	2005	2.2.1	MDR-TB	ERR13666504	
134	Nonthaburi	Central	2005	2.2.1	MDR-TB	ERR13666428	
135	Yala	Southern	2005	1.1.1	MDR-TB	ERR13666426	
136	Roi Et	Northeastern	2005	2.2.1	MDR-TB	ERR13666316	
137	Nonthaburi	Central	2005	2.2.1	MDR-TB	ERR13666072	
138	Kanchanaburi	Central	2003	2.2.1	MDR-TB	ERR13666333	
139	Kanchanaburi	Central	2008	2.2.1	MDR-TB	ERR13666345	
140	Kanchanaburi	Central	2003	2.2.1.1	MDR-TB	ERR13666251	
141	Kanchanaburi	Central	2005	2.2.1	MDR-TB	ERR13666121	
142	Kanchanaburi	Central	2005	2.2.1	MDR-TB	ERR13665946	
143	Kanchanaburi	Central	2006	2.2.1	MDR-TB	ERR13666224	
144	Kanchanaburi	Central	2008	2.2.1	MDR-TB	ERR13666257	
145	Kanchanaburi	Central	2009	2.2.1	MDR-TB	ERR13666886	
146	Kanchanaburi	Central	2009	2.2.1	MDR-TB	ERR13666447	
147	Kanchanaburi	Central	2009	2.2.1	MDR-TB	ERR13666832	
148	Kanchanaburi	Central	2009	2.2.1	MDR-TB	ERR13666258	
149	Kanchanaburi	Central	2010	2.2.1	MDR-TB	ERR13666492	
150	Kanchanaburi	Central	2010	2.2.1	MDR-TB	ERR13666761	
151	Kanchanaburi	Central	2011	2.2.1	MDR-TB	ERR13666207	
152	Kanchanaburi	Central	2005	2.2.1	MDR-TB	ERR13666857	
153	Kanchanaburi	Central	2006	2.2.1	MDR-TB	ERR13666044	
154	Kanchanaburi	Central	2009	2.2.1	MDR-TB	ERR13666372	

155	Kanchanaburi	Central	2005	2.2.1	MDR-TB	ERR13666006	
156	Kanchanaburi	Central	2005	2.2.1	MDR-TB	ERR13666566	
157	Kanchanaburi	Central	2009	2.2.1	MDR-TB	ERR13666788	
158	Kanchanaburi	Central	2009	2.2.1	MDR-TB	ERR13666348	
159	Kanchanaburi	Central	2004	2.2.1	MDR-TB	ERR13666038	
160	Kanchanaburi	Central	2004	2.2.1	MDR-TB	ERR13666429	
161	Kanchanaburi	Central	2005	2.1	Pre-XDR-TB	ERR13666059	SRR11715724
162	Kanchanaburi	Central	2005	2.2.1	MDR-TB	ERR13666461	
163	Kanchanaburi	Central	2005	2.2.1	MDR-TB	ERR13665893	
164	Kanchanaburi	Central	2005	2.2.1	MDR-TB	ERR13666064	
165	Kanchanaburi	Central	2006	2.2.1	MDR-TB	ERR13665926	
166	Kanchanaburi	Central	2006	2.2.1	MDR-TB	ERR13666132	
167	Kanchanaburi	Central	2006	2.2.1	MDR-TB	ERR13666747	
168	Kanchanaburi	Central	2006	2.2.1	MDR-TB	ERR13666362	
169	Kanchanaburi	Central	2007	2.2.1	MDR-TB	ERR13666197	
170	Kanchanaburi	Central	2007	2.2.1	MDR-TB	ERR13666087	
171	Kanchanaburi	Central	2007	2.2.1	Other	ERR13665901	
172	Kanchanaburi	Central	2007	2.2.1	MDR-TB	ERR13665969	
173	Kanchanaburi	Central	2009	2.2.1	MDR-TB	ERR13666176	
174	Kanchanaburi	Central	2009	2.2.1	MDR-TB	ERR13666870	
175	Kanchanaburi	Central	2009	2.2.1	MDR-TB	ERR13665940	
176	Kanchanaburi	Central	2010	2.2.1	MDR-TB	ERR13666778	
177	Kanchanaburi	Central	2011	2.2.1	MDR-TB	ERR13666113	
178	Kanchanaburi	Central	2011	2.2.1	MDR-TB	ERR13666296	
179	Kanchanaburi	Central	2011	2.2.1	MDR-TB	ERR13666443	
180	Kanchanaburi	Central	2003	2.2.1	MDR-TB	ERR13666628	
181	Kanchanaburi	Central	2003	2.2.1	MDR-TB	ERR13666301	
182	Kanchanaburi	Central	2004	2.2.1	Pre-XDR-TB	ERR13666183	
183	Kanchanaburi	Central	2004	4.4.2	MDR-TB	ERR13666513	
184	Kanchanaburi	Central	2005	2.2.1	MDR-TB	ERR13666659	
185	Kanchanaburi	Central	2005	2.2.1	MDR-TB	ERR13666218	
186	Kanchanaburi	Central	2005	2.2.1	MDR-TB	ERR13666535	
187	Kanchanaburi	Central	2005	2.2.1	MDR-TB	ERR13666843	
188	Kanchanaburi	Central	2006	2.2.1	MDR-TB	ERR13666676	
189	Kanchanaburi	Central	2006	2.2.1.1	RR-TB	ERR13666508	
190	Kanchanaburi	Central	2006	2.1	Pre-XDR-TB	ERR13666910	SRR11715722
191	Kanchanaburi	Central	2006	2.2.1	Other	ERR13665990	
192	Kanchanaburi	Central	2007	1.2.2.2	HR-TB	ERR13666222	
193	Kanchanaburi	Central	2007	2.2.1	MDR-TB	ERR13666748	
194	Kanchanaburi	Central	2008	2.2.1	Pre-XDR-TB	ERR13666289	
195	Kanchanaburi	Central	2008	2.2.1	MDR-TB	ERR13666664	
196	Kanchanaburi	Central	2008	2.1	MDR-TB	ERR13666008	SRR11715721
197	Kanchanaburi	Central	2008	2.2.1	Pre-XDR-TB	ERR13666883	
198	Kanchanaburi	Central	2009	2.1	MDR-TB	ERR13666370	SRR11715719
199	Kanchanaburi	Central	2010	2.2.1	MDR-TB	ERR13665917	
200	Kanchanaburi	Central	2011	1.1.1	MDR-TB	ERR13665957	
201	Kanchanaburi	Central	2011	2.2.1	MDR-TB	ERR13666690	
202	Kanchanaburi	Central	2011	1.1.1	MDR-TB	ERR13666223	
203	Kanchanaburi	Central	2011	2.2.1	MDR-TB	ERR13666509	
204	Kanchanaburi	Central	2011	2.2.1	MDR-TB	ERR13665951	
205	Kanchanaburi	Central	2011	2.2.1	MDR-TB	ERR13666512	
206	Kanchanaburi	Central	2012	2.2.1	MDR-TB	ERR13666634	

207	Kanchanaburi	Central	2012	2.2.1	MDR-TB	ERR13665950	
208	Kanchanaburi	Central	2012	2.2.1	MDR-TB	ERR13666255	
209	Kanchanaburi	Central	2003	2.2.1	MDR-TB	ERR13666011	
210	Kanchanaburi	Central	2006	2.2.1	MDR-TB	ERR13666313	
211	Kanchanaburi	Central	2006	2.2.1	MDR-TB	ERR13666709	
212	Kanchanaburi	Central	2007	2.2.1	MDR-TB	ERR13666893	
213	Kanchanaburi	Central	2008	2.2.1	MDR-TB	ERR13665963	
214	Kanchanaburi	Central	2009	2.2.1	MDR-TB	ERR13666890	
215	Kanchanaburi	Central	2006	2.2.1	MDR-TB	ERR13665902	
216	Kanchanaburi	Central	2005	2.2.1	MDR-TB	ERR13666624	
217	Kanchanaburi	Central	2005	2.2.1	MDR-TB	ERR13666704	
218	Yala	Southern	2001	2.2.1.1	Pre-XDR-TB	ERR13665972	
219	Rayong	Central	2008	2.2.1	Pre-XDR-TB	ERR13665975	
220	Suphan Buri	Central	2009	2.2.1	MDR-TB	ERR13666615	
221	Surat Thani	Southern	2007	2.2.1	Pre-XDR-TB	ERR13666295	
222	Nong Khai	Northeastern	2007	1.1.3.1	RR-TB	ERR13666329	
223	Bangkok	Central	2008	1.2.2	MDR-TB	ERR13666763	
224	Kanchanaburi	Central	2005	2.1	Pre-XDR-TB	ERR13665912	SRR11715725
225	Bangkok	Central	2005	4.5	Pre-XDR-TB	ERR13666552	
226	Bangkok	Central	2009	2.2.1	MDR-TB	ERR13666353	
227	Bangkok	Central	2012	1.2.1.2	MDR-TB	ERR13666817	
228	Bangkok	Central	2015	2.2.1	MDR-TB	ERR13666192	
229	Bangkok	Central	2015	2.2.1	Pre-XDR-TB	ERR13665872	
230	Phrae	Northern	2015	2.2.1	HR-TB	ERR13665997	
231	Bangkok	Central	2015	2.2.1	Pre-XDR-TB	ERR13666572	
232	Bangkok	Central	2014	2.2.1.2	MDR-TB	ERR13666107	
233	Chachoengsao	Central	2005	2.2.1	HR-TB	ERR13665973	
234	Nakhon Phanom	Northeastern	2006	2.2.1.1	Pre-XDR-TB	ERR13666216	
235	Vakhon Ratchasima	Northeastern	2006	2.2.1	Pre-XDR-TB	ERR13666332	
236	Vakhon Ratchasima	Northeastern	2006	1.1	Pre-XDR-TB	ERR13666455	
237	Vakhon Ratchasima	Northeastern	2007	1.1	Pre-XDR-TB	ERR13666269	
238	Vakhon Ratchasima	Northeastern	2008	1.1.3.3	MDR-TB	ERR13666137	
239	Vakhon Ratchasima	Northeastern	2011	2.1	Pre-XDR-TB	ERR13666136	SRR11715716
240	Vakhon Ratchasima	Northeastern	2012	2.2.1	Pre-XDR-TB	ERR13666167	
241	Vakhon Ratchasima	Northeastern	2012	2.2.1	Pre-XDR-TB	ERR13666669	
242	Vakhon Ratchasima	Northeastern	2012	2.2.1	Pre-XDR-TB	ERR13666448	
243	Vakhon Ratchasima	Northeastern	2012	2.2.1	Pre-XDR-TB	ERR13666672	
244	Nonthaburi	Central	2005	2.2.1	MDR-TB	ERR13665993	
245	Nonthaburi	Central	2008	1.2.1.2.1	Sensitive	ERR13666500	
246	Nan	Northern	2007	2.2.1	MDR-TB	ERR13666612	
247	Nan	Northern	2006	2.2.1	MDR-TB	ERR13666318	
248	Buri Ram	Northeastern	2007	2.2.1	Pre-XDR-TB	ERR13666617	
249	Buri Ram	Northeastern	2007	2.2.1	Pre-XDR-TB	ERR13666583	
250	Bangkok	Central	2015	4.4.2	MDR-TB	ERR13666392	
251	Buri Ram	Northeastern	2015	1.1.1.1	MDR-TB	ERR13665966	
252	'rachuap Khiri Khar	Central	2005	2.2.1	RR-TB	ERR13666186	
253	Chon Buri	Central	2012	2.2.1	Pre-XDR-TB	ERR13666389	
254	Phitsanulok	Northern	2009	2.2.1	MDR-TB	ERR13666291	
255	Phrae	Northern	2006	2.2.1	Pre-XDR-TB	ERR13666740	
256	Roi Et	Northeastern	2014	2.2.1	Pre-XDR-TB	ERR13666777	
257	Rayong	Central	2001	4.2.2	Pre-XDR-TB	ERR13666099	
258	Suphan Buri	Central	2010	2.2.1	MDR-TB	ERR13666547	

259	Suphan Buri	Central	2010	4.2.2	MDR-TB	ERR13666300
260	Samut Sakhon	Central	2002	2.2.1	MDR-TB	ERR13665984
261	Samut Sakhon	Central	2003	2.2.1	MDR-TB	ERR13666862
262	Samut Sakhon	Central	2004	2.2.1	MDR-TB	ERR13666570
263	Chiang Mai	Northern	2005	2.2.1	MDR-TB	ERR13665935
264	Chiang Mai	Northern	2005	2.2.1	MDR-TB	ERR13665921
265	Chiang Mai	Northern	2004	1.1.1	MDR-TB	ERR13666589
266	Satun	Southern	2015	1.1.1	MDR-TB	ERR13666863
267	Bangkok	Central	2015	2.2.1	MDR-TB	ERR13666891
268	Bangkok	Central	2015	2.2.1.1	RR-TB	ERR13666585
269	Surat Thani	Southern	2016	2.2.1	MDR-TB	ERR13666839
270	Bangkok	Central	2015	2.2.1	MDR-TB	ERR13666533
271	Bangkok	Central	2015	2.2.1	MDR-TB	ERR13666180
272	Buri Ram	Northeastern	2015	2.2.1	RR-TB	ERR13666261
273	Kanchanaburi	Central	2007	2.2.1	MDR-TB	ERR13666232
274	Buri Ram	Northeastern	2006	2.2.1	MDR-TB	ERR13666745
275	Chachoengsao	Central	2011	2.2.1.1	MDR-TB	ERR13666133
276	Chachoengsao	Central	2011	2.2.1	MDR-TB	ERR13666441
277	Chon Buri	Central	2011	1.2.1.2	Sensitive	ERR13666066
278	Chachoengsao	Central	2011	2.2.1	MDR-TB	ERR13666851
279	Chachoengsao	Central	2011	1.1.1	MDR-TB	ERR13665907
280	Buri Ram	Northeastern	2011	2.1	MDR-TB	ERR13665952
281	Bangkok	Central	2010	2.2.1	MDR-TB	ERR13666840
282	Nonthaburi	Central	2010	2.2.1	MDR-TB	ERR13666030
283	Rayong	Central	2010	2.2.1	MDR-TB	ERR13666597
284	Surat Thani	Southern	2010	2.2.1	Pre-XDR-TB	ERR13666622
285	Chachoengsao	Central	2010	2.2.1	MDR-TB	ERR13666355
286	Bangkok	Central	2010	2.2.1	MDR-TB	ERR13666308
287	Nonthaburi	Central	2010	2.2.1	MDR-TB	ERR13666278
288	Phrae	Northern	2010	2.2.1	MDR-TB	ERR13665976
289	Bangkok	Central	2010	2.2.1.1	MDR-TB	ERR13666414
290	Chachoengsao	Central	2010	2.2.1	Pre-XDR-TB	ERR13666177
291	Jakhon Ratchasima	Northeastern	2009	2.1	MDR-TB	ERR13666302
292	Bangkok	Central	2009	2.2.1	RR-TB	ERR13666498
293	Chachoengsao	Central	2009	2.2.1	MDR-TB	ERR13666766
294	Satun	Southern	2009	2.2.1	MDR-TB	ERR13666600
295	Bangkok	Central	2009	2.2.1	MDR-TB	ERR13666215
296	Sukhothai	Northern	2009	2.2.1	MDR-TB	ERR13666846
297	Nong Khai	Northeastern	2009	2.2.1.2	MDR-TB	ERR13666323
298	'rachuap Khiri Khar	Central	2009	2.2.1	MDR-TB	ERR13666390
299	Bangkok	Central	2009	1.1.1	MDR-TB	ERR13666235
300	Rayong	Central	2009	4.2.2	MDR-TB	ERR13666887
301	Satun	Southern	2009	2.2.1	MDR-TB	ERR13666379
302	Saraburi	Central	2009	2.1	MDR-TB	ERR13666794
303	Nonthaburi	Central	2008	2.2.1	MDR-TB	ERR13666285
304	Nonthaburi	Central	2008	2.2.1	MDR-TB	ERR13666849
305	Bangkok	Central	2008	2.2.1	MDR-TB	ERR13666716
306	Chachoengsao	Central	2008	2.2.1.1	MDR-TB	ERR13666750
307	Jakhon Ratchasima	Northeastern	2008	2.2.1	MDR-TB	ERR13666581
308	Chachoengsao	Central	2008	2.2.1	Pre-XDR-TB	ERR13666756
309	Bangkok	Central	2008	2.2.1	RR-TB	ERR13666375
310	Chachoengsao	Central	2008	2.2.1	MDR-TB	ERR13666156

311	Surat Thani	Southern	2008	2.2.1	MDR-TB	ERR13666376
312	Phitsanulok	Northern	2008	4.2.2	MDR-TB	ERR13666262
313	Buri Ram	Northeastern	2008	2.2.1	MDR-TB	ERR13666541
314	Nan	Northern	2008	2.2.1	MDR-TB	ERR13665956
315	Bangkok	Central	2007	2.2.1	Sensitive	ERR13666273
316	Chachoengsao	Central	2007	2.2.1.1	MDR-TB	ERR13666050
317	Bangkok	Central	2007	2.2.1	MDR-TB	ERR13666237
318	Rayong	Central	2007	2.2.1	MDR-TB	ERR13666909
319	Chachoengsao	Central	2007	2.2.1	MDR-TB	ERR13666804
320	Nan	Northern	2007	2.2.1	MDR-TB	ERR13666653
321	Chon Buri	Central	2007	2.2.1	Pre-XDR-TB	ERR13665874
322	Samut Prakan	Central	2002	2.2.1	MDR-TB	ERR13666826
323	Phetchabun	Northern	2002	2.2.1	MDR-TB	ERR13666684
324	Samut Prakan	Central	2002	4.2.2	MDR-TB	ERR13666837
325	Nan	Northern	2002	2.2.1	MDR-TB	ERR13665943
326	Nonthaburi	Central	2003	2.2.1	HR-TB	ERR13666334
327	Bangkok	Central	2003	2.2.1	MDR-TB	ERR13666320
328	Bangkok	Central	2003	2.2.1	MDR-TB	ERR13666331
329	Bangkok	Central	2003	2.2.1	MDR-TB	ERR13665922
330	Bangkok	Central	2003	2.2.1	MDR-TB	ERR13666772
331	Bangkok	Central	2003	2.2.1	MDR-TB	ERR13666660
332	Chachoengsao	Central	2003	2.2.1	MDR-TB	ERR13666242
333	Samut Prakan	Central	2003	2.2.1	MDR-TB	ERR13666438
334	Chachoengsao	Central	2003	2.2.1	MDR-TB	ERR13666383
335	Chon Buri	Central	2003	2.2.1	MDR-TB	ERR13666126
336	Chachoengsao	Central	2003	2.2.1.1	MDR-TB	ERR13666205
337	Nonthaburi	Central	2004	2.2.1	MDR-TB	ERR13665885
338	Phitsanulok	Northern	2004	2.2.1	MDR-TB	ERR13666643
339	Bangkok	Central	2004	2.2.1	MDR-TB	ERR13666908
340	Bangkok	Central	2004	4.2.2	MDR-TB	ERR13666907
341	Loei	Northeastern	2004	4.4.2	MDR-TB	ERR13666736
342	Rayong	Central	2004	2.2.1	MDR-TB	ERR13666129
343	Loei	Northeastern	2005	4.4.2	MDR-TB	ERR13666116
344	Chon Buri	Central	2005	2.2.1	MDR-TB	ERR13666037
345	Phetchabun	Northern	2005	2.2.1	MDR-TB	ERR13666478
346	Rayong	Central	2005	2.2.1	MDR-TB	ERR13666016
347	Chachoengsao	Central	2005	2.2.1	MDR-TB	ERR13666403
348	Bangkok	Central	2005	2.2.1	MDR-TB	ERR13666155
349	Buri Ram	Northeastern	2005	2.2.1	MDR-TB	ERR13666354
350	Nonthaburi	Central	2006	2.2.1	MDR-TB	ERR13666779
351	Rayong	Central	2006	2.2.1	MDR-TB	ERR13666831
352	Rayong	Central	2006	2.2.1	MDR-TB	ERR13666904
353	Chumphon	Southern	2006	1.2.1.2.1	MDR-TB	ERR13666363
354	\akhon Ratchasima	Northeastern	2006	2.2.1.1	MDR-TB	ERR13665918
355	Bangkok	Central	2006	2.2.1	MDR-TB	ERR13666651
356	\akhon Ratchasima	Northeastern	2006	2.2.1	RR-TB	ERR13666256
357	Rayong	Central	2006	2.2.1	MDR-TB	ERR13666090
358	Rayong	Central	2006	2.2.1	MDR-TB	ERR13666169
359	Nonthaburi	Central	2006	2.2.1	MDR-TB	ERR13666859
360	Buri Ram	Northeastern	2006	2.2.1	MDR-TB	ERR13666569
361	Nan	Northern	2006	2.2.1	MDR-TB	ERR13666082
362	Rayong	Central	2006	2.2.1	MDR-TB	ERR13665905

363	\akhon Ratchasima	Northeastern	2006	2.2.1	Pre-XDR-TB	ERR13665948
364	Chon Buri	Central	2006	4.2.2	MDR-TB	ERR13666051
365	\akhon Ratchasima	Northeastern	2006	4.5	MDR-TB	ERR13666328
366	Phrae	Northern	2014	2.2.1.1	MDR-TB	ERR13666706
367	Songkhla	Southern	2006	2.2.1	Pre-XDR-TB	ERR13666028
368	Songkhla	Southern	2008	2.2.1	MDR-TB	ERR13666240
369	Songkhla	Southern	2010	2.2.1	MDR-TB	ERR13666903
370	Phitsanulok	Northern	2003	2.2.1	MDR-TB	ERR13665881
371	Prachin Buri	Central	2003	2.2.2	MDR-TB	ERR13666497
372	Phuket	Southern	2011	2.2.1	MDR-TB	ERR13666070
373	Surat Thani	Southern	2011	2.2.1	MDR-TB	ERR13666881
374	Phitsanulok	Northern	2011	2.2.1	MDR-TB	ERR13666275
375	Phitsanulok	Northern	2012	2.1	MDR-TB	ERR13666820
376	Phuket	Southern	2012	2.2.1.1	MDR-TB	ERR13666106
377	Suphan Buri	Central	2012	2.2.1	Sensitive	ERR13666119
378	Phuket	Southern	2012	2.2.1.1	MDR-TB	ERR13665953
379	Phuket	Southern	2012	2.2.1	MDR-TB	ERR13666697
380	Saraburi	Central	2012	2.2.1	MDR-TB	ERR13666198
381	Suphan Buri	Central	2012	2.1	MDR-TB	ERR13666635
382	Suphan Buri	Central	2012	2.2.1	MDR-TB	ERR13665909
383	Suphan Buri	Central	2012	2.2.1	MDR-TB	ERR13666663
384	Suphan Buri	Central	2012	2.2.1	MDR-TB	ERR13666717
385	Surat Thani	Southern	2012	2.2.1	MDR-TB	ERR13666879
386	Phatthalung	Southern	2012	2.2.1	MDR-TB	ERR13666171
387	'rachuap Khiri Khar	Central	2012	2.2.1	MDR-TB	ERR13666344
388	Phitsanulok	Northern	2012	2.2.1	MDR-TB	ERR13666598
389	Phuket	Southern	2012	2.2.1	MDR-TB	ERR13666900
390	Phitsanulok	Northern	2006	2.2.1	MDR-TB	ERR13666466
391	Phitsanulok	Northern	2006	2.2.1	MDR-TB	ERR13666590
392	Saraburi	Central	2005	2.2.1	MDR-TB	ERR13666083
393	Nong Khai	Northeastern	2014	2.2.1	MDR-TB	ERR13666080
394	Lop Buri	Central	2014	2.2.1	MDR-TB	ERR13666626
395	Nong Khai	Northeastern	2015	1.1.3.1	MDR-TB	ERR13666678
396	Bangkok	Central	2001	2.2.1	MDR-TB	ERR13666118
397	Surat Thani	Southern	2001	2.2.1	MDR-TB	ERR13666787
398	Yala	Southern	2001	2.2.1	MDR-TB	ERR13666760
399	Rayong	Central	2001	4.5	MDR-TB	ERR13666464
400	Suphan Buri	Central	2003	2.2.1	MDR-TB	ERR13666725
401	Nong Khai	Northeastern	2006	2.1	MDR-TB	ERR13666424
402	Phitsanulok	Northern	2007	2.2.1	MDR-TB	ERR13666147
403	Suphan Buri	Central	2010	1.1.3.3	MDR-TB	ERR13666377
404	Nong Khai	Northeastern	2011	2.2.1	MDR-TB	ERR13666661
405	Nong Khai	Northeastern	2011	1.1.3.3	MDR-TB	ERR13666462
406	Nong Khai	Northeastern	2011	2.2.1	MDR-TB	ERR13666906
407	Nong Khai	Northeastern	2011	1.1.3.3	MDR-TB	ERR13666616
408	\akhon Ratchasima	Northeastern	2012	2.1	MDR-TB	ERR13666431
409	\akhon Ratchasima	Northeastern	2012	2.2.1	MDR-TB	ERR13665967
410	Bangkok	Central	2012	2.2.1	MDR-TB	ERR13666493
411	Bangkok	Central	2012	2.2.1	MDR-TB	ERR13666573
412	Nonthaburi	Central	2012	2.2.1	MDR-TB	ERR13666808
413	Nonthaburi	Central	2012	2.2.1	MDR-TB	ERR13666283
414	\akhon Ratchasima	Northeastern	2012	2.2.1	MDR-TB	ERR13666472

415	Bangkok	Central	2012	2.2.1	MDR-TB	ERR13666315	
416	Buri Ram	Northeastern	2012	2.2.1	MDR-TB	ERR13666357	
417	Roi Et	Northeastern	2012	2.2.1.1	MDR-TB	ERR13666789	
418	Buri Ram	Northeastern	2012	2.2.1	MDR-TB	ERR13666151	
419	Nakhon Ratchasima	Northeastern	2012	2.1	MDR-TB	ERR13666658	
420	Chumphon	Southern	2012	2.2.1	MDR-TB	ERR13666614	
421	Nakhon Ratchasima	Northeastern	2012	2.2.1	MDR-TB	ERR13666321	
422	Roi Et	Northeastern	2012	1.1.1	MDR-TB	ERR13666743	
423	Bangkok	Central	2012	2.2.1	MDR-TB	ERR13666729	
424	Nakhon Ratchasima	Northeastern	2012	2.1	MDR-TB	ERR13666834	
425	Phrae	Northern	2012	2.2.2	MDR-TB	ERR13666214	
426	Buri Ram	Northeastern	2012	2.2.1	MDR-TB	ERR13666393	
427	Nong Khai	Northeastern	2012	2.2.1	MDR-TB	ERR13666797	
428	Satun	Southern	2012	1.1.3.3	MDR-TB	ERR13666061	
429	Nonthaburi	Central	2012	1.1.1	MDR-TB	ERR13666785	
430	Buri Ram	Northeastern	2012	2.2.1	MDR-TB	ERR13665977	
431	Nakhon Ratchasima	Northeastern	2012	2.2.1	RR-TB	ERR13666204	
432	Bangkok	Central	2012	2.2.1	Pre-XDR-TB	ERR13666728	
433	Nakhon Ratchasima	Northeastern	2012	2.2.1	MDR-TB	ERR13666546	
434	Bangkok	Central	2012	2.2.1	MDR-TB	ERR13666499	
435	Nonthaburi	Central	2012	2.2.1	MDR-TB	ERR13666253	
436	Bangkok	Central	2016	2.1	MDR-TB	ERR13666266	
437	Bangkok	Central	2016	2.2.1	MDR-TB	ERR13666631	
438	Bangkok	Central	2016	2.2.1	MDR-TB	ERR13666595	
439	Bangkok	Central	2016	2.2.1	MDR-TB	ERR13666847	
440	Bangkok	Central	2016	2.2.1	MDR-TB	ERR13666034	
441	Nakhon Ratchasima	Northeastern	2013	2.2.1	RR-TB	ERR13666482	
442	Phuket	Southern	2013	2.2.1	MDR-TB	ERR13666722	
443	Bangkok	Central	2013	2.2.1	Pre-XDR-TB	ERR13666823	
444	Roi Et	Northeastern	2013	2.2.1	MDR-TB	ERR13666114	
445	Bangkok	Central	2013	2.2.1	MDR-TB	ERR13666408	
446	Nonthaburi	Central	2014	2.2.1	MDR-TB	ERR13666866	
447	Nonthaburi	Central	2014	2.2.1	MDR-TB	ERR13666209	
448	Chumphon	Southern	2011	2.1	Sensitive	ERR13666457	SRR17610984
449	Buri Ram	Northeastern	2011	1.1.2	MDR-TB	ERR13666744	SRR17610983
450	Chachoengsao	Central	2011	2.2.1	MDR-TB	ERR13666021	SRR17610982
451	Nakhon Ratchasima	Northeastern	2011	2.2.1	MDR-TB	ERR13666279	SRR17610980
452	Nakhon Ratchasima	Northeastern	2011	2.2.2	MDR-TB	ERR13666710	SRR17610979
453	Phatthalung	Southern	2011	1.1.3.3	MDR-TB	ERR13666505	SRR17610978
454	Nakhon Ratchasima	Northeastern	2011	2.1	MDR-TB	ERR13666623	SRR17610977
455	Bangkok	Central	2011	2.2.1	MDR-TB	ERR13666687	SRR17610976
456	Bangkok	Central	2011	2.2.1	MDR-TB	ERR13666523	SRR17610975
457	Buri Ram	Northeastern	2011	1.1.2	MDR-TB	ERR13666529	SRR17610974
458	Bangkok	Central	2011	2.1	MDR-TB	ERR13666596	SRR17610973
459	Bangkok	Central	2011	1.1.1	MDR-TB	ERR13666775	SRR17611239
460	Nakhon Ratchasima	Northeastern	2011	2.2.1	Pre-XDR-TB	ERR13666157	SRR17611238
461	Nonthaburi	Central	2011	2.2.1	MDR-TB	ERR13666415	SRR17611236
462	Bangkok	Central	2011	2.2.1	MDR-TB	ERR13666252	SRR17611235
463	Pattani	Southern	2011	1.1.1	HR-TB	ERR13666166	SRR17611234
464	Bangkok	Central	2011	2.2.1	MDR-TB	ERR13666131	SRR17611233
465	Nakhon Ratchasima	Northeastern	2011	1.1.3.3	MDR-TB	ERR13666470	SRR17611232
466	Nakhon Ratchasima	Northeastern	2011	2.2.1	MDR-TB	ERR13666495	SRR17611231

467	Roi Et	Northeastern	2011	2.2.1	MDR-TB	ERR13666695	SRR17611230
468	Buri Ram	Northeastern	2011	1.1.2	MDR-TB	ERR13666856	SRR17611229
469	Nakhon Ratchasima	Northeastern	2011	2.2.1	Pre-XDR-TB	ERR13665989	SRR17611227
470	Tak	Northern	2011	2.2.1	RR-TB	ERR13666163	SRR17611224
471	Buri Ram	Northeastern	2011	2.2.1.1	MDR-TB	ERR13666416	SRR17611223
472	Buri Ram	Northeastern	2011	2.2.1	MDR-TB	ERR13665991	SRR17611222
473	Buri Ram	Northeastern	2011	2.2.1	MDR-TB	ERR13666294	SRR17611221
474	Buri Ram	Northeastern	2011	1.1.2	MDR-TB	ERR13666814	SRR17611220
475	Nakhon Ratchasima	Northeastern	2011	2.2.1	MDR-TB	ERR13666367	SRR17611219
476	Buri Ram	Northeastern	2011	1.1.1	MDR-TB	ERR13666299	SRR17611218
477	Phrae	Northern	2011	2.2.1	RR-TB	ERR13666049	SRR17611217
478	Bangkok	Central	2016	2.2.1	MDR-TB	ERR13666233	SRR17611216
479	Bangkok	Central	2012	2.2.1	MDR-TB	ERR13666146	SRR17611047
480	Nakhon Ratchasima	Northeastern	2012	2.2.1	MDR-TB	ERR13665891	SRR17611045
481	Buri Ram	Northeastern	2012	2.2.1	MDR-TB	ERR13666816	SRR17611044
482	Chumphon	Southern	2012	1.1.3.3	MDR-TB	ERR13666480	SRR17611043
483	Rayong	Central	2012	2.2.1	MDR-TB	ERR13666557	SRR17611042
484	Nakhon Ratchasima	Northeastern	2012	2.2.1	MDR-TB	ERR13666307	SRR17611041
485	Bangkok	Central	2012	2.2.1	MDR-TB	ERR13666040	SRR17611040
486	Nonthaburi	Central	2012	2.2.1	MDR-TB	ERR13665892	SRR17611039
487	Bangkok	Central	2012	2.2.1	MDR-TB	ERR13666025	SRR17611038
488	Nonthaburi	Central	2012	2.2.1	MDR-TB	ERR13666611	SRR17611037
489	Rayong	Central	2012	2.2.1	MDR-TB	ERR13666210	SRR17611036
490	Bangkok	Central	2012	2.2.1	MDR-TB	ERR13666140	SRR17611034
491	Buri Ram	Northeastern	2012	2.2.1	Pre-XDR-TB	ERR13666506	SRR17611033
492	Nakhon Ratchasima	Northeastern	2012	2.2.1	MDR-TB	ERR13666014	SRR17611032
493	Bangkok	Central	2012	2.2.1	MDR-TB	ERR13666491	SRR17611031
494	Bangkok	Central	2012	2.1	HR-TB	ERR13666681	SRR17611030
495	Nakhon Ratchasima	Northeastern	2012	2.2.1	MDR-TB	ERR13666004	SRR17611029
496	Buri Ram	Northeastern	2012	2.2.1.1	MDR-TB	ERR13666514	SRR17611028
497	Nakhon Ratchasima	Northeastern	2012	2.2.1	MDR-TB	ERR13666860	SRR17611027
498	Roi Et	Northeastern	2012	2.2.1	MDR-TB	ERR13665924	SRR17611026
499	Roi Et	Northeastern	2012	2.2.1	MDR-TB	ERR13665884	SRR17611025
500	Buri Ram	Northeastern	2012	2.2.1	MDR-TB	ERR13666404	SRR17610900
501	Nonthaburi	Central	2012	2.2.1	MDR-TB	ERR13666645	SRR17610899
502	Nakhon Ratchasima	Northeastern	2012	2.2.1	MDR-TB	ERR13666002	SRR17610898
503	Yala	Southern	2012	1.1.3.3	MDR-TB	ERR13666276	SRR17610897
504	Phrae	Northern	2012	2.2.1	MDR-TB	ERR13666667	SRR17610896
505	Chumphon	Southern	2012	1.1.1	MDR-TB	ERR13666503	SRR17610895
506	Buri Ram	Northeastern	2012	2.2.1	MDR-TB	ERR13666013	SRR17610894
507	Roi Et	Northeastern	2012	2.2.1	MDR-TB	ERR13666829	SRR17610893
508	Nakhon Ratchasima	Northeastern	2012	2.2.1	MDR-TB	ERR13666191	SRR17610892
509	Phrae	Northern	2012	2.1	MDR-TB	ERR13665961	SRR17610891
510	Nakhon Ratchasima	Northeastern	2012	2.1	MDR-TB	ERR13666142	SRR17610889
511	Buri Ram	Northeastern	2012	2.2.1	MDR-TB	ERR13666776	SRR17610888
512	Bangkok	Central	2012	2.2.1	MDR-TB	ERR13666201	SRR17610887
513	Bangkok	Central	2012	2.2.1	MDR-TB	ERR13666073	SRR17610886
514	Nakhon Ratchasima	Northeastern	2012	2.2.1	MDR-TB	ERR13666292	SRR17610885
515	Ratchaburi	Central	2012	2.1	Pre-XDR-TB	ERR13666727	SRR17610884
516	Bangkok	Central	2012	2.2.1.1	MDR-TB	ERR13666220	SRR17610883
517	Bangkok	Central	2016	2.2.1	MDR-TB	ERR13666122	SRR17610882
518	Phuket	Southern	2013	2.2.1.1	MDR-TB	ERR13666440	SRR17610881

519	Bangkok	Central	2013	2.2.1	MDR-TB	ERR13666213	SRR17610880
520	Chumphon	Southern	2013	2.2.1	Pre-XDR-TB	ERR13666268	SRR17610878
521	Suphan Buri	Central	2013	2.2.1	MDR-TB	ERR13666463	SRR17610877
522	Nakhon Ratchasima	Northeastern	2013	2.2.1	Pre-XDR-TB	ERR13666637	SRR17611095
523	Buri Ram	Northeastern	2013	2.2.1	MDR-TB	ERR13666486	SRR17611094
524	Suphan Buri	Central	2013	2.2.1	MDR-TB	ERR13666526	SRR17611093
525	Buri Ram	Northeastern	2013	2.2.1	MDR-TB	ERR13666287	SRR17611092
526	Nong Khai	Northeastern	2013	2.2.2	MDR-TB	ERR13666769	SRR17611091
527	Chumphon	Southern	2013	3	Sensitive	ERR13666530	SRR17611090
528	Buri Ram	Northeastern	2013	2.2.1	MDR-TB	ERR13666125	SRR17611089
529	Bangkok	Central	2013	2.2.1	MDR-TB	ERR13666293	SRR17611088
530	Bangkok	Central	2013	2.2.1	MDR-TB	ERR13665882	SRR17611086
531	Bangkok	Central	2013	2.2.1	MDR-TB	ERR13666358	SRR17611085
532	Suphan Buri	Central	2013	2.2.1	MDR-TB	ERR13666134	SRR17611084
533	Nakhon Ratchasima	Northeastern	2013	1.1.1	MDR-TB	ERR13666741	SRR17611083
534	Bangkok	Central	2013	2.2.1	MDR-TB	ERR13666815	SRR17611082
535	Chon Buri	Central	2013	1.2.1.2	HR-TB	ERR13666339	SRR17611081
536	Nonthaburi	Central	2013	2.2.1	MDR-TB	ERR13666033	SRR17611080
537	Nakhon Ratchasima	Northeastern	2013	2.2.1	MDR-TB	ERR13666792	SRR17611079
538	Surat Thani	Southern	2013	2.2.1	MDR-TB	ERR13666374	SRR17611078
539	Bangkok	Central	2013	2.2.1	RR-TB	ERR13666115	SRR17611077
540	Nakhon Ratchasima	Northeastern	2013	2.2.1	MDR-TB	ERR13665908	SRR17611075
541	Buri Ram	Northeastern	2013	2.2.1.1	MDR-TB	ERR13665999	SRR17611074
542	Prachuap Khiri Khan	Central	2013	1.1.2	MDR-TB	ERR13666100	SRR17611073
543	Ratchaburi	Central	2013	4.4.2	MDR-TB	ERR13666625	SRR17611072
544	Satun	Southern	2013	2.2.1	RR-TB	ERR13666714	SRR17611215
545	Bangkok	Central	2013	4.1.2.1	MDR-TB	ERR13666827	SRR17611214
546	Bangkok	Central	2013	2.2.1	MDR-TB	ERR13666629	SRR17611213
547	Bangkok	Central	2013	2.2.1	MDR-TB	ERR13665895	SRR17611212
548	Ratchaburi	Central	2013	2.2.1	MDR-TB	ERR13666227	SRR17611211
549	Ratchaburi	Central	2013	2.2.1	MDR-TB	ERR13666865	SRR17611210
550	Saraburi	Central	2013	2.2.1	MDR-TB	ERR13666892	SRR17611208
551	Bangkok	Central	2013	2.2.1	MDR-TB	ERR13666647	SRR17611207
552	Satun	Southern	2013	2.2.1	RR-TB	ERR13666467	SRR17611206
553	Nonthaburi	Central	2013	2.2.1	MDR-TB	ERR13666877	SRR17611205
554	Surat Thani	Southern	2013	2.2.1	MDR-TB	ERR13666088	SRR17611204
555	Phitsanulok	Northern	2013	2.2.1	Pre-XDR-TB	ERR13665875	SRR17611203
556	Buri Ram	Northeastern	2013	2.2.1	MDR-TB	ERR13666340	SRR17611202
557	Bangkok	Central	2013	2.2.1	MDR-TB	ERR13666093	SRR17611201
558	Roi Et	Northeastern	2013	2.2.1	MDR-TB	ERR13666670	SRR17611200
559	Satun	Southern	2013	1.1.3.3	MDR-TB	ERR13666068	SRR17611199
560	Chon Buri	Central	2013	2.2.1	MDR-TB	ERR13666202	SRR17611197
561	Sukhothai	Northern	2013	2.1	MDR-TB	ERR13665987	SRR17611196
562	Surat Thani	Southern	2013	2.2.1	HR-TB	ERR13666507	SRR17611195
563	Roi Et	Northeastern	2013	2.2.1	MDR-TB	ERR13666130	SRR17611194
564	Bangkok	Central	2013	2.2.1	MDR-TB	ERR13665911	SRR17611193
565	Saraburi	Central	2014	4.4.2	MDR-TB	ERR13666128	SRR17611192
566	Nonthaburi	Central	2014	2.2.1	MDR-TB	ERR13666149	SRR17611167
567	Bangkok	Central	2014	2.2.1	MDR-TB	ERR13666031	SRR17611166
568	Saraburi	Central	2014	2.2.1	MDR-TB	ERR13666193	SRR17611165
569	Uthai Thani	Northern	2014	4.3.3	MDR-TB	ERR13666668	SRR17611164
570	Buri Ram	Northeastern	2014	1.2.1.2.1	MDR-TB	ERR13666828	SRR17611161

571	Suphan Buri	Central	2014	2.2.1	MDR-TB	ERR13666673	SRR17611160
572	Buri Ram	Northeastern	2014	2.2.1	MDR-TB	ERR13666411	SRR17611159
573	Roi Et	Northeastern	2014	4.5	Pre-XDR-TB	ERR13665894	SRR17611158
574	Ratchaburi	Central	2014	2.1	Pre-XDR-TB	ERR13666337	SRR17611157
575	Buri Ram	Northeastern	2014	2.2.1	MDR-TB	ERR13666419	SRR17611156
576	Bangkok	Central	2014	2.2.1	MDR-TB	ERR13666310	SRR17611155
577	Buri Ram	Northeastern	2014	2.2.1	MDR-TB	ERR13666703	SRR17611154
578	Buri Ram	Northeastern	2014	2.2.1	MDR-TB	ERR13666352	SRR17611153
579	Buri Ram	Northeastern	2014	2.2.1	MDR-TB	ERR13666065	SRR17611152
580	Nonthaburi	Central	2014	2.2.1	MDR-TB	ERR13666896	SRR17611150
581	Buri Ram	Northeastern	2014	2.2.1	MDR-TB	ERR13666396	SRR17611149
582	Buri Ram	Northeastern	2014	2.2.1	MDR-TB	ERR13666520	SRR17611147
583	Buri Ram	Northeastern	2014	2.2.1	MDR-TB	ERR13666751	SRR17611146
584	Buri Ram	Northeastern	2014	2.2.1	MDR-TB	ERR13666270	SRR17611145
585	Nonthaburi	Central	2014	2.2.1	MDR-TB	ERR13665920	SRR17611144
586	Buri Ram	Northeastern	2014	2.2.1	MDR-TB	ERR13666284	SRR17610852
587	Roi Et	Northeastern	2014	4.5	Pre-XDR-TB	ERR13666067	SRR17610851
588	Phrae	Northern	2014	2.2.1	MDR-TB	ERR13666825	SRR17610850
589	Buri Ram	Northeastern	2014	2.2.1	MDR-TB	ERR13666387	SRR17610848
590	Bangkok	Central	2014	2.2.1	MDR-TB	ERR13666790	SRR17610847
591	Buri Ram	Northeastern	2014	1.1.1	MDR-TB	ERR13666620	SRR17610846
592	Bangkok	Central	2014	2.2.1	MDR-TB	ERR13666665	SRR17610845
593	Kanchanaburi	Central	2014	2.2.1	MDR-TB	ERR13666074	SRR17610844
594	Satun	Southern	2014	1.1.1	MDR-TB	ERR13666644	SRR17610843
595	Bangkok	Central	2014	2.2.1	MDR-TB	ERR13666873	SRR17610842
596	Buri Ram	Northeastern	2014	2.2.1	MDR-TB	ERR13666035	SRR17610841
597	Nonthaburi	Central	2014	2.2.1.1	MDR-TB	ERR13666430	SRR17610840
598	Phrae	Northern	2014	2.2.1	MDR-TB	ERR13666062	SRR17610839
599	Roi Et	Northeastern	2014	4.5	Pre-XDR-TB	ERR13666565	SRR17610837
600	Nonthaburi	Central	2014	2.2.1	MDR-TB	ERR13665955	SRR17610836
601	Satun	Southern	2014	2.2.1	MDR-TB	ERR13666705	SRR17610835
602	Nong Khai	Northeastern	2014	2.2.1	MDR-TB	ERR13666168	SRR17610834
603	Satun	Southern	2014	2.2.1	MDR-TB	ERR13666818	SRR17610833
604	Phrae	Northern	2014	2.2.1	MDR-TB	ERR13665962	SRR17610832
605	Buri Ram	Northeastern	2014	2.2.1	MDR-TB	ERR13666381	SRR17610831
606	Buri Ram	Northeastern	2014	1.1.1	MDR-TB	ERR13666079	SRR17610830
607	Buri Ram	Northeastern	2014	2.2.1	MDR-TB	ERR13666764	SRR17610829
608	Buri Ram	Northeastern	2014	1.1.1	MDR-TB	ERR13666605	SRR17611119
609	Surat Thani	Southern	2014	2.2.1	MDR-TB	ERR13666084	SRR17611117
610	Bangkok	Central	2014	2.2.1	MDR-TB	ERR13666861	SRR17611116
611	Bangkok	Central	2014	2.2.1	Pre-XDR-TB	ERR13666405	SRR17611115
612	Nonthaburi	Central	2014	2.2.1	MDR-TB	ERR13666144	SRR17611114
613	Buri Ram	Northeastern	2014	2.2.1	MDR-TB	ERR13666880	SRR17611113
614	Bangkok	Central	2014	2.2.1	MDR-TB	ERR13666445	SRR17611112
615	Buri Ram	Northeastern	2014	2.2.1	MDR-TB	ERR13665903	SRR17611111
616	Nonthaburi	Central	2014	2.1	MDR-TB	ERR13666636	SRR17611110
617	Bangkok	Central	2014	2.1	MDR-TB	ERR13666196	SRR17611109
618	Lop Buri	Central	2014	2.2.1	MDR-TB	ERR13666208	SRR17611108
619	Buri Ram	Northeastern	2015	2.2.1.1	Pre-XDR-TB	ERR13666897	SRR17611106
620	Buri Ram	Northeastern	2015	4.2.2	MDR-TB	ERR13666420	SRR17611104
621	Satun	Southern	2015	1.1.3.3	MDR-TB	ERR13666702	SRR17611103
622	Satun	Southern	2015	2.2.1	MDR-TB	ERR13666501	SRR17611102

623	Surat Thani	Southern	2015	2.2.1	MDR-TB	ERR13666528	SRR17611101
624	Bangkok	Central	2015	2.2.1	Pre-XDR-TB	ERR13666485	SRR17611100
625	Bangkok	Central	2015	2.2.1	MDR-TB	ERR13666433	SRR17611098
626	Buri Ram	Northeastern	2015	2.2.1	MDR-TB	ERR13666894	SRR17611097
627	Suphan Buri	Central	2015	2.2.1	MDR-TB	ERR13666844	SRR17611071
628	Buri Ram	Northeastern	2015	2.2.1	MDR-TB	ERR13665938	SRR17611070
629	Lop Buri	Central	2015	2.2.1	MDR-TB	ERR13666753	SRR17611069
630	Bangkok	Central	2015	2.2.1	MDR-TB	ERR13666698	SRR17611068
631	Buri Ram	Northeastern	2015	1.1.1	MDR-TB	ERR13666359	SRR17611067
632	Chumphon	Southern	2015	2.2.1	MDR-TB	ERR13666527	SRR17611066
633	Roi Et	Northeastern	2015	2.2.1	MDR-TB	ERR13666810	SRR17611065
634	Buri Ram	Northeastern	2015	2.2.1	MDR-TB	ERR13666378	SRR17611064
635	Buri Ram	Northeastern	2015	1.1.1	MDR-TB	ERR13666101	SRR17611063
636	Satun	Southern	2015	1.1.3.3	MDR-TB	ERR13666518	SRR17611062
637	Bangkok	Central	2015	2.2.1	MDR-TB	ERR13665944	SRR17611060
638	Buri Ram	Northeastern	2015	2.2.1	MDR-TB	ERR13666822	SRR17611059
639	Satun	Southern	2016	1.1.3	MDR-TB	ERR13666655	SRR17611058
640	Nong Khai	Northeastern	2015	4.5	MDR-TB	ERR13666707	SRR17611057
641	Buri Ram	Northeastern	2015	2.2.1	MDR-TB	ERR13665889	SRR17611056
642	Bangkok	Central	2015	2.1	MDR-TB	ERR13666023	SRR17611055
643	Roi Et	Northeastern	2015	2.2.1	MDR-TB	ERR13666394	SRR17611054
644	Bangkok	Central	2015	2.2.1	MDR-TB	ERR13666042	SRR17611053
645	Surat Thani	Southern	2015	1.1.1	MDR-TB	ERR13666421	SRR17611052
646	Buri Ram	Northeastern	2015	2.2.1	MDR-TB	ERR13666838	SRR17611051
647	Satun	Southern	2015	2.2.1	MDR-TB	ERR13666325	SRR17611049
648	Bangkok	Central	2015	2.2.1.1	RR-TB	ERR13666474	SRR17611048
649	Buri Ram	Northeastern	2015	2.1	MDR-TB	ERR13665983	SRR17611191
650	Buri Ram	Northeastern	2015	1.1.1	RR-TB	ERR13665941	SRR17611190
651	Bangkok	Central	2015	2.2.1	MDR-TB	ERR13665919	SRR17611189
652	Bangkok	Central	2015	2.2.1	MDR-TB	ERR13666594	SRR17611188
653	Buri Ram	Northeastern	2015	2.2.1	Pre-XDR-TB	ERR13666009	SRR17611187
654	Buri Ram	Northeastern	2015	1.1.1.1	MDR-TB	ERR13666260	SRR17611186
655	Nong Khai	Northeastern	2015	2.2.1	MDR-TB	ERR13666712	SRR17611185
656	Phrae	Northern	2015	2.1	Pre-XDR-TB	ERR13666236	SRR17611184
657	Uthai Thani	Northern	2015	2.2.1	MDR-TB	ERR13666746	SRR17611182
658	Bangkok	Central	2015	2.2.1	MDR-TB	ERR13666012	SRR17611181
659	Phrae	Northern	2015	2.2.1	MDR-TB	ERR13666473	SRR17611180
660	Buri Ram	Northeastern	2015	1.1.1	MDR-TB	ERR13666076	SRR17611179
661	Surat Thani	Southern	2015	2.2.1	MDR-TB	ERR13665986	SRR17611178
662	Bangkok	Central	2015	2.2.1.1	MDR-TB	ERR13666648	SRR17611177
663	Bangkok	Central	2015	2.2.1	MDR-TB	ERR13666052	SRR17611176
664	Bangkok	Central	2015	2.2.1	Pre-XDR-TB	ERR13666435	SRR17611175
665	Nonthaburi	Central	2015	2.2.1	Pre-XDR-TB	ERR13666195	SRR17611174
666	Bangkok	Central	2015	2.2.1	MDR-TB	ERR13666110	SRR17611173
667	Bangkok	Central	2015	2.2.1	MDR-TB	ERR13666026	SRR17611170
668	Bangkok	Central	2015	2.2.1	Pre-XDR-TB	ERR13666782	SRR17611169
669	Roi Et	Northeastern	2015	2.2.1	MDR-TB	ERR13666489	SRR17611168
670	Bangkok	Central	2015	2.2.1	MDR-TB	ERR13666564	SRR17610924
671	Bangkok	Central	2015	2.2.1	MDR-TB	ERR13666089	SRR17610923
672	Buri Ram	Northeastern	2015	2.2.1	MDR-TB	ERR13666786	SRR17610922
673	Bangkok	Central	2016	2.2.1	RR-TB	ERR13665959	SRR17610921
674	Phrae	Northern	2016	2.2.1	HR-TB	ERR13666135	SRR17610920

675	Bangkok	Central	2016	2.2.1	RR-TB	ERR13665964	SRR17610919
676	Buri Ram	Northeastern	2016	1.2.1.2.1	MDR-TB	ERR13665930	SRR17610918
677	Bangkok	Central	2016	2.2.1	MDR-TB	ERR13666388	SRR17610916
678	Roi Et	Northeastern	2016	2.2.1.1	MDR-TB	ERR13666699	SRR17610915
679	Satun	Southern	2016	2.2.1	MDR-TB	ERR13666475	SRR17610914
680	Nong Khai	Northeastern	2016	2.2.1	MDR-TB	ERR13666229	SRR17610913
681	Kanchanaburi	Central	2012	1.1.1	MDR-TB	ERR13666290	SRR17610912
682	Loei	Northeastern	2002	4.4.2	MDR-TB	ERR13666632	SRR17610911
683	Surat Thani	Southern	2003	1.2.1.2.1	MDR-TB	ERR13666444	SRR17610910
684	Buri Ram	Northeastern	2003	1.1.2	HR-TB	ERR13666231	SRR17610909
685	Chachoengsao	Central	2003	2.2.1	MDR-TB	ERR13665932	SRR17610908
686	Chumphon	Southern	2003	1.1.1	MDR-TB	ERR13666742	SRR17610907
687	Chumphon	Southern	2003	1.1.1	MDR-TB	ERR13666277	SRR17610905
688	Kalasin	Northeastern	2004	2.2.1	MDR-TB	ERR13666584	SRR17610904
689	Bangkok	Central	2004	1.1.1.1	MDR-TB	ERR13666650	SRR17610903
690	Bangkok	Central	2004	2.2.1	MDR-TB	ERR13666451	SRR17610901
691	Bangkok	Central	2004	1.2.2.2	HR-TB	ERR13666175	SRR17611023
692	Rayong	Central	2004	1.1.1	MDR-TB	ERR13666685	SRR17611022
693	Rayong	Central	2004	2.2.1	MDR-TB	ERR13666724	SRR17611021
694	Rayong	Central	2005	2.2.1	MDR-TB	ERR13666187	SRR17611020
695	Bangkok	Central	2005	2.2.1	MDR-TB	ERR13666095	SRR17611019
696	Chon Buri	Central	2005	2.2.1	MDR-TB	ERR13665979	SRR17611017
697	Yala	Southern	2005	2.2.1	MDR-TB	ERR13666303	SRR17611016
698	Suphan Buri	Central	2004	2.2.1	MDR-TB	ERR13666228	SRR17611015
699	Samut Prakan	Central	2004	2.2.1	MDR-TB	ERR13665878	SRR17611014
700	Buri Ram	Northeastern	2004	2.2.1	MDR-TB	ERR13666350	SRR17611013
701	Phitsanulok	Northern	2004	2.2.1	MDR-TB	ERR13666671	SRR17611012
702	Si Sa Ket	Northeastern	2005	2.2.1	MDR-TB	ERR13666791	SRR17611011
703	Nong Khai	Northeastern	2005	2.2.1	MDR-TB	ERR13666027	SRR17611010
704	rachuap Khiri Khar	Central	2005	2.1	MDR-TB	ERR13666145	SRR17611009
705	Phitsanulok	Northern	2005	2.1	MDR-TB	ERR13666347	SRR17611008
706	Sukhothai	Northern	2005	2.1	MDR-TB	ERR13666189	SRR17611006
707	Suphan Buri	Central	2006	2.2.1	MDR-TB	ERR13666609	SRR17611005
708	Rayong	Central	2006	2.2.1	Pre-XDR-TB	ERR13666018	SRR17611004
709	Prachin Buri	Central	2007	2.2.1	MDR-TB	ERR13666456	SRR17611003
710	Phitsanulok	Northern	2007	1.1.1	MDR-TB	ERR13665914	SRR17611002
711	Nong Khai	Northeastern	2007	2.1	MDR-TB	ERR13666138	SRR17611001
712	Phitsanulok	Northern	2008	2.2.1	MDR-TB	ERR13665910	SRR17611000
713	Saraburi	Central	2009	1.2.1.2.1	MDR-TB	ERR13665971	SRR17610874
714	Nong Khai	Northeastern	2010	1.1.1	MDR-TB	ERR13666096	SRR17610872
715	Nong Khai	Northeastern	2010	2.2.1	MDR-TB	ERR13666723	SRR17610871
716	Suphan Buri	Central	2011	4.2.2	MDR-TB	ERR13665945	SRR17610870
717	Nong Khai	Northeastern	2011	2.2.1.1	MDR-TB	ERR13666239	SRR17610869
718	Surat Thani	Southern	2011	2.2.1	MDR-TB	ERR13666649	SRR17610868
719	Suphan Buri	Central	2011	2.2.1	Other	ERR13666057	SRR17610867
720	Nong Khai	Northeastern	2011	1.1.1	MDR-TB	ERR13665897	SRR17610866
721	Nakhon Ratchasima	Northeastern	2007	2.2.1	MDR-TB	ERR13666483	SRR17610865
722	Ratchaburi	Central	2009	2.1	Pre-XDR-TB	ERR13666019	SRR17610864
723	Nakhon Ratchasima	Northeastern	2007	2.1	MDR-TB	ERR13666109	SRR17610863
724	Ratchaburi	Central	2009	2.1	MDR-TB	ERR13666402	SRR17610861
725	Mae Hong Son	Northern	2001	2.1	Pre-XDR-TB	ERR13666158	SRR17610860
726	Ratchaburi	Central	2012	2.1	Pre-XDR-TB	ERR13666449	SRR17610859

727	Chon Buri	Central	2012	2.1	MDR-TB	ERR13666842	SRR17610858
728	Ratchaburi	Central	2012	2.1	Pre-XDR-TB	ERR13666105	SRR17610857
729	Ratchaburi	Central	2009	2.1	Pre-XDR-TB	ERR13666043	SRR17610856
730	Buri Ram	Northeastern	2015	2.1	MDR-TB	ERR13666580	SRR17610855
731	Bangkok	Central	2015	2.1	MDR-TB	ERR13666225	SRR17610854
732	Nonthaburi	Central	2011	2.2.1.1	MDR-TB	ERR13665942	SRR17610853
733	Bangkok	Central	2012	2.1	MDR-TB	ERR13666351	SRR17611143
734	Phrae	Northern	2016	2.1	Pre-XDR-TB	ERR13666422	SRR17611141
735	Narathiwat	Southern	2013	2.1	MDR-TB	ERR13666190	SRR17611140
736	Kanchanaburi	Central	2014	2.1	MDR-TB	ERR13666120	SRR17611139
737	Phrae	Northern	2014	2.1	MDR-TB	ERR13666868	SRR17611138
738	Buri Ram	Northeastern	2015	2.2.1	RR-TB	ERR13666056	SRR17611137
739	Kanchanaburi	Central	2019	1.1.1	Sensitive	ERR13666373	SRR17611136
740	Kanchanaburi	Central	2019	1.1.1	Sensitive	ERR13666559	SRR17611135
741	Samut Prakan	Central	2003	2.2.1	MDR-TB	ERR13665883	SRR20855441
742	Bangkok	Central	2004	2.1	MDR-TB	ERR13666459	SRR20855440
743	Kanchanaburi	Central	2019	4.5	HR-TB	ERR13666317	SRR20855345
744	Kanchanaburi	Central	2019	1.1.1	HR-TB	ERR13665886	SRR20855395
745	Kanchanaburi	Central	2019	2.2.1	MDR-TB	ERR13666123	SRR20855253
746	Kanchanaburi	Central	2019	1.2.1.2.1	HR-TB	ERR13666726	SRR20855242
747	Kanchanaburi	Central	2019	2.2.1	HR-TB	ERR13665896	SRR20855231
748	Kanchanaburi	Central	2019	1.2.2.2	Other	ERR13666758	SRR20855439
749	Kanchanaburi	Central	2019	2.2.1.1	Sensitive	ERR13666298	SRR20855428
750	Kanchanaburi	Central	2019	1.1.1	HR-TB	ERR13666774	SRR20855417
751	Kanchanaburi	Central	2019	2.2.1	Sensitive	ERR13665887	SRR20855382
752	Kanchanaburi	Central	2019	1.1.1	Sensitive	ERR13666304	SRR20855371
753	Kanchanaburi	Central	2019	1.1.3.1	Sensitive	ERR13666360	SRR20855336
754	Kanchanaburi	Central	2019	2.2.1.2	Sensitive	ERR13666899	SRR20855325
755	Kanchanaburi	Central	2019	1.1.1	Sensitive	ERR13665998	SRR20855314
756	Kanchanaburi	Central	2019	1.2.1.2.1	Sensitive	ERR13666055	SRR20855303
757	Kanchanaburi	Central	2019	1.1.1	Sensitive	ERR13666592	SRR20855292
758	Kanchanaburi	Central	2019	2.2.1	Sensitive	ERR13665994	SRR20855280
759	Kanchanaburi	Central	2019	1.1.3.1	Sensitive	ERR13665995	SRR20855365
760	Kanchanaburi	Central	2019	2.2.1	Sensitive	ERR13666406	SRR20855354
761	Kanchanaburi	Central	2019	2.2.1	Sensitive	ERR13666850	SRR20855352
762	Kanchanaburi	Central	2019	1.2.1.2.1	Sensitive	ERR13666434	SRR20855351
763	Kanchanaburi	Central	2019	4.4.1.2	Sensitive	ERR13666767	SRR20855350
764	Kanchanaburi	Central	2019	4.1.1.1	Sensitive	ERR13665873	SRR20855349
765	Kanchanaburi	Central	2019	2.2.1	Other	ERR13665949	SRR20855348
766	Kanchanaburi	Central	2019	1.1.3.3	Sensitive	ERR13666173	SRR20855347
767	Kanchanaburi	Central	2019	2.2.1.1	Sensitive	ERR13666007	SRR20855346
768	Kanchanaburi	Central	2019	2.2.1	Sensitive	ERR13666199	SRR20855344
769	Kanchanaburi	Central	2019	1.2.1.2.1	Other	ERR13665936	SRR20855343
770	Kanchanaburi	Central	2019	4.4.2	Sensitive	ERR13666181	SRR20855414
771	Kanchanaburi	Central	2019	2.2.1	Sensitive	ERR13666835	SRR20855413
772	Kanchanaburi	Central	2019	2.2.1	Sensitive	ERR13665947	SRR20855412
773	Kanchanaburi	Central	2019	1.2.1.2.1	Sensitive	ERR13666395	SRR20855411
774	Kanchanaburi	Central	2019	1.1.1	Sensitive	ERR13666458	SRR20855410
775	Kanchanaburi	Central	2019	1.2.1.2.1	Sensitive	ERR13666150	SRR20855409
776	Kanchanaburi	Central	2019	1.1.1	Other	ERR13666219	SRR20855408
777	Kanchanaburi	Central	2019	2.2.1	Sensitive	ERR13666094	SRR20855407
778	Kanchanaburi	Central	2019	1.1.1.1	Sensitive	ERR13666311	SRR20855405

779	Kanchanaburi	Central	2019	4.5	Sensitive	ERR13666479	SRR20855404
780	Kanchanaburi	Central	2019	2.2.1	Sensitive	ERR13666577	SRR20855403
781	Kanchanaburi	Central	2019	1.1.3	Sensitive	ERR13666432	SRR20855402
782	Kanchanaburi	Central	2019	1.1.1	Sensitive	ERR13666556	SRR20855401
783	Kanchanaburi	Central	2019	1.2.1.2.1	Sensitive	ERR13666010	SRR20855400
784	Kanchanaburi	Central	2019	1.1.1	Sensitive	ERR13666267	SRR20855399
785	Kanchanaburi	Central	2019	2.2.1	Other	ERR13666041	SRR20855398
786	Kanchanaburi	Central	2019	1.1.1	Sensitive	ERR13666141	SRR20855397
787	Kanchanaburi	Central	2019	1.1.1	Sensitive	ERR13666356	SRR20855396
788	Kanchanaburi	Central	2019	1.1.1	Sensitive	ERR13666561	SRR20855394
789	Kanchanaburi	Central	2019	1.1.3.2	Sensitive	ERR13666811	SRR20855393
790	Kanchanaburi	Central	2019	2.2.1	Sensitive	ERR13666217	SRR20855391
791	Kanchanaburi	Central	2019	2.2.1	Sensitive	ERR13666343	SRR20855270
792	Kanchanaburi	Central	2019	2.2.1	Other	ERR13666889	SRR20855269
793	Kanchanaburi	Central	2019	2.2.1	Sensitive	ERR13666799	SRR20855268
794	Kanchanaburi	Central	2019	2.2.1	Other	ERR13666249	SRR20855267
795	Kanchanaburi	Central	2019	1.2.1.2.1	Other	ERR13666662	SRR20855266
796	Kanchanaburi	Central	2019	1.1.1	Sensitive	ERR13666238	SRR20855265
797	Kanchanaburi	Central	2019	1.1.2	Sensitive	ERR13666898	SRR20855263
798	Kanchanaburi	Central	2019	1.2.1.2.1	Sensitive	ERR13666281	SRR20855262
799	Kanchanaburi	Central	2020	1.1.1	Other	ERR13666346	SRR20855260
800	Kanchanaburi	Central	2020	1.2.1.2.1	Sensitive	ERR13666905	SRR20855259
801	Kanchanaburi	Central	2020	2.2.1	Sensitive	ERR13666288	SRR20855258
802	Kanchanaburi	Central	2020	1.2.1.2.1	Sensitive	ERR13666211	SRR20855257
803	Kanchanaburi	Central	2020	1.1.1	Sensitive	ERR13666587	SRR20855256
804	Kanchanaburi	Central	2020	1.1.2	Sensitive	ERR13666460	SRR20855255
805	Kanchanaburi	Central	2020	2.2.1	Other	ERR13665888	SRR20855254
806	Kanchanaburi	Central	2020	1.1.1	Sensitive	ERR13666423	SRR20855252
807	Kanchanaburi	Central	2020	1.1.2	Sensitive	ERR13666282	SRR20855251
808	Kanchanaburi	Central	2020	1.1.3	Sensitive	ERR13666179	SRR20855250
809	Kanchanaburi	Central	2020	1.1.1	HR-TB	ERR13666386	SRR20855248
810	Kanchanaburi	Central	2020	2.2.1	Sensitive	ERR13666081	SRR20855247
811	Kanchanaburi	Central	2020	1.1.1	Sensitive	ERR13666783	SRR20855245
812	Kanchanaburi	Central	2020	2.2.1.1	Sensitive	ERR13666165	SRR20855241
813	Kanchanaburi	Central	2020	1.1.1	Sensitive	ERR13666022	SRR20855239
814	Kanchanaburi	Central	2020	1.1.1.1	Sensitive	ERR13665916	SRR20855238
815	Kanchanaburi	Central	2020	2.2.1	HR-TB	ERR13666060	SRR20855237
816	Kanchanaburi	Central	2020	1.1.1	Sensitive	ERR13666364	SRR20855236
817	Kanchanaburi	Central	2020	1.2.1.2.1	Sensitive	ERR13665899	SRR20855235
818	Kanchanaburi	Central	2020	2.2.1	Sensitive	ERR13666579	SRR20855234
819	Kanchanaburi	Central	2020	1.2.1.2	Sensitive	ERR13666884	SRR20855233
820	Kanchanaburi	Central	2020	1.1.3.1	Sensitive	ERR13665915	SRR20855232
821	Kanchanaburi	Central	2020	1.2.1.2	Sensitive	ERR13666244	SRR20855230
822	Kanchanaburi	Central	2020	1.1.1	Sensitive	ERR13666768	SRR20855229
823	Kanchanaburi	Central	2020	1.1.1	Sensitive	ERR13666164	SRR20855228
824	Kanchanaburi	Central	2017	2.2.1	Sensitive	ERR13666773	SRR20855227
825	Kanchanaburi	Central	2017	2.2.1	Sensitive	ERR13666543	SRR20855226
826	Kanchanaburi	Central	2017	2.2.1.2	Sensitive	ERR13666047	SRR20855225
827	Kanchanaburi	Central	2017	1.2.1.2.1	HR-TB	ERR13666836	SRR20855224
828	Kanchanaburi	Central	2017	2.2.2	Sensitive	ERR13666696	SRR20855223
829	Kanchanaburi	Central	2017	1.1.1	Sensitive	ERR13666683	SRR20855222
830	Kanchanaburi	Central	2017	1.1.1	Sensitive	ERR13666675	SRR20855221

831	Kanchanaburi	Central	2017	2.2.1	Sensitive	ERR13666888	SRR20855438
832	Kanchanaburi	Central	2017	4.5	HR-TB	ERR13666554	SRR20855437
833	Kanchanaburi	Central	2017	1.2.1.2	Sensitive	ERR13665904	SRR20855436
834	Kanchanaburi	Central	2017	2.2.1	Sensitive	ERR13666312	SRR20855435
835	Kanchanaburi	Central	2020	1.1.1	Sensitive	ERR13665898	SRR20855434
836	Kanchanaburi	Central	2017	4.5	Sensitive	ERR13666711	SRR20855433
837	Kanchanaburi	Central	2017	2.2.1	RR-TB	ERR13666409	SRR20855432
838	Kanchanaburi	Central	2017	1.1.3	Sensitive	ERR13665927	SRR20855431
839	Kanchanaburi	Central	2017	2.2.1	MDR-TB	ERR13666784	SRR20855430
840	Kanchanaburi	Central	2017	2.2.1	Sensitive	ERR13666496	SRR20855429
841	Kanchanaburi	Central	2017	1.2.1.2.1	Sensitive	ERR13666153	SRR20855427
842	Kanchanaburi	Central	2017	2.1	Sensitive	ERR13666522	SRR20855426
843	Kanchanaburi	Central	2017	1.1.2	Sensitive	ERR13666567	SRR20855425
844	Kanchanaburi	Central	2017	1.2.1.2	Sensitive	ERR13666494	SRR20855424
845	Kanchanaburi	Central	2017	4.5	Pre-XDR-TB	ERR13666721	SRR20855423
846	Kanchanaburi	Central	2017	2.2.1	Sensitive	ERR13666677	SRR20855422
847	Kanchanaburi	Central	2017	1.1.1	Sensitive	ERR13666867	SRR20855421
848	Kanchanaburi	Central	2017	1.1.3	Sensitive	ERR13666143	SRR20855420
849	Kanchanaburi	Central	2017	1.1.1	Sensitive	ERR13666188	SRR20855419
850	Kanchanaburi	Central	2017	1.1.1	Sensitive	ERR13665913	SRR20855418
851	Kanchanaburi	Central	2017	4.1.2	Other	ERR13666824	SRR20855416
852	Kanchanaburi	Central	2017	1.1.3	Sensitive	ERR13666465	SRR20855415
853	Kanchanaburi	Central	2017	1.2.1.2.1	Sensitive	ERR13666694	SRR20855390
854	Kanchanaburi	Central	2017	2.2.1	HR-TB	ERR13666342	SRR20855389
855	Kanchanaburi	Central	2017	2.1	HR-TB	ERR13666576	SRR20855387
856	Kanchanaburi	Central	2017	1.1.1	Sensitive	ERR13666521	SRR20855386
857	Kanchanaburi	Central	2017	2.2.1	Sensitive	ERR13665890	SRR20855385
858	Kanchanaburi	Central	2017	4.5	HR-TB	ERR13666848	SRR20855384
859	Kanchanaburi	Central	2017	2.2.1	Sensitive	ERR13666575	SRR20855383
860	Kanchanaburi	Central	2017	1.2.1.2.1	Sensitive	ERR13666102	SRR20855381
861	Kanchanaburi	Central	2017	1.1.1	Sensitive	ERR13666241	SRR20855380
862	Kanchanaburi	Central	2017	1.1.3.1	Sensitive	ERR13666517	SRR20855379
863	Kanchanaburi	Central	2017	1.2.1.2.1	Sensitive	ERR13665974	SRR20855378
864	Kanchanaburi	Central	2017	2.2.1.1	Sensitive	ERR13666103	SRR20855377
865	Kanchanaburi	Central	2017	1.2.1.2	Sensitive	ERR13666578	SRR20855376
866	Kanchanaburi	Central	2017	1.1.1	Sensitive	ERR13665980	SRR20855374
867	Kanchanaburi	Central	2017	1.1.1	Sensitive	ERR13666730	SRR20855373
868	Kanchanaburi	Central	2017	2.2.1	Sensitive	ERR13666852	SRR20855372
869	Kanchanaburi	Central	2017	2.2.1	Sensitive	ERR13666365	SRR20855370
870	Kanchanaburi	Central	2017	1.1.1	Sensitive	ERR13666450	SRR20855369
871	Kanchanaburi	Central	2017	1.1.1	Sensitive	ERR13666385	SRR20855368
872	Kanchanaburi	Central	2017	1.1.1	Sensitive	ERR13666765	SRR20855367
873	Kanchanaburi	Central	2017	2.2.1	Sensitive	ERR13666005	SRR20855342
874	Kanchanaburi	Central	2017	1.2.1.2.1	Other	ERR13666471	SRR20855341
875	Kanchanaburi	Central	2017	2.2.1	Sensitive	ERR13666802	SRR20855340
876	Kanchanaburi	Central	2017	4.5	Sensitive	ERR13666391	SRR20855339
877	Kanchanaburi	Central	2017	2.1	HR-TB	ERR13666550	SRR20855338
878	Kanchanaburi	Central	2017	2.2.1	Sensitive	ERR13666604	SRR20855337
879	Kanchanaburi	Central	2017	1.1.3	Sensitive	ERR13666206	SRR20855335
880	Kanchanaburi	Central	2018	1.1.1	Sensitive	ERR13666732	SRR20855334
881	Kanchanaburi	Central	2018	1.2.2.2	Other	ERR13666524	SRR20855333
882	Kanchanaburi	Central	2018	1.1.3.3	Sensitive	ERR13666864	SRR20855332

883	Kanchanaburi	Central	2018	1.2.1.2.1	HR-TB	ERR13666603	SRR20855331
884	Kanchanaburi	Central	2018	1.1.1	Sensitive	ERR13666749	SRR20855330
885	Kanchanaburi	Central	2018	1.1.3.1	Sensitive	ERR13666693	SRR20855329
886	Kanchanaburi	Central	2018	4.1.1.1	Sensitive	ERR13666439	SRR20855328
887	Kanchanaburi	Central	2018	2.2.1.1	Sensitive	ERR13666036	SRR20855327
888	Kanchanaburi	Central	2018	2.2.1	Sensitive	ERR13666885	SRR20855326
889	Kanchanaburi	Central	2018	1.2.1.2	Sensitive	ERR13666305	SRR20855324
890	Kanchanaburi	Central	2018	2.2.1	Other	ERR13666418	SRR20855323
891	Kanchanaburi	Central	2018	1.1.3.1	HR-TB	ERR13666127	SRR20855322
892	Kanchanaburi	Central	2018	2.2.1	Sensitive	ERR13666582	SRR20855321
893	Kanchanaburi	Central	2018	1.2.1.2.1	Sensitive	ERR13665954	SRR20855320
894	Kanchanaburi	Central	2018	1.2.1.2	Sensitive	ERR13666246	SRR20855319
895	Kanchanaburi	Central	2018	1.2.2.2	HR-TB	ERR13666159	SRR20855318
896	Kanchanaburi	Central	2018	4.5	Sensitive	ERR13666812	SRR20855317
897	Kanchanaburi	Central	2018	2.2.1	Other	ERR13666895	SRR20855316
898	Kanchanaburi	Central	2018	1.1.1	Other	ERR13666045	SRR20855315
899	Kanchanaburi	Central	2018	1.1.3.1	Sensitive	ERR13666875	SRR20855313
900	Kanchanaburi	Central	2018	1.2.1.2	Sensitive	ERR13666400	SRR20855312
901	Kanchanaburi	Central	2018	2.2.1	HR-TB	ERR13666855	SRR20855311
902	Kanchanaburi	Central	2018	1.1.1	Sensitive	ERR13666226	SRR20855310
903	Kanchanaburi	Central	2018	1.2.1.2	Sensitive	ERR13666757	SRR20855309
904	Kanchanaburi	Central	2018	2.2.1	Sensitive	ERR13666656	SRR20855308
905	Kanchanaburi	Central	2018	4.1.1.1	Sensitive	ERR13666608	SRR20855307
906	Kanchanaburi	Central	2018	1.1.1	Sensitive	ERR13666160	SRR20855306
907	Kanchanaburi	Central	2018	4.5	Sensitive	ERR13666630	SRR20855305
908	Kanchanaburi	Central	2018	1.1.1	Sensitive	ERR13666807	SRR20855304
909	Kanchanaburi	Central	2018	4.4.2	Sensitive	ERR13666718	SRR20855302
910	Kanchanaburi	Central	2018	1.2.2.1	HR-TB	ERR13666737	SRR20855301
911	Kanchanaburi	Central	2018	1.2.1.2	Sensitive	ERR13666502	SRR20855300
912	Kanchanaburi	Central	2018	1.2.1.2.1	Sensitive	ERR13666871	SRR20855299
913	Kanchanaburi	Central	2018	1.1.1	HR-TB	ERR13666280	SRR20855298
914	Kanchanaburi	Central	2018	1.1.1	Sensitive	ERR13666755	SRR20855297
915	Kanchanaburi	Central	2018	2.1	RR-TB	ERR13666759	SRR20855296
916	Kanchanaburi	Central	2018	2.2.1	HR-TB	ERR13666633	SRR20855295
917	Kanchanaburi	Central	2018	1.1.1	Sensitive	ERR13666230	SRR20855294
918	Kanchanaburi	Central	2018	2.2.1.1	Sensitive	ERR13665934	SRR20855293
919	Kanchanaburi	Central	2018	1.1.1	Sensitive	ERR13666731	SRR20855291
920	Kanchanaburi	Central	2018	1.1.1.1	HR-TB	ERR13666046	SRR20855290
921	Kanchanaburi	Central	2018	1.2.1.2	Sensitive	ERR13666003	SRR20855289
922	Kanchanaburi	Central	2018	1.2.1.2.1	Sensitive	ERR13666024	SRR20855288
923	Kanchanaburi	Central	2018	4.5	Sensitive	ERR13666384	SRR20855287
924	Kanchanaburi	Central	2018	4.5	Sensitive	ERR13666806	SRR20855286
925	Kanchanaburi	Central	2019	2.1	Sensitive	ERR13666481	SRR20855285
926	Kanchanaburi	Central	2019	2.1	HR-TB	ERR13666172	SRR20855284
927	Kanchanaburi	Central	2019	2.2.1	Sensitive	ERR13666599	SRR20855283
928	Kanchanaburi	Central	2019	1.2.1.2.1	Sensitive	ERR13666148	SRR20855282
929	Kanchanaburi	Central	2019	2.1	Sensitive	ERR13666454	SRR20855279
930	Kanchanaburi	Central	2020	1.2.1.2.1	Sensitive	ERR13666436	SRR20855278
931	Kanchanaburi	Central	2020	2.2.1	Sensitive	ERR13666854	SRR20855277
932	Kanchanaburi	Central	2020	2.1	Sensitive	ERR13666327	SRR20855276
933	Kanchanaburi	Central	2020	1.1.1	Sensitive	ERR13666097	SRR20855275
934	Kanchanaburi	Central	2020	4.4.2	Sensitive	ERR13666574	SRR20855274

935	Kanchanaburi	Central	2020	1.2.1.2.1	Other	ERR13666322	SRR20855272
936	Kanchanaburi	Central	2020	1.2.2.2	Sensitive	ERR13666152	SRR20855271
937	Kanchanaburi	Central	2020	1.1.1	HR-TB	ERR13666902	SRR20855366
938	Kanchanaburi	Central	2020	1.2.1.2.1	Sensitive	ERR13665960	SRR20855364
939	Kanchanaburi	Central	2020	2.2.1	Sensitive	ERR13666821	SRR20855363
940	Kanchanaburi	Central	2020	1.1.3.3	Sensitive	ERR13666248	SRR20855362
941	Kanchanaburi	Central	2020	4.5	Sensitive	ERR13666369	SRR20855361
942	Kanchanaburi	Central	2020	4.5	Other	ERR13665877	SRR20855360
943	Kanchanaburi	Central	2020	1.1.1	Sensitive	ERR13666796	SRR20855359
944	Kanchanaburi	Central	2020	1.1.1.1	Sensitive	ERR13666380	SRR20855357
945	Kanchanaburi	Central	2020	1.1.1	Sensitive	ERR13666077	SRR20855356
946	Kanchanaburi	Central	2020	1.1.1	HR-TB	ERR13666265	SRR20855355
947	Kanchanaburi	Central	2020	1.1.1	HR-TB	ERR13666324	SRR20855353
948	Kanchanaburi	Central	2004	2.2.1	MDR-TB	ERR13666371	
949	Kanchanaburi	Central	2004	2.2.1	MDR-TB	ERR13666754	
950	Kanchanaburi	Central	2004	2.2.1	MDR-TB	ERR13666477	
951	Kanchanaburi	Central	2009	2.2.1	MDR-TB	ERR13665933	
952	Kanchanaburi	Central	2004	2.2.1	MDR-TB	ERR13666124	
953	Kanchanaburi	Central	2004	2.2.1	MDR-TB	ERR13666858	
954	Kanchanaburi	Central	2004	2.2.1	MDR-TB	ERR13665996	
955	Kanchanaburi	Central	2005	2.2.1	MDR-TB	ERR13666798	
956	Kanchanaburi	Central	2005	2.2.1	MDR-TB	ERR13665937	
957	Kanchanaburi	Central	2006	2.2.1	MDR-TB	ERR13666901	
958	Kanchanaburi	Central	2007	2.2.1	MDR-TB	ERR13665978	
959	Kanchanaburi	Central	2007	2.2.1	MDR-TB	ERR13666878	
960	Kanchanaburi	Central	2007	2.2.1	MDR-TB	ERR13666642	
961	Kanchanaburi	Central	2007	2.2.1	MDR-TB	ERR13666619	
962	Kanchanaburi	Central	2007	2.2.1	MDR-TB	ERR13666063	
963	Kanchanaburi	Central	2007	2.2.1	MDR-TB	ERR13666691	
964	Kanchanaburi	Central	2007	2.2.1	MDR-TB	ERR13666602	
965	Kanchanaburi	Central	2007	2.2.1	MDR-TB	ERR13666476	
966	Kanchanaburi	Central	2008	2.2.1	MDR-TB	ERR13666427	
967	Kanchanaburi	Central	2008	2.1	Pre-XDR-TB	ERR13666330	
968	Kanchanaburi	Central	2008	2.2.1	MDR-TB	ERR13666638	
969	Kanchanaburi	Central	2008	2.2.1	MDR-TB	ERR13666780	
970	Kanchanaburi	Central	2008	2.2.1	MDR-TB	ERR13666250	
971	Kanchanaburi	Central	2008	2.2.1	MDR-TB	ERR13666487	
972	Kanchanaburi	Central	2009	2.2.1	MDR-TB	ERR13666098	
973	Kanchanaburi	Central	2009	2.2.1	MDR-TB	ERR13666108	
974	Kanchanaburi	Central	2009	2.2.1	Pre-XDR-TB	ERR13666092	
975	Kanchanaburi	Central	2009	2.2.1	MDR-TB	ERR13666272	
976	Kanchanaburi	Central	2009	2.2.1	MDR-TB	ERR13666117	
977	Kanchanaburi	Central	2007	2.2.1	MDR-TB	ERR13666682	
978	Kanchanaburi	Central	2007	2.2.1	Other	ERR13666591	
979	Kanchanaburi	Central	2009	1.1.3.1	Sensitive	ERR13666338	
980	Kanchanaburi	Central	2006	4.2.2	MDR-TB	ERR13666069	
981	Kanchanaburi	Central	2009	2.2.1	MDR-TB	ERR13666853	
982	Kanchanaburi	Central	2011	2.2.1.1	MDR-TB	ERR13666607	
983	Kanchanaburi	Central	2011	2.2.1	MDR-TB	ERR13666542	
984	Kanchanaburi	Central	2011	2.2.1	MDR-TB	ERR13666000	
985	Kanchanaburi	Central	2011	1.1.2	MDR-TB	ERR13666601	
986	Kanchanaburi	Central	2011	1.1.1	MDR-TB	ERR13666525	

987	Kanchanaburi	Central	2006	2.2.1	MDR-TB	ERR13666245
988	Kanchanaburi	Central	2006	4.2.2	Sensitive	ERR13666366
989	Kanchanaburi	Central	2013	2.2.1	MDR-TB	ERR13665929
990	Kanchanaburi	Central	2013	2.2.1	MDR-TB	ERR13666841
991	Kanchanaburi	Central	2013	2.2.1	MDR-TB	ERR13666571
992	Kanchanaburi	Central	2013	2.2.1.1	MDR-TB	ERR13666417
993	Kanchanaburi	Central	2013	2.2.1	MDR-TB	ERR13665925
994	Kanchanaburi	Central	2013	2.2.1	MDR-TB	ERR13666349
995	Kanchanaburi	Central	2013	2.2.1	MDR-TB	ERR13666194
996	Kanchanaburi	Central	2013	2.2.1.1	MDR-TB	ERR13666020
997	Kanchanaburi	Central	2013	2.2.1	MDR-TB	ERR13666111
998	Kanchanaburi	Central	2013	2.2.1	MDR-TB	ERR13666254
999	Kanchanaburi	Central	2014	2.2.1	MDR-TB	ERR13666800
1000	Kanchanaburi	Central	2014	2.2.1	MDR-TB	ERR13666627
1001	Kanchanaburi	Central	2014	2.2.1	Pre-XDR-TB	ERR13666437
1002	Kanchanaburi	Central	2014	2.2.1	MDR-TB	ERR13665876
1003	Kanchanaburi	Central	2014	2.2.1	MDR-TB	ERR13666017
1004	Kanchanaburi	Central	2014	2.2.1	MDR-TB	ERR13665906
1005	Kanchanaburi	Central	2014	2.2.1	MDR-TB	ERR13666771
1006	Kanchanaburi	Central	2014	2.2.1	MDR-TB	ERR13666531
1007	Kanchanaburi	Central	2014	2.2.1	MDR-TB	ERR13666819
1008	Kanchanaburi	Central	2014	2.2.1	MDR-TB	ERR13666803
1009	Kanchanaburi	Central	2014	2.2.1	MDR-TB	ERR13666679
1010	Kanchanaburi	Central	2014	2.2.1	MDR-TB	ERR13666029
1011	Kanchanaburi	Central	2014	2.2.1	MDR-TB	ERR13666091
1012	Kanchanaburi	Central	2006	2.2.1	MDR-TB	ERR13666639
1013	Bangkok	Central	2004	1.1.1	MDR-TB	ERR13666739
1014	Bangkok	Central	2004	2.2.1	Pre-XDR-TB	ERR13666212
1015	Bangkok	Central	2016	2.2.1	MDR-TB	ERR13666104
1016	Bangkok	Central	2015	2.2.1	Pre-XDR-TB	ERR13666174
1017	Bangkok	Central	2013	2.2.1	Pre-XDR-TB	ERR13666686
1018	Bangkok	Central	2014	2.2.1	Pre-XDR-TB	ERR13666078
1019	Bangkok	Central	2014	2.2.1	Pre-XDR-TB	ERR13665958
1020	Bangkok	Central	2014	2.2.1	Pre-XDR-TB	ERR13666412
1021	Bangkok	Central	2014	2.2.1	Pre-XDR-TB	ERR13666446
1022	Bangkok	Central	2014	2.2.1	MDR-TB	ERR13666781
1023	Bangkok	Central	2015	2.2.1	Pre-XDR-TB	ERR13666184
1024	Bangkok	Central	2015	2.2.1	Pre-XDR-TB	ERR13666054
1025	Bangkok	Central	2015	2.2.1	Pre-XDR-TB	ERR13666539
1026	Bangkok	Central	2015	2.2.1	Pre-XDR-TB	ERR13666593
1027	Bangkok	Central	2015	2.2.1	Pre-XDR-TB	ERR13666085
1028	Bangkok	Central	2015	2.2.1	Pre-XDR-TB	ERR13666536
1029	Bangkok	Central	2015	2.2.1	Pre-XDR-TB	ERR13666715
1030	Bangkok	Central	2015	2.2.2	Pre-XDR-TB	ERR13666032
1031	Bangkok	Central	2015	2.2.1	Pre-XDR-TB	ERR13666488
1032	Bangkok	Central	2002	2.2.1	MDR-TB	ERR13666621
1033	Bangkok	Central	2005	2.2.1	MDR-TB	ERR13666654
1034	Ratchaburi	Central	2002	2.2.1	MDR-TB	ERR13666833
1035	Ratchaburi	Central	2009	2.1	MDR-TB	ERR13666562
1036	Ratchaburi	Central	2011	2.2.1	MDR-TB	ERR13666452
1037	Ratchaburi	Central	2011	2.2.1	MDR-TB	ERR13666692
1038	Ratchaburi	Central	2011	2.2.1	MDR-TB	ERR13666540

1039	Ratchaburi	Central	2012	2.1	MDR-TB	ERR13666516
1040	Ratchaburi	Central	2006	4.2.2	MDR-TB	ERR13666876
1041	Chanthaburi	Central	2014	1.1.1	MDR-TB	SRR11662127
1042	Sing Buri	Central	2014	2.2.1	MDR-TB	SRR11662128
1043	Prachin Buri	Central	2014	2.2.1	Pre-XDR-TB	SRR11662129
1044	Prachin Buri	Central	2016	2.2.1	MDR-TB	SRR11662130
1045	Songkhla	Southern	2014	2.2.1.1	MDR-TB	SRR11662131
1046	Bangkok	Central	2014	2.2.1	MDR-TB	SRR11662132
1047	Bangkok	Central	2016	1.1.1	MDR-TB	SRR11662133
1048	Bangkok	Central	2016	2.2.1	MDR-TB	SRR11662134
1049	Tak	Northern	2014	2.2.1	MDR-TB	SRR11662135
1050	Bangkok	Central	2016	2.2.1.1	Pre-XDR-TB	SRR11662136
1051	Tak	Northern	2014	2.2.1.1	Pre-XDR-TB	SRR11662137
1052	Chon Buri	Central	2015	2.2.1	Pre-XDR-TB	SRR11662138
1053	Phitsanulok	Northern	2016	2.2.1	Pre-XDR-TB	SRR11662139
1054	Tak	Northern	2014	2.2.1.1	Pre-XDR-TB	SRR11662140
1055	Bangkok	Central	2017	2.2.1	MDR-TB	SRR11662141
1056	Prachin Buri	Central	2016	2.2.1	Pre-XDR-TB	SRR11662142
1057	Nakhon Sawan	Northern	2014	2.2.1	MDR-TB	SRR11662143
1058	Ratchaburi	Central	2014	2.2.1	MDR-TB	SRR11662144
1059	Samut Sakhon	Central	2014	2.2.1	MDR-TB	SRR11662145
1060	Kanchanaburi	Central	2015	2.2.1	Pre-XDR-TB	SRR11662146
1061	Kanchanaburi	Central	2014	2.2.1	MDR-TB	SRR11662147
1062	Nakhon Pathom	Central	2015	2.2.1	MDR-TB	SRR11662148
1063	Kanchanaburi	Central	2014	2.2.1	MDR-TB	SRR11662149
1064	Kanchanaburi	Central	2014	2.2.1	MDR-TB	SRR11662150
1065	Bangkok	Central	2015	2.2.1.1	Other	SRR11662151
1066	Bangkok	Central	2017	2.2.1	Pre-XDR-TB	SRR11662152
1067	Chanthaburi	Central	2016	2.2.1	MDR-TB	SRR11662153
1068	Bangkok	Central	2017	2.2.1	MDR-TB	SRR11662154
1069	Bangkok	Central	2017	2.2.1	MDR-TB	SRR11662155
1070	Bangkok	Central	2014	2.2.1	MDR-TB	SRR11662156
1071	Bangkok	Central	2015	2.2.1	MDR-TB	SRR11662157
1072	Bangkok	Central	2017	2.2.1	MDR-TB	SRR11662158
1073	Chon Buri	Central	2014	2.2.1	MDR-TB	SRR11662159
1074	Chon Buri	Central	2014	2.2.1	MDR-TB	SRR11662161
1075	Trat	Central	2016	2.1	Pre-XDR-TB	SRR11662162
1076	Trat	Central	2016	1.1.1	MDR-TB	SRR11662163
1077	Chon Buri	Central	2016	1.1.2	MDR-TB	SRR11662164
1078	Bangkok	Central	2017	2.2.1	MDR-TB	SRR11662165
1079	'rachuap Khiri Khan	Central	2016	2.2.1	MDR-TB	SRR11662166
1080	Phuket	Southern	2016	1.1.1	MDR-TB	SRR11662167
1081	Samut Sakhon	Central	2015	2.2.1	MDR-TB	SRR11662168
1082	Kamphaeng Phet	Northern	2014	2.2.1	Pre-XDR-TB	SRR11662169
1083	Ubon Ratchathani	Northeastern	2014	1.1.1	MDR-TB	SRR11662170
1084	Bangkok	Central	2015	4.4.2	MDR-TB	SRR11662171
1085	Si Sa Ket	Northeastern	2014	2.2.1	MDR-TB	SRR11662172
1086	Nakhon Phanom	Northeastern	2014	2.2.1	MDR-TB	SRR11662173
1087	Nonthaburi	Central	2017	2.2.1	MDR-TB	SRR11662174
1088	Bangkok	Central	2017	2.2.1	MDR-TB	SRR11662175
1089	Bangkok	Central	2016	2.2.1	MDR-TB	SRR11662176
1090	Trat	Central	2014	2.2.1	MDR-TB	SRR11662177

1091	Chon Buri	Central	2014	2.2.1	MDR-TB	SRR11662178
1092	akhon Si Thammara	Southern	2014	2.2.1	MDR-TB	SRR11662179
1093	akhon Si Thammara	Southern	2014	1.1.3.3	MDR-TB	SRR11662180
1094	Surat Thani	Southern	2014	2.2.1	MDR-TB	SRR11662181
1095	Sukhothai	Northern	2014	2.1	MDR-TB	SRR11662182
1096	Chiang Mai	Northern	2016	2.2.1	MDR-TB	SRR11662183
1097	Narathiwat	Southern	2016	2.2.1	MDR-TB	SRR11662184
1098	Ubon Ratchathani	Northeastern	2016	2.2.1	MDR-TB	SRR11662185
1099	Kanchanaburi	Central	2016	2.2.1	MDR-TB	SRR11662186
1100	Ubon Ratchathani	Northeastern	2016	2.2.1	Pre-XDR-TB	SRR11662187
1101	Phetchaburi	Central	2014	2.2.1	MDR-TB	SRR11662188
1102	Kanchanaburi	Central	2014	2.2.1	MDR-TB	SRR11662189
1103	Kanchanaburi	Central	2014	2.2.1	MDR-TB	SRR11662190
1104	Chon Buri	Central	2014	2.2.1	MDR-TB	SRR11662191
1105	Chai Nat	Central	2015	2.2.1	MDR-TB	SRR11662192
1106	Ratchaburi	Central	2017	2.2.1.1	MDR-TB	SRR11662193
1107	Nakhon Pathom	Central	2017	2.2.1.1	MDR-TB	SRR11662194
1108	Bangkok	Central	2017	2.2.1.1	MDR-TB	SRR11662195
1109	Buri Ram	Northeastern	2016	2.2.1	MDR-TB	SRR11662196
1110	Kanchanaburi	Central	2016	1.1.1	MDR-TB	SRR11662197
1111	Surin	Northeastern	2016	2.2.1	MDR-TB	SRR11662198
1112	Bangkok	Central	2015	1.1.1	MDR-TB	SRR11662200
1113	Sukhothai	Northern	2015	2.2.1	MDR-TB	SRR11662201
1114	Prachin Buri	Central	2017	1.1.1	MDR-TB	SRR11662202
1115	Chaiyaphum	Northeastern	2015	2.2.1	MDR-TB	SRR11662203
1116	Nakhon Ratchasima	Northeastern	2015	2.2.1	MDR-TB	SRR11662204
1117	Prachin Buri	Central	2015	2.2.1	MDR-TB	SRR11662205
1118	Pathum Thani	Central	2015	2.2.1	MDR-TB	SRR11662206
1119	Chon Buri	Central	2015	2.2.1	MDR-TB	SRR11662207
1120	Tak	Northern	2015	2.2.1	MDR-TB	SRR11662208
1121	Kamphaeng Phet	Northern	2015	2.2.1	MDR-TB	SRR11662209
1122	Songkhla	Southern	2015	2.2.1	MDR-TB	SRR11662210
1123	Ubon Ratchathani	Northeastern	2015	1.1.1	MDR-TB	SRR11662211
1124	Phuket	Southern	2015	2.2.1.1	MDR-TB	SRR11662212
1125	Amnat Charoen	Northeastern	2016	2.2.1	Pre-XDR-TB	SRR11662213
1126	Chaiyaphum	Northeastern	2015	2.2.1	MDR-TB	SRR11662214
1127	Chon Buri	Central	2015	1.1.1	MDR-TB	SRR11662215
1128	Chon Buri	Central	2015	2.2.1	MDR-TB	SRR11662216
1129	Sakon Nakhon	Northeastern	2015	1.1.1	MDR-TB	SRR11662217
1130	Trang	Southern	2015	1.1.1	MDR-TB	SRR11662218
1131	Surat Thani	Southern	2015	2.2.1	MDR-TB	SRR11662219
1132	akhon Si Thammara	Southern	2017	4.5	MDR-TB	SRR11662220
1133	Bangkok	Central	2015	2.2.1	MDR-TB	SRR11662221
1134	Bangkok	Central	2014	4.2.2	MDR-TB	SRR11662222
1135	Trat	Central	2014	2.1	MDR-TB	SRR11662223
1136	Yala	Southern	2016	2.2.1.1	MDR-TB	SRR11662224
1137	Trang	Southern	2017	1.1.3.3	MDR-TB	SRR11662225
1138	Nong Bua Lamphu	Northeastern	2016	2.2.1	Pre-XDR-TB	SRR11662226
1139	Udon Thani	Northeastern	2016	2.2.1	MDR-TB	SRR11662227
1140	Kalasin	Northeastern	2016	2.2.1	MDR-TB	SRR11662228
1141	Khon Kaen	Northeastern	2016	4.5	MDR-TB	SRR11662229
1142	Khon Kaen	Northeastern	2016	2.2.1.1	MDR-TB	SRR11662231

1143	Nong Khai	Northeastern	2016	2.2.1	MDR-TB	SRR11662232
1144	Khon Kaen	Northeastern	2016	2.2.1	MDR-TB	SRR11662233
1145	Khon Kaen	Northeastern	2016	2.2.1	MDR-TB	SRR11662234
1146	Suphan Buri	Central	2017	2.2.1	MDR-TB	SRR11662235
1147	'rachuap Khiri Khar	Central	2017	4.2.2	MDR-TB	SRR11662236
1148	Phetchaburi	Central	2017	2.2.1	MDR-TB	SRR11662237
1149	Kanchanaburi	Central	2017	2.2.1	MDR-TB	SRR11662238
1150	'rachuap Khiri Khar	Central	2017	2.2.1	MDR-TB	SRR11662239
1151	Samut Sakhon	Central	2017	2.2.1	MDR-TB	SRR11662240
1152	Kanchanaburi	Central	2016	2.2.1	MDR-TB	SRR11662241
1153	Kanchanaburi	Central	2017	2.2.1	MDR-TB	SRR11662242
1154	Kanchanaburi	Central	2017	2.2.1	MDR-TB	SRR11662243
1155	Kanchanaburi	Central	2017	2.2.1	Pre-XDR-TB	SRR11662244
1156	Samut Sakhon	Central	2017	2.2.1	MDR-TB	SRR11662245
1157	Kanchanaburi	Central	2017	2.2.1	Pre-XDR-TB	SRR11662246
1158	Suphan Buri	Central	2015	2.2.1	MDR-TB	SRR11662247
1159	Chon Buri	Central	2017	2.2.1	MDR-TB	SRR11662248
1160	Khon Kaen	Northeastern	2014	1.1.3.3	MDR-TB	SRR11662249
1161	Khon Kaen	Northeastern	2014	2.2.1	Pre-XDR-TB	SRR11662250
1162	Songkhla	Southern	2017	2.2.1	MDR-TB	SRR11662251
1163	Bangkok	Central	2016	1.1.1.1	MDR-TB	SRR11662252
1164	Bangkok	Central	2016	2.2.2	Pre-XDR-TB	SRR11662253
1165	Nakhon Sawan	Northern	2016	2.2.1	MDR-TB	SRR11662254
1166	Kamphaeng Phet	Northern	2014	2.2.1	MDR-TB	SRR11662255
1167	Chai Nat	Central	2014	2.2.1	MDR-TB	SRR11662256
1168	Nakhon Sawan	Northern	2014	2.2.1	MDR-TB	SRR11662257
1169	Kanchanaburi	Central	2017	4.5	HR-TB	SRR11662258
1170	Ratchaburi	Central	2017	2.2.1	Pre-XDR-TB	SRR11662259
1171	Phetchabun	Northern	2014	2.2.1	MDR-TB	SRR11662260
1172	Samut Sakhon	Central	2017	2.2.1	MDR-TB	SRR11662261
1173	Phitsanulok	Northern	2014	1.2.1.2.1	MDR-TB	SRR11662262
1174	Tak	Northern	2014	1.1.3.1	MDR-TB	SRR11662263
1175	Ratchaburi	Central	2017	4.5	MDR-TB	SRR11662264
1176	Nakhon Sawan	Northern	2016	2.2.1	MDR-TB	SRR11662265
1177	'rachuap Khiri Khar	Central	2017	1.2.1.2.1	MDR-TB	SRR11662266
1178	Nakhon Pathom	Central	2017	2.2.1	MDR-TB	SRR11662267
1179	'rachuap Khiri Khar	Central	2017	2.2.1.1	Pre-XDR-TB	SRR11662268
1180	Kanchanaburi	Central	2017	2.2.1	MDR-TB	SRR11662269
1181	Phetchaburi	Central	2017	2.2.1	MDR-TB	SRR11662270
1182	Kanchanaburi	Central	2017	2.2.1	Pre-XDR-TB	SRR11662271
1183	Bangkok	Central	2014	2.2.1	MDR-TB	SRR11662279
1184	Chiang Rai	Northern	2017	1.1.1	Pre-XDR-TB	SRR11662290
1185	Buri Ram	Northeastern	2014	2.1	MDR-TB	SRR11662293
1186	Trat	Central	2014	2.1	MDR-TB	SRR11662296
1187	Ranong	Southern	2015	2.2.1	Other	SRR11662298
1188	Songkhla	Southern	2015	2.2.1	MDR-TB	SRR11662299
1189	Nakhon Phanom	Northeastern	2016	1.1.1	MDR-TB	SRR11662300
1190	Yala	Southern	2014	2.2.1	MDR-TB	SRR11662301
1191	Bangkok	Central	2016	2.2.1	HR-TB	SRR11662302
1192	akhon Si Thammara	Southern	2017	4.5	MDR-TB	SRR11662303
1193	Ratchaburi	Central	2015	2.2.1	Pre-XDR-TB	SRR11662304
1194	Kanchanaburi	Central	2014	2.2.1	Pre-XDR-TB	SRR11662305

1195	Phetchaburi	Central	2014	2.2.1	MDR-TB	SRR11662306
1196	Loei	Northeastern	2016	4.4.2	MDR-TB	SRR11662307
1197	Chai Nat	Central	2016	2.1	MDR-TB	SRR11662308
1198	Bangkok	Central	2014	2.2.1	MDR-TB	SRR11662309
1199	Phitsanulok	Northern	2015	2.2.1	MDR-TB	SRR11662310
1200	Chanthaburi	Central	2014	2.2.1	MDR-TB	SRR11662311
1201	Chon Buri	Central	2016	4.2.2	MDR-TB	SRR11662312
1202	Surat Thani	Southern	2017	2.2.1	MDR-TB	SRR11662314
1203	Ubon Ratchathani	Northeastern	2014	2.2.1	Pre-XDR-TB	SRR11662315
1204	Chon Buri	Central	2016	2.2.1	MDR-TB	SRR11662316
1205	Chon Buri	Central	2016	2.2.1	Pre-XDR-TB	SRR11662317
1206	akhon Si Thammara	Southern	2016	2.2.1	MDR-TB	SRR11662318
1207	Khon Kaen	Northeastern	2016	2.2.1	Pre-XDR-TB	SRR11662319
1208	Kanchanaburi	Central	2017	2.2.1	MDR-TB	SRR11662320
1209	Bangkok	Central	2017	2.2.1	MDR-TB	SRR11662321
1210	Songkhla	Southern	2016	2.2.1	Pre-XDR-TB	SRR11662322
1211	Prachin Buri	Central	2017	2.2.1.1	Pre-XDR-TB	SRR11662323
1212	Ratchaburi	Central	2016	2.2.1	MDR-TB	SRR11662324
1213	Bangkok	Central	2017	2.1	MDR-TB	SRR11662325
1214	Bangkok	Central	2017	2.1	Pre-XDR-TB	SRR11662326
1215	Phichit	Northern	2016	2.2.1	Pre-XDR-TB	SRR11662327
1216	Chachoengsao	Central	2017	2.2.1	MDR-TB	SRR11662328
1217	Chon Buri	Central	2017	2.2.1	Pre-XDR-TB	SRR11662329
1218	Samut Prakan	Central	2017	2.2.1	MDR-TB	SRR11662330
1219	Chanthaburi	Central	2014	2.2.1	MDR-TB	SRR11662331
1220	Chachoengsao	Central	2014	2.2.1.1	Pre-XDR-TB	SRR11662332
1221	Sukhothai	Northern	2016	2.2.1	HR-TB	SRR11662333
1222	Samut Sakhon	Central	2016	2.2.1.1	HR-TB	SRR11662334
1223	Kanchanaburi	Central	2016	2.2.1	MDR-TB	SRR11662335
1224	Kanchanaburi	Central	2015	2.2.1	MDR-TB	SRR11662336
1225	Kanchanaburi	Central	2017	2.2.1	MDR-TB	SRR11662337
1226	Kanchanaburi	Central	2015	2.2.1	MDR-TB	SRR11662338
1227	Suphan Buri	Central	2017	2.2.1	MDR-TB	SRR11662339
1228	Ratchaburi	Central	2015	2.2.1	MDR-TB	SRR11662340
1229	Kanchanaburi	Central	2017	2.2.1	MDR-TB	SRR11662341
1230	Samut Sakhon	Central	2017	2.2.1	MDR-TB	SRR11662342
1231	Songkhla	Southern	2014	2.2.1	MDR-TB	SRR11662343
1232	Bangkok	Central	2017	2.2.1	Pre-XDR-TB	SRR11662344
1233	Chiang Mai	Northern	2015	1.1.1	MDR-TB	SRR11662345
1234	Chachoengsao	Central	2014	2.2.1	MDR-TB	SRR11662346
1235	Chon Buri	Central	2017	2.2.1	MDR-TB	SRR11662347
1236	Bangkok	Central	2017	2.2.1	Pre-XDR-TB	SRR11662348
1237	Rayong	Central	2014	2.2.1	MDR-TB	SRR11662349
1238	Songkhla	Southern	2016	2.2.1	MDR-TB	SRR11662350
1239	Chaiyaphum	Northeastern	2016	2.2.1	Pre-XDR-TB	SRR11662352
1240	Nakhon Ratchasima	Northeastern	2016	2.2.1	MDR-TB	SRR11662353
1241	Suphan Buri	Central	2014	2.2.1	MDR-TB	SRR11662354
1242	Phatthalung	Southern	2017	2.2.1	MDR-TB	SRR11662355
1243	Kanchanaburi	Central	2014	2.2.1	MDR-TB	SRR11662356
1244	Kanchanaburi	Central	2014	2.2.1	MDR-TB	SRR11662357
1245	Bangkok	Central	2017	2.2.1	MDR-TB	SRR11662358
1246	Bangkok	Central	2017	2.2.1	Pre-XDR-TB	SRR11662359

1247	Nonthaburi	Central	2014	4.4.2	MDR-TB	SRR11662360
1248	Chon Buri	Central	2016	2.2.1	MDR-TB	SRR11662361
1249	Rayong	Central	2016	4.2.2	Pre-XDR-TB	SRR11662362
1250	Uthai Thani	Northern	2016	2.2.1	MDR-TB	SRR11662363
1251	Chon Buri	Central	2016	2.2.1	MDR-TB	SRR11662364
1252	Chon Buri	Central	2014	2.2.1	HR-TB	SRR11662365
1253	Roi Et	Northeastern	2016	2.2.1	MDR-TB	SRR11662367
1254	Bangkok	Central	2016	2.2.1	Pre-XDR-TB	SRR11662373
1255	Uttaradit	Northern	2015	2.2.1	MDR-TB	SRR11662378
1256	Chanthaburi	Central	2015	2.2.1	MDR-TB	SRR11662379
1257	Chanthaburi	Central	2015	2.2.1	MDR-TB	SRR11662380
1258	Samut Prakan	Central	2015	2.2.1	MDR-TB	SRR11662381
1259	Chanthaburi	Central	2015	2.2.1	MDR-TB	SRR11662382
1260	Chanthaburi	Central	2015	2.2.1	MDR-TB	SRR11662383
1261	Bangkok	Central	2014	2.2.1	MDR-TB	SRR11662384
1262	Bangkok	Central	2015	2.2.1	MDR-TB	SRR11662385
1263	Bangkok	Central	2015	4.5	MDR-TB	SRR11662386
1264	Amnat Charoen	Northeastern	2015	2.2.1	MDR-TB	SRR11662387
1265	Si Sa Ket	Northeastern	2015	1.1.1	Pre-XDR-TB	SRR11662388
1266	Ubon Ratchathani	Northeastern	2015	2.2.1.1	MDR-TB	SRR11662389
1267	Nakhon Phanom	Northeastern	2015	2.2.1	MDR-TB	SRR11662390
1268	Narathiwat	Southern	2015	2.2.1	MDR-TB	SRR11662391
1269	Samut Prakan	Central	2015	2.2.1	MDR-TB	SRR11662392
1270	a Nakhon Si Ayutth	Central	2015	2.2.1	MDR-TB	SRR11662393
1271	Ubon Ratchathani	Northeastern	2016	2.2.1	Pre-XDR-TB	SRR11662394
1272	Yasothon	Northeastern	2016	1.1.1	MDR-TB	SRR11662395
1273	Bangkok	Central	2016	4.5	Pre-XDR-TB	SRR11662396
1274	Chai Nat	Central	2017	2.2.1	MDR-TB	SRR11662397
1275	Nonthaburi	Central	2017	2.2.1	MDR-TB	SRR11662398
1276	Phichit	Northern	2014	1.1.1	MDR-TB	SRR11662399
1277	akhon Si Thammar	Southern	2015	1.1.3.3	MDR-TB	SRR11662400
1278	Phuket	Southern	2015	2.2.1.1	MDR-TB	SRR11662401
1279	Phuket	Southern	2014	2.2.1.1	Pre-XDR-TB	SRR11662402
1280	Phatthalung	Southern	2015	2.2.1	MDR-TB	SRR11662403
1281	Bangkok	Central	2015	2.2.1.1	MDR-TB	SRR11662404
1282	Nonthaburi	Central	2017	2.2.1	MDR-TB	SRR11662405
1283	Samut Sakhon	Central	2015	1.1.1	MDR-TB	SRR11662406
1284	Chaiyaphum	Northeastern	2016	2.2.1	Pre-XDR-TB	SRR11662407
1285	Phichit	Northern	2017	2.2.1	Pre-XDR-TB	SRR11662408
1286	Buri Ram	Northeastern	2016	2.2.1	MDR-TB	SRR11662409
1287	Bangkok	Central	2014	2.2.2	HR-TB	SRR11662410
1288	Bangkok	Central	2016	2.2.1	MDR-TB	SRR11662411
1289	Bangkok	Central	2014	2.2.1	Pre-XDR-TB	SRR11662412
1290	Amnat Charoen	Northeastern	2015	2.2.1	Pre-XDR-TB	SRR11662413
1291	Sing Buri	Central	2016	2.2.1	MDR-TB	SRR11662414
1292	akhon Si Thammar	Southern	2016	4.2.2	MDR-TB	SRR11662415
1293	Kanchanaburi	Central	2017	3	MDR-TB	SRR11662416
1294	Bangkok	Central	2016	2.2.1	MDR-TB	SRR11662418
1295	Chiang Mai	Northern	2015	2.2.1	MDR-TB	SRR11662419
1296	Chiang Mai	Northern	2015	2.2.1	MDR-TB	SRR11662420
1297	akhon Si Thammar	Southern	2015	2.2.1	MDR-TB	SRR11662421
1298	Prachin Buri	Central	2014	2.2.1.1	MDR-TB	SRR11662422

1299	Amnat Charoen	Northeastern	2014	1.1.3	MDR-TB	SRR11662423
1300	Sakon Nakhon	Northeastern	2014	1.1.1.1	MDR-TB	SRR11662424
1301	Ubon Ratchathani	Northeastern	2014	1.1.1	MDR-TB	SRR11662425
1302	Phuket	Southern	2014	2.2.1.1	MDR-TB	SRR11662426
1303	Phitsanulok	Northern	2017	2.2.1	MDR-TB	SRR11662427
1304	Phetchabun	Northern	2017	2.2.1	MDR-TB	SRR11662428
1305	Bangkok	Central	2017	2.2.1	MDR-TB	SRR11662429
1306	Phuket	Southern	2014	2.2.1	HR-TB	SRR11662430
1307	Phuket	Southern	2014	2.2.1	Pre-XDR-TB	SRR11662431
1308	Bangkok	Central	2014	2.2.1	MDR-TB	SRR11662432
1309	Uthai Thani	Northern	2014	1.1.1.1	MDR-TB	SRR11662433
1310	Nonthaburi	Central	2014	2.2.1	Pre-XDR-TB	SRR11662434
1311	Nonthaburi	Central	2014	2.2.1	MDR-TB	SRR11662435
1312	Ubon Ratchathani	Northeastern	2014	2.2.1	MDR-TB	SRR11662436
1313	Mukdahan	Northeastern	2014	2.2.1	MDR-TB	SRR11662437
1314	Mukdahan	Northeastern	2014	2.2.1	Pre-XDR-TB	SRR11662438
1315	akhon Si Thammara	Southern	2014	2.1	MDR-TB	SRR11662439
1316	Udon Thani	Northeastern	2016	2.2.1.1	MDR-TB	SRR11662440
1317	Bangkok	Central	2017	4.2.2	MDR-TB	SRR11662441
1318	Chachoengsao	Central	2017	2.2.1	MDR-TB	SRR11662442
1319	Maha Sarakham	Northeastern	2016	2.2.1	MDR-TB	SRR11662443
1320	Kalasin	Northeastern	2016	1.1.2	MDR-TB	SRR11662444
1321	Khon Kaen	Northeastern	2016	2.2.1	Pre-XDR-TB	SRR11662445
1322	Khon Kaen	Northeastern	2016	4.5	MDR-TB	SRR11662446
1323	Maha Sarakham	Northeastern	2016	2.2.1	MDR-TB	SRR11662447
1324	Roi Et	Northeastern	2016	1.1.1	MDR-TB	SRR11662448
1325	Maha Sarakham	Northeastern	2016	2.2.1	MDR-TB	SRR11662449
1326	Phetchaburi	Central	2016	2.2.1	MDR-TB	SRR11662451
1327	Suphan Buri	Central	2016	2.2.1	Pre-XDR-TB	SRR11662452
1328	Bangkok	Central	2017	2.2.1	MDR-TB	SRR11662453
1329	Si Sa Ket	Northeastern	2017	2.2.1	MDR-TB	SRR11662454
1330	Nakhon Pathom	Central	2016	2.2.1	MDR-TB	SRR11662455
1331	Uttaradit	Northern	2016	2.2.1	MDR-TB	SRR11662456
1332	Nonthaburi	Central	2016	2.1	MDR-TB	SRR11662457
1333	Rayong	Central	2017	4.2.2	MDR-TB	SRR11662458
1334	Maha Sarakham	Northeastern	2017	2.2.1	MDR-TB	SRR11662459
1335	Nong Khai	Northeastern	2017	2.2.1	MDR-TB	SRR11662460
1336	Maha Sarakham	Northeastern	2017	2.2.1	MDR-TB	SRR11662461
1337	Khon Kaen	Northeastern	2017	1.1.1	MDR-TB	SRR11662462
1338	Bangkok	Central	2014	2.2.1	Pre-XDR-TB	SRR11662463
1339	Surat Thani	Southern	2016	2.2.1	MDR-TB	SRR11662464
1340	Khon Kaen	Northeastern	2014	4.5	MDR-TB	SRR11662465
1341	Bangkok	Central	2014	2.2.1	MDR-TB	SRR11662466
1342	Ubon Ratchathani	Northeastern	2015	2.2.1	MDR-TB	SRR11662467
1343	Chon Buri	Central	2017	2.2.1	MDR-TB	SRR11662468
1344	Kanchanaburi	Central	2015	2.2.1	MDR-TB	SRR11662469
1345	Kanchanaburi	Central	2015	2.2.1	MDR-TB	SRR11662470
1346	Samut Sakhon	Central	2015	2.2.1	MDR-TB	SRR11662471
1347	Ratchaburi	Central	2015	2.2.1	MDR-TB	SRR11662472
1348	Ratchaburi	Central	2015	1.1.1	MDR-TB	SRR11662473
1349	Nakhon Pathom	Central	2015	2.1	MDR-TB	SRR11662474
1350	Kanchanaburi	Central	2015	2.2.1	MDR-TB	SRR11662475

1351	Phetchaburi	Central	2015	2.2.1	MDR-TB	SRR11662476
1352	Suphan Buri	Central	2015	2.2.1	MDR-TB	SRR11662477
1353	Chon Buri	Central	2014	2.2.1	Pre-XDR-TB	SRR11662479
1354	Songkhla	Southern	2016	2.2.1	MDR-TB	SRR11662480
1355	Rayong	Central	2014	1.1.1.1	MDR-TB	SRR11662481
1356	Khon Kaen	Northeastern	2017	2.2.1	MDR-TB	SRR11662483
1357	Nong Bua Lamphu	Northeastern	2017	1.1.3.3	MDR-TB	SRR11662484
1358	Ubon Ratchathani	Northeastern	2016	2.2.1	Pre-XDR-TB	SRR11662485
1359	Nong Bua Lamphu	Northeastern	2017	1.1.1	MDR-TB	SRR11662486
1360	Bangkok	Central	2017	2.2.1	MDR-TB	SRR11662487
1361	Maha Sarakham	Northeastern	2017	1.1.1	MDR-TB	SRR11662488
1362	Bangkok	Central	2014	1.1.1	MDR-TB	SRR11662489
1363	Samut Prakan	Central	2014	2.2.1	MDR-TB	SRR11662490
1364	Chanthaburi	Central	2017	1.1.3.3	MDR-TB	SRR11662491
1365	Chanthaburi	Central	2017	1.1.1	MDR-TB	SRR11662492
1366	Phang Nga	Southern	2014	2.2.1	MDR-TB	SRR11662493
1367	Chon Buri	Central	2017	2.2.1	MDR-TB	SRR11662494
1368	Chon Buri	Central	2017	2.2.1	MDR-TB	SRR11662495
1369	Yasothon	Northeastern	2014	1.1.1	MDR-TB	SRR11662496
1370	Ubon Ratchathani	Northeastern	2014	2.2.1	MDR-TB	SRR11662497
1371	Kanchanaburi	Central	2016	2.1	Pre-XDR-TB	SRR11662498
1372	Nonthaburi	Central	2017	4.2.2	MDR-TB	SRR11662499
1373	Chon Buri	Central	2014	2.2.1	Pre-XDR-TB	SRR11662500
1374	Chon Buri	Central	2014	2.2.1	Pre-XDR-TB	SRR11662501
1375	Trat	Central	2015	2.1	MDR-TB	SRR11662502
1376	Samut Prakan	Central	2017	2.1	MDR-TB	SRR11662503
1377	Phang Nga	Southern	2015	2.2.1	MDR-TB	SRR11662504
1378	Nonthaburi	Central	2015	2.2.1	MDR-TB	SRR11662505
1379	Si Sa Ket	Northeastern	2015	2.2.1	MDR-TB	SRR11662506
1380	Si Sa Ket	Northeastern	2015	2.2.1	MDR-TB	SRR11662507
1381	Phetchabun	Northern	2015	2.2.1	MDR-TB	SRR11662509
1382	Nakhon Sawan	Northern	2015	2.2.1.2	RR-TB	SRR11662510
1383	Phichit	Northern	2015	2.2.1	MDR-TB	SRR11662511
1384	Surat Thani	Southern	2015	2.2.1	MDR-TB	SRR11662512
1385	Nonthaburi	Central	2015	2.2.1	MDR-TB	SRR11662513
1386	Bangkok	Central	2015	2.1	MDR-TB	SRR11662514
1387	Bangkok	Central	2017	2.2.1	MDR-TB	SRR11662515
1388	Krabi	Southern	2015	4.5	MDR-TB	SRR11662516
1389	Trat	Central	2015	1.2.1.2.1	Other	SRR11662517
1390	Bangkok	Central	2015	2.2.1	MDR-TB	SRR11662518
1391	Chachoengsao	Central	2015	2.2.1	MDR-TB	SRR11662519
1392	Bangkok	Central	2015	2.2.1	Pre-XDR-TB	SRR11662520
1393	Chiang Mai	Northern	2015	2.2.1	MDR-TB	SRR11662521
1394	Chiang Mai	Northern	2015	2.2.1	MDR-TB	SRR11662522
1395	Bangkok	Central	2015	2.2.1	MDR-TB	SRR11662523
1396	akhon Si Thammar	Southern	2015	2.2.1	MDR-TB	SRR11662524
1397	\akhon Ratchasim	Northeastern	2015	2.2.1	MDR-TB	SRR11662525
1398	Bangkok	Central	2017	4.5	MDR-TB	SRR11662526
1399	Ranong	Southern	2014	2.2.1	Pre-XDR-TB	SRR11662527
1400	Surat Thani	Southern	2014	2.1	MDR-TB	SRR11662528
1401	Chumphon	Southern	2014	2.2.2	MDR-TB	SRR11662529
1402	Rayong	Central	2014	2.1	MDR-TB	SRR11662530

1403	Roi Et	Northeastern	2014	2.2.1	MDR-TB	SRR11662531
1404	Khon Kaen	Northeastern	2014	1.1.1	MDR-TB	SRR11662532
1405	Udon Thani	Northeastern	2014	2.2.1	MDR-TB	SRR11662533
1406	Udon Thani	Northeastern	2014	2.2.1	MDR-TB	SRR11662534
1407	Chon Buri	Central	2017	2.2.1	MDR-TB	SRR11662535
1408	Chon Buri	Central	2017	2.2.1	MDR-TB	SRR11662536
1409	Phetchabun	Northern	2016	2.2.1	MDR-TB	SRR11662537
1410	Chon Buri	Central	2017	2.2.1	Pre-XDR-TB	SRR11662538
1411	Bangkok	Central	2014	2.2.1	MDR-TB	SRR11662539
1412	Krabi	Southern	2017	2.2.1	MDR-TB	SRR11662540
1413	Nakhon Sawan	Northern	2017	2.2.1.1	MDR-TB	SRR11662541
1414	Nakhon Nayok	Central	2017	2.2.1	MDR-TB	SRR11662542
1415	Saraburi	Central	2017	2.2.1.1	MDR-TB	SRR11662543
1416	Chaiyaphum	Northeastern	2017	2.2.1	MDR-TB	SRR11662544
1417	Nakhon Ratchasima	Northeastern	2017	2.2.1.1	MDR-TB	SRR11662545
1418	Nakhon Ratchasima	Northeastern	2017	2.2.1	MDR-TB	SRR11662546
1419	Phuket	Southern	2014	2.2.1	MDR-TB	SRR11662547
1420	Tak	Northern	2016	2.2.1	MDR-TB	SRR11662548
1421	Ubon Ratchathani	Northeastern	2017	2.2.1	MDR-TB	SRR11662549
1422	Roi Et	Northeastern	2017	1.1.1	MDR-TB	SRR11662550
1423	Samut Prakan	Central	2015	2.2.1	MDR-TB	SRR11662551
1424	Sakon Nakhon	Northeastern	2014	2.2.1	MDR-TB	SRR11662552
1425	Bangkok	Central	2016	2.2.1	MDR-TB	SRR11662553
1426	Bangkok	Central	2016	2.2.1	MDR-TB	SRR11662554
1427	Prachuap Khiri Khan	Central	2014	2.2.1	MDR-TB	SRR11662555
1428	Bangkok	Central	2016	2.2.1	MDR-TB	SRR11662556
1429	Krabi	Southern	2016	2.2.1	MDR-TB	SRR11662557
1430	Chumphon	Southern	2016	2.2.1	MDR-TB	SRR11662558
1431	Nakhon Ratchasima	Northeastern	2017	2.2.1	MDR-TB	SRR11662559
1432	Phang Nga	Southern	2016	2.2.1.1	MDR-TB	SRR11662560
1433	Nakhon Si Thammarat	Southern	2016	2.2.1	MDR-TB	SRR11662561
1434	Chaiyaphum	Northeastern	2017	2.2.1	Pre-XDR-TB	SRR11662562
1435	Surat Thani	Southern	2016	2.2.1	MDR-TB	SRR11662563
1436	Kanchanaburi	Central	2017	2.2.1	MDR-TB	SRR11662564
1437	Kamphaeng Phet	Northern	2014	2.2.1	MDR-TB	SRR11662565
1438	Rayong	Central	2016	2.2.1	MDR-TB	SRR11662566
1439	Surin	Northeastern	2015	2.2.1	MDR-TB	SRR11662567
1440	Bangkok	Central	2015	2.2.1	MDR-TB	SRR11662568
1441	Mukdahan	Northeastern	2015	1.1.1	MDR-TB	SRR11662569
1442	Bangkok	Central	2015	2.2.1	Pre-XDR-TB	SRR11662570
1443	Chai Nat	Central	2015	2.2.1.1	MDR-TB	SRR11662571
1444	Bangkok	Central	2015	2.1	MDR-TB	SRR11662572
1445	Sa Kaeo	Central	2015	2.2.1	MDR-TB	SRR11662573
1446	Nakhon Sawan	Northern	2015	2.2.1	MDR-TB	SRR11662574
1447	Satun	Southern	2015	2.2.1	MDR-TB	SRR11662575
1448	Bangkok	Central	2016	4.2.2	MDR-TB	SRR11662576
1449	Rayong	Central	2016	2.2.1	MDR-TB	SRR11662577
1450	Ratchaburi	Central	2016	2.2.1	MDR-TB	SRR11662578
1451	Nakhon Pathom	Central	2016	2.2.1	MDR-TB	SRR11662579
1452	Phetchaburi	Central	2016	2.2.1	MDR-TB	SRR11662580
1453	Trat	Central	2016	1.1.1	MDR-TB	SRR11662581
1454	Nakhon Si Thammarat	Southern	2016	1.1.3.3	MDR-TB	SRR11662582

1455	Chumphon	Southern	2015	2.2.1	MDR-TB	SRR11662583
1456	Maha Sarakham	Northeastern	2015	2.2.1	MDR-TB	SRR11662584
1457	Roi Et	Northeastern	2015	2.2.1	MDR-TB	SRR11662585
1458	Udon Thani	Northeastern	2015	2.2.1	MDR-TB	SRR11662586
1459	Kalasin	Northeastern	2015	2.2.1	MDR-TB	SRR11662587
1460	akhon Si Thammar	Southern	2017	2.2.1	Pre-XDR-TB	SRR11662588
1461	Udon Thani	Northeastern	2015	2.2.1	MDR-TB	SRR11662589
1462	Chon Buri	Central	2014	2.2.1	MDR-TB	SRR11662590
1463	Tak	Northern	2017	2.2.1	MDR-TB	SRR11662591
1464	Chon Buri	Central	2017	2.2.1	MDR-TB	SRR11662592
1465	Chon Buri	Central	2017	2.2.1	MDR-TB	SRR11662593
1466	Khon Kaen	Northeastern	2017	2.2.1	MDR-TB	SRR11662594
1467	Kanchanaburi	Central	2017	2.2.1	MDR-TB	SRR11662595
1468	Ratchaburi	Central	2017	2.2.1	MDR-TB	SRR11662596
1469	Bangkok	Central	2017	2.2.1	MDR-TB	SRR11662597
1470	Samut Sakhon	Central	2017	2.2.1	HR-TB	SRR11662598
1471	Kanchanaburi	Central	2017	2.2.1	MDR-TB	SRR11662599
1472	Phetchaburi	Central	2017	1.1.1	MDR-TB	SRR11662600
1473	Samut Songkhram	Central	2017	2.2.1	MDR-TB	SRR11662601
1474	Phetchaburi	Central	2017	2.2.1	MDR-TB	SRR11662602
1475	Kanchanaburi	Central	2017	2.2.1	MDR-TB	SRR11662603
1476	Songkhla	Southern	2017	1.1.1	MDR-TB	SRR11662604
1477	Si Sa Ket	Northeastern	2014	1.1.1.1	MDR-TB	SRR11662605
1478	Songkhla	Southern	2015	1.1.1	MDR-TB	SRR11662606
1479	Lop Buri	Central	2016	2.2.1	MDR-TB	SRR11662607
1480	Phetchaburi	Central	2016	2.2.1	MDR-TB	SRR11662608
1481	akhon Si Thammar	Southern	2014	2.2.1	MDR-TB	SRR11662609
1482	Satun	Southern	2017	2.2.1	MDR-TB	SRR11662610
1483	Samut Prakan	Central	2016	2.2.1	MDR-TB	SRR11662611
1484	akhon Si Thammar	Southern	2017	1.1.3.3	Pre-XDR-TB	SRR11662612
1485	Pattani	Southern	2016	2.2.1.1	MDR-TB	SRR11662613
1486	Ubon Ratchathani	Northeastern	2017	2.2.1	MDR-TB	SRR11662614
1487	Yasothon	Northeastern	2017	1.1.1	MDR-TB	SRR11662615
1488	Si Sa Ket	Northeastern	2017	2.2.1	MDR-TB	SRR11662616
1489	Phuket	Southern	2017	2.1	MDR-TB	SRR11662617
1490	Prachin Buri	Central	2017	2.2.1	MDR-TB	SRR11662618
1491	Sa Kaeo	Central	2017	1.1.1	MDR-TB	SRR11662619
1492	Nakhon Sawan	Northern	2016	2.1	MDR-TB	SRR11662620
1493	Bangkok	Central	2014	2.2.1	Pre-XDR-TB	SRR11662621
1494	Tak	Northern	2014	2.2.1.1	MDR-TB	SRR11662622
1495	Uttaradit	Northern	2016	2.2.1	MDR-TB	SRR11662623
1496	Bangkok	Central	2016	2.2.1	Pre-XDR-TB	SRR11662624
1497	Bangkok	Central	2016	2.2.1	MDR-TB	SRR11662625
1498	Kamphaeng Phet	Northern	2017	1.1.1	MDR-TB	SRR11662626
1499	Chiang Mai	Northern	2017	2.1	MDR-TB	SRR11662627
1500	Chiang Mai	Northern	2017	2.1	MDR-TB	SRR11662628
1501	Chiang Mai	Northern	2017	2.2.1	MDR-TB	SRR11662629
1502	Chiang Mai	Northern	2017	2.2.1	MDR-TB	SRR11662630
1503	Buri Ram	Northeastern	2017	2.2.1	MDR-TB	SRR11662631
1504	Buri Ram	Northeastern	2017	2.2.1.1	MDR-TB	SRR11662632
1505	Surin	Northeastern	2017	2.2.1	MDR-TB	SRR11662633
1506	Rayong	Central	2017	2.2.1	MDR-TB	SRR11662634

1507	Bangkok	Central	2016	2.2.2	Pre-XDR-TB	SRR11662635
1508	Ubon Ratchathani	Northeastern	2014	2.2.1	MDR-TB	SRR11662636
1509	Sakon Nakhon	Northeastern	2016	2.2.1	MDR-TB	SRR11662637
1510	Kanchanaburi	Central	2017	2.2.1	MDR-TB	SRR11662638
1511	Samut Sakhon	Central	2017	2.2.1	MDR-TB	SRR11662639
1512	Suphan Buri	Central	2017	2.2.1	MDR-TB	SRR11662640
1513	Kanchanaburi	Central	2017	2.2.1	MDR-TB	SRR11662641
1514	Samut Songkhram	Central	2017	2.2.1	MDR-TB	SRR11662642
1515	Tak	Northern	2014	1.1.1	HR-TB	SRR11662643
1516	Suphan Buri	Central	2017	2.2.1.1	MDR-TB	SRR11662644
1517	Kanchanaburi	Central	2017	2.2.1	MDR-TB	SRR11662645
1518	Kanchanaburi	Central	2017	2.2.1	MDR-TB	SRR11662646
1519	Phetchaburi	Central	2017	2.2.1	MDR-TB	SRR11662647
1520	Kanchanaburi	Central	2017	2.2.1	Pre-XDR-TB	SRR11662648
1521	Bangkok	Central	2016	2.2.1	MDR-TB	SRR11662649
1522	Phetchaburi	Central	2017	2.2.1	MDR-TB	SRR11662650
1523	Kanchanaburi	Central	2017	2.2.1	MDR-TB	SRR11662651
1524	Kanchanaburi	Central	2017	2.2.1	MDR-TB	SRR11662652
1525	Bangkok	Central	2017	2.2.1	MDR-TB	SRR11662653
1526	Chon Buri	Central	2014	2.2.1	MDR-TB	SRR11662654
1527	Trang	Southern	2016	2.2.1	MDR-TB	SRR11662655
1528	Pattani	Southern	2017	1.1.1	MDR-TB	SRR11662656
1529	Bangkok	Central	2017	2.2.1	MDR-TB	SRR11662657
1530	Samut Prakan	Central	2017	2.2.1	MDR-TB	SRR11662658
1531	Chon Buri	Central	2017	2.2.1	MDR-TB	SRR11662659
1532	Chon Buri	Central	2017	2.2.1	MDR-TB	SRR11662660
1533	Chon Buri	Central	2017	4.4.2	MDR-TB	SRR11662661
1534	Kanchanaburi	Central	2014	2.2.1	MDR-TB	SRR11662662
1535	Si Sa Ket	Northeastern	2016	2.2.1.2	MDR-TB	SRR11662663
1536	Kanchanaburi	Central	2014	2.2.1	MDR-TB	SRR11662664
1537	Ratchaburi	Central	2014	2.1	MDR-TB	SRR11662666
1538	Si Sa Ket	Northeastern	2016	1.1.3.1	MDR-TB	SRR11662667
1539	Si Sa Ket	Northeastern	2016	1.1.1.1	MDR-TB	SRR11662668
1540	Nonthaburi	Central	2016	2.1	MDR-TB	SRR11662669
1541	Chon Buri	Central	2016	2.2.1	Pre-XDR-TB	SRR11662670
1542	Bangkok	Central	2014	2.2.1	MDR-TB	SRR11662671
1543	Phuket	Southern	2017	2.2.1	MDR-TB	SRR11662672
1544	Chachoengsao	Central	2017	2.2.1	MDR-TB	SRR11662673
1545	Sa Kaeo	Central	2017	2.2.1	MDR-TB	SRR11662674
1546	Trat	Central	2017	2.2.1	Pre-XDR-TB	SRR11662675
1547	Chon Buri	Central	2014	2.2.1	MDR-TB	SRR11662676
1548	Trat	Central	2017	2.1	Pre-XDR-TB	SRR11662677
1549	Rayong	Central	2014	2.2.1	MDR-TB	SRR11662678
1550	Yala	Southern	2017	2.2.1	MDR-TB	SRR11662679
1551	Bangkok	Central	2017	2.2.1	MDR-TB	SRR11662680
1552	Chanthaburi	Central	2016	2.2.1	MDR-TB	SRR11662681
1553	Chon Buri	Central	2016	2.2.1	Pre-XDR-TB	SRR11662682
1554	Phetchaburi	Central	2016	2.2.1	HR-TB	SRR11662683
1555	Bangkok	Central	2016	2.2.1	MDR-TB	SRR11662684
1556	Loei	Northeastern	2017	2.2.1	Pre-XDR-TB	SRR11662685
1557	Amnat Charoen	Northeastern	2017	2.2.1	MDR-TB	SRR11662686
1558	Ubon Ratchathani	Northeastern	2017	1.1.1	MDR-TB	SRR11662687

1559	Bangkok	Central	2017	2.2.1	MDR-TB	SRR11662688
1560	Sa Kaeo	Central	2014	2.2.1	MDR-TB	SRR11662689
1561	Chanthaburi	Central	2016	2.2.1	MDR-TB	SRR11662695
1562	Khon Kaen	Northeastern	2015	2.2.1	MDR-TB	SRR11662698
1563	Chachoengsao	Central	2016	4.8	MDR-TB	SRR11662706
1564	Bueng Kan	Northeastern	2015	2.2.1	MDR-TB	SRR11662710
1565	Roi Et	Northeastern	2015	2.2.1	MDR-TB	SRR11662711
1566	Loei	Northeastern	2015	2.2.1	RR-TB	SRR11662712
1567	Ranong	Southern	2016	2.2.1	Pre-XDR-TB	SRR11662713
1568	Bangkok	Central	2016	2.2.1	MDR-TB	SRR11662714
1569	Kanchanaburi	Central	2015	2.2.1	MDR-TB	SRR11662715
1570	Kanchanaburi	Central	2015	2.2.1	MDR-TB	SRR11662716
1571	Suphan Buri	Central	2015	2.2.1	Pre-XDR-TB	SRR11662717
1572	Trat	Central	2015	2.2.1	Pre-XDR-TB	SRR11662718
1573	akhon Si Thammar	Southern	2017	2.2.1	MDR-TB	SRR11662719
1574	Phetchaburi	Central	2015	2.2.1	Pre-XDR-TB	SRR11662720
1575	Sing Buri	Central	2015	2.2.1	MDR-TB	SRR11662721
1576	Kanchanaburi	Central	2008	2.2.1	MDR-TB	SRR1595970
1577	Kanchanaburi	Central	2008	2.2.1	MDR-TB	SRR1595971
1578	Kanchanaburi	Central	2008	2.2.1	MDR-TB	SRR1595972
1579	Kanchanaburi	Central	2006	2.2.1	MDR-TB	SRR5184979
1580	Kanchanaburi	Central	2004	2.2.1	MDR-TB	SRR5184984
1581	Chiang Rai	Northern	2007	2.2.1	Sensitive	SRR5217416
1582	Chiang Rai	Northern	2008	2.2.1	Sensitive	SRR5217417
1583	Chiang Rai	Northern	2009	2.2.1	Sensitive	SRR5217418
1584	Chiang Rai	Northern	2006	2.2.1	Sensitive	SRR5217419
1585	Chiang Rai	Northern	2005	2.2.1	Sensitive	SRR5217420
1586	Chiang Rai	Northern	2005	2.2.1	Other	SRR5217422
1587	Bangkok	Central	1999	2.2.1	HR-TB	SRR5217423
1588	Bangkok	Central	1994	2.2.1	Sensitive	SRR5217424
1589	Bangkok	Central	1999	2.2.1	Sensitive	SRR5217425
1590	Tak	Northern	2000	2.2.1	Sensitive	SRR5217426
1591	Bangkok	Central	1999	2.2.1	RR-TB	SRR5217427
1592	Bangkok	Central	1999	2.2.1	Sensitive	SRR5217428
1593	Bangkok	Central	1998	2.2.1	HR-TB	SRR5217429
1594	Bangkok	Central	2000	2.2.1	Sensitive	SRR5217430
1595	Bangkok	Central	2000	2.2.1	RR-TB	SRR5217431
1596	Bangkok	Central	1999	2.2.1	RR-TB	SRR5217432
1597	Tak	Northern	2000	2.2.1	Sensitive	SRR5217433
1598	Bangkok	Central	2000	2.2.1	MDR-TB	SRR5217434
1599	Bangkok	Central	1999	2.2.1.1	MDR-TB	SRR5217436
1600	Bangkok	Central	1999	2.2.1.1	RR-TB	SRR5217437
1601	Bangkok	Central	1999	2.2.1	Sensitive	SRR5217438
1602	Bangkok	Central	1999	2.2.1.1	Pre-XDR-TB	SRR5217439
1603	Bangkok	Central	1998	2.2.1	Other	SRR5217440
1604	Bangkok	Central	1995	2.2.1	Sensitive	SRR5217441
1605	Kanchanaburi	Central	2011	2.2.1	MDR-TB	SRR5709753
1606	Suphan Buri	Central	2011	2.1	Pre-XDR-TB	SRR5709762
1607	Kanchanaburi	Central	2011	2.2.1	Pre-XDR-TB	SRR5709763
1608	Kanchanaburi	Central	2007	2.1	Pre-XDR-TB	SRR5709764
1609	Kanchanaburi	Central	2010	1.1.1	Sensitive	SRR5709767
1610	Bangkok	Central	2009	2.2.1	Pre-XDR-TB	SRR5709839

1611	Ratchaburi	Central	2012	2.1	Pre-XDR-TB	SRR5709872
1612	Phitsanulok	Northern	2008	2.2.1	Pre-XDR-TB	SRR5709886
1613	Bangkok	Central	2008	2.1	Pre-XDR-TB	SRR5709887
1614	Kanchanaburi	Central	2008	2.2.1	Pre-XDR-TB	SRR5709889
1615	Kanchanaburi	Central	2008	2.2.1	Pre-XDR-TB	SRR5709891
1616	Chachoengsao	Central	2008	2.1	Pre-XDR-TB	SRR5709899
1617	Kanchanaburi	Central	2009	2.2.1	Pre-XDR-TB	SRR5709928
1618	Kanchanaburi	Central	2007	2.2.1	Pre-XDR-TB	SRR5709932
1619	Kanchanaburi	Central	2007	2.1	Pre-XDR-TB	SRR5709934
1620	Kanchanaburi	Central	2003	4.3.4.2	Pre-XDR-TB	SRR5709938
1621	Kanchanaburi	Central	2008	2.2.1	XDR-TB	SRR5710023
1622	Kanchanaburi	Central	2012	2.2.1	Pre-XDR-TB	SRR5710026
1623	Kanchanaburi	Central	2012	2.1	Pre-XDR-TB	SRR5710027
1624	Kanchanaburi	Central	2008	2.1	Pre-XDR-TB	SRR5837708
1625	Kanchanaburi	Central	2007	4.5	MDR-TB	ERR14105131
1626	Vakhon Ratchasima	Northeastern	2012	2.2.1	MDR-TB	ERR14105197
1627	Kanchanaburi	Central	2005	2.2.1	MDR-TB	ERR14105148
1628	Nong Khai	Northeastern	2011	2.2.1	MDR-TB	ERR14105116
1629	Kanchanaburi	Central	2006	2.2.1	MDR-TB	ERR14105120
1630	Kanchanaburi	Central	2007	2.2.1	MDR-TB	ERR14105123
1631	Kanchanaburi	Central	2007	2.2.1	MDR-TB	ERR14105185
1632	Kanchanaburi	Central	2007	2.2.1	MDR-TB	ERR14105216
1633	Kanchanaburi	Central	2007	2.2.1	MDR-TB	ERR14105133
1634	Bangkok	Central	2008	2.2.1.1	MDR-TB	ERR14105119
1635	Nong Khai	Northeastern	2011	1.1.1	MDR-TB	ERR14105138
1636	Kanchanaburi	Central	2011	2.2.1	MDR-TB	ERR14105165
1637	Bangkok	Central	2012	4.5	MDR-TB	ERR14105153
1638	Kanchanaburi	Central	2009	1.1.3.1	Other	ERR14105235
1639	Kanchanaburi	Central	2010	2.2.1	HR-TB	ERR14105168
1640	Kanchanaburi	Central	2005	4.3.4.2	MDR-TB	ERR14105251
1641	Vakhon Ratchasima	Northeastern	2012	2.2.1	MDR-TB	ERR14105141
1642	Kanchanaburi	Central	2007	2.2.1	MDR-TB	ERR14105258
1643	Kanchanaburi	Central	2007	2.2.1	MDR-TB	ERR14105139
1644	Kanchanaburi	Central	2010	2.2.1	MDR-TB	ERR14105237
1645	Kanchanaburi	Central	2010	2.2.1	MDR-TB	ERR14105196
1646	Kanchanaburi	Central	2010	2.2.1	MDR-TB	ERR14105183
1647	Kanchanaburi	Central	2010	2.2.1	MDR-TB	ERR14105173
1648	Kanchanaburi	Central	2011	2.2.1	MDR-TB	ERR14105254
1649	Kanchanaburi	Central	2011	1.1.1	RR-TB	ERR14105128
1650	Kanchanaburi	Central	2011	2.2.1.1	MDR-TB	ERR14105158
1651	Kanchanaburi	Central	2006	2.2.1	MDR-TB	ERR14105255
1652	Bangkok	Central	2004	4.2.2	Pre-XDR-TB	ERR14105114
1653	Kanchanaburi	Central	2007	2.2.1	Sensitive	ERR14105199
1654	Kanchanaburi	Central	2005	1.1.2	Sensitive	ERR14105129
1655	Kanchanaburi	Central	2007	1.1.1	Sensitive	ERR14105217
1656	Rayong	Central	2009	2.2.1	Pre-XDR-TB	ERR14105231
1657	Nonthaburi	Central	2001	1.1.1	MDR-TB	ERR14105087
1658	Nakhon Pathom	Central	2002	4.4.2	Sensitive	ERR14105247
1659	Chachoengsao	Central	2002	2.2.1	MDR-TB	ERR14105191
1660	Bangkok	Central	2002	4.5	MDR-TB	ERR14105093
1661	Phichit	Northern	2002	2.2.1	RR-TB	ERR14105203
1662	Sakon Nakhon	Northeastern	2003	4.8	MDR-TB	ERR14105117

1663	Kanchanaburi	Central	2009	1.2.1.2.1	Sensitive	ERR14105171
1664	Nakhon Ratchasima	Northeastern	2004	2.2.1	MDR-TB	ERR14105122
1665	Trang	Southern	2003	1.1.1	MDR-TB	ERR14105174
1666	Kanchanaburi	Central	2006	2.2.1	MDR-TB	ERR14105234
1667	Kanchanaburi	Central	2011	1.1.1	MDR-TB	ERR14105098
1668	Kanchanaburi	Central	2007	1.1.1	Sensitive	ERR14105115
1669	Yala	Southern	2006	2.2.1	MDR-TB	ERR14105242
1670	Samut Sakhon	Central	2012	2.2.1	MDR-TB	ERR14105108
1671	Kanchanaburi	Central	2008	2.2.1	MDR-TB	ERR14105152
1672	Kanchanaburi	Central	2010	2.2.1	MDR-TB	ERR14105142
1673	Nan	Northern	2006	2.2.1	MDR-TB	ERR14105099
1674	Bangkok	Central	2006	2.2.1	MDR-TB	ERR14105095
1675	Saraburi	Central	2002	1.2.1.2.1	MDR-TB	ERR14105223
1676	Bangkok	Central	2005	2.1	MDR-TB	SRR11828963
1677	Rayong	Central	2005	2.2.1	Pre-XDR-TB	ERR14105157
1678	Chumphon	Southern	2006	2.2.1	MDR-TB	ERR14105220
1679	Chumphon	Southern	2006	4.3.4.2	MDR-TB	ERR14105134
1680	Nakhon Ratchasima	Northeastern	2006	4.5	Sensitive	ERR14105228
1681	Rayong	Central	2005	2.2.1	Pre-XDR-TB	ERR14105186
1682	Chon Buri	Central	2005	2.2.1	MDR-TB	ERR14105260
1683	Bangkok	Central	2007	4.4.2	Pre-XDR-TB	ERR14105090
1684	Rayong	Central	2007	2.2.1	MDR-TB	ERR14105202
1685	Phrae	Northern	2007	2.1	MDR-TB	SRR11828965
1686	Nakhon Ratchasima	Northeastern	2007	2.2.1	RR-TB	ERR14105225
1687	Ratchaburi	Central	2007	1.1.2	MDR-TB	ERR14105180
1688	Chiang Mai	Northern	2007	2.2.1	MDR-TB	ERR14105150
1689	Buri Ram	Northeastern	2007	2.2.1	MDR-TB	ERR14105192
1690	Bangkok	Central	2007	2.2.1	MDR-TB	ERR14105248
1691	Buri Ram	Northeastern	2007	2.2.1	MDR-TB	ERR14105194
1692	Rayong	Central	2007	2.2.1	MDR-TB	ERR14105167
1693	Rayong	Central	2008	2.2.1.1	MDR-TB	ERR14105121
1694	Bangkok	Central	2008	2.2.1	MDR-TB	ERR14105164
1695	Chon Buri	Central	2007	2.1	MDR-TB	SRR11828970
1696	Nonthaburi	Central	2007	2.2.1	MDR-TB	ERR14105226
1697	Surat Thani	Southern	2007	2.2.1	MDR-TB	ERR14105113
1698	Bangkok	Central	2008	2.2.1.1	MDR-TB	ERR14105155
1699	Nakhon Ratchasima	Northeastern	2008	2.2.1	MDR-TB	ERR14105161
1700	Nonthaburi	Central	2008	2.2.1.2	Pre-XDR-TB	ERR14105221
1701	Chanthaburi	Central	2007	4.5	MDR-TB	ERR14105189
1702	Bangkok	Central	2008	2.2.1	RR-TB	ERR14105112
1703	Yala	Southern	2008	2.2.1	MDR-TB	ERR14105181
1704	Bangkok	Central	2008	2.2.1	MDR-TB	ERR14105151
1705	Nakhon Ratchasima	Northeastern	2008	2.2.1	MDR-TB	ERR14105215
1706	Prachuap Khiri Khan	Central	2008	2.2.1	MDR-TB	ERR14105124
1707	Prachuap Khiri Khan	Central	2008	2.2.1	MDR-TB	ERR14105214
1708	Nakhon Ratchasima	Northeastern	2008	4.4.2	MDR-TB	ERR14105211
1709	Saraburi	Central	2008	2.2.1	MDR-TB	ERR14105200
1710	Roi Et	Northeastern	2008	2.2.1	MDR-TB	ERR14105208
1711	Nakhon Ratchasima	Northeastern	2008	1.1.1	MDR-TB	ERR14105163
1712	Chachoengsao	Central	2008	2.2.1	MDR-TB	ERR14105239
1713	Bangkok	Central	2008	2.2.1	HR-TB	ERR14105111
1714	Nonthaburi	Central	2008	2.2.1	MDR-TB	ERR14105207

1715	Nakhon Ratchasima	Northeastern	2008	2.2.1	MDR-TB	ERR14105253
1716	Rayong	Central	2006	2.2.1	MDR-TB	ERR14105147
1717	Nonthaburi	Central	2007	2.2.1.1	MDR-TB	ERR14105241
1718	Suphan Buri	Central	2008	2.2.1	MDR-TB	ERR14105146
1719	Nakhon Ratchasima	Northeastern	2008	2.2.1.1	MDR-TB	ERR14105170
1720	Nakhon Ratchasima	Northeastern	2008	2.2.1.1	MDR-TB	ERR14105110
1721	Surat Thani	Southern	2008	2.2.1	MDR-TB	ERR14105154
1722	Nong Khai	Northeastern	2008	2.2.1	MDR-TB	ERR14105109
1723	Nakhon Ratchasima	Northeastern	2008	2.1	MDR-TB	SRR11828969
1724	Bangkok	Central	2008	2.2.1	MDR-TB	ERR14105088
1725	Yala	Southern	2008	2.2.1	MDR-TB	ERR14105236
1726	Satun	Southern	2008	1.1.3.3	RR-TB	ERR14105130
1727	Nakhon Ratchasima	Northeastern	2008	2.2.1.1	MDR-TB	ERR14105219
1728	Kamphaeng Phet	Northern	2008	2.2.1	MDR-TB	ERR14105105
1729	Bangkok	Central	2008	2.2.1	MDR-TB	ERR14105205
1730	Yala	Southern	2008	1.1.1	MDR-TB	ERR14105201
1731	Phrae	Northern	2008	2.2.1.1	MDR-TB	ERR14105172
1732	Kamphaeng Phet	Northern	2008	2.2.1	MDR-TB	ERR14105156
1733	Surat Thani	Southern	2008	2.2.1	MDR-TB	ERR14105184
1734	Bangkok	Central	2008	2.2.1.2	Pre-XDR-TB	ERR14105104
1735	Phitsanulok	Northern	2008	4.5	MDR-TB	ERR14105187
1736	Bangkok	Central	2008	2.2.1	Pre-XDR-TB	ERR14105126
1737	Bangkok	Central	2008	2.2.1.1	MDR-TB	ERR14105193
1738	Suphan Buri	Central	2011	2.2.1	MDR-TB	ERR14105175
1739	Kanchanaburi	Central	2009	2.2.1	MDR-TB	ERR14105102
1740	Kanchanaburi	Central	2004	1.2.1.2.1	Sensitive	ERR14105210
1741	Chon Buri	Central	2006	2.2.1.1	MDR-TB	ERR14105096
1742	Bangkok	Central	2008	1.2.1.2.1	MDR-TB	ERR14105252
1743	Satun	Southern	2008	2.2.1	MDR-TB	ERR14105259
1744	Phitsanulok	Northern	2007	2.2.1	Pre-XDR-TB	ERR14105224
1745	Bangkok	Central	2009	2.2.1.1	MDR-TB	ERR14105097
1746	Suphan Buri	Central	2009	2.2.1	MDR-TB	ERR14105249
1747	Suphan Buri	Central	2009	2.2.1	RR-TB	ERR14105159
1748	Phitsanulok	Northern	2009	4.4.2	MDR-TB	ERR14105118
1749	Nakhon Ratchasima	Northeastern	2009	2.2.1	Other	ERR14105160
1750	Surat Thani	Southern	2009	4.2.1.1	Pre-XDR-TB	ERR14105092
1751	Phatthalung	Southern	2009	2.2.1	Pre-XDR-TB	ERR14105132
1752	Rayong	Central	2009	2.2.1	MDR-TB	ERR14105195
1753	Bangkok	Central	2009	2.2.1	MDR-TB	ERR14105166
1754	Chachoengsao	Central	2009	1.1.1	MDR-TB	ERR14105245
1755	Ratchaburi	Central	2009	2.2.1	MDR-TB	ERR14105218
1756	Nakhon Ratchasima	Northeastern	2009	2.2.1	Pre-XDR-TB	ERR14105229
1757	Rayong	Central	2009	2.1	MDR-TB	SRR11828964
1758	Bangkok	Central	2009	2.2.1	MDR-TB	ERR14105101
1759	Buri Ram	Northeastern	2009	2.2.1	Pre-XDR-TB	ERR14105257
1760	Nong Khai	Northeastern	2009	2.2.1.2	MDR-TB	ERR14105190
1761	Suphan Buri	Central	2009	2.2.1	MDR-TB	ERR14105143
1762	Buri Ram	Northeastern	2009	4.5	MDR-TB	ERR14105144
1763	Bangkok	Central	2009	2.2.1	MDR-TB	ERR14105212
1764	Sukhothai	Northern	2009	2.2.1	MDR-TB	ERR14105250
1765	Chachoengsao	Central	2009	2.2.1	MDR-TB	ERR14105136
1766	Chachoengsao	Central	2009	2.2.1	MDR-TB	ERR14105182

1767	Phatthalung	Southern	2009	2.2.1	MDR-TB	ERR14105107
1768	Kanchanaburi	Central	2010	1.1.3	Sensitive	ERR14105178
1769	Phitsanulok	Northern	2009	1.2.1.2	MDR-TB	ERR14105209
1770	Nakhon Ratchasima	Northeastern	2009	2.2.1	MDR-TB	ERR14105140
1771	Rayong	Central	2009	2.2.1	MDR-TB	ERR14105169
1772	Bangkok	Central	2009	2.1	MDR-TB	SRR11828962
1773	Surat Thani	Southern	2009	2.2.1	MDR-TB	ERR14105177
1774	Nakhon Ratchasima	Northeastern	2009	2.1	MDR-TB	SRR11828968
1775	Nonthaburi	Central	2009	2.2.1.1	MDR-TB	ERR14105103
1776	Satun	Southern	2009	2.2.1	MDR-TB	ERR14105127
1777	Bangkok	Central	2005	1.1.1	MDR-TB	ERR14105227
1778	Prachuap Khiri Khan	Central	2009	2.2.1.2	RR-TB	ERR14105162
1779	Rayong	Central	2009	2.2.1	MDR-TB	ERR14105230
1780	Bangkok	Central	2009	2.2.1	MDR-TB	ERR14105213
1781	Buri Ram	Northeastern	2009	2.2.1.1	MDR-TB	ERR14105240
1782	Satun	Southern	2009	2.2.1	MDR-TB	ERR14105091
1783	Nong Khai	Northeastern	2010	2.2.1	MDR-TB	ERR14105246
1784	Suphan Buri	Central	2010	2.2.1	Pre-XDR-TB	ERR14105106
1785	Kanchanaburi	Central	2011	1.1.1.1	RR-TB	ERR14105206
1786	Yala	Southern	2005	1.1.3.3	MDR-TB	ERR14105176
1787	Nakhon Ratchasima	Northeastern	2010	2.2.1	MDR-TB	ERR14105094
1788	Nakhon Ratchasima	Northeastern	2010	4.4.2	HR-TB	ERR14105125
1789	Saraburi	Central	2010	2.1	MDR-TB	SRR11828961
1790	Suphan Buri	Central	2010	1.1.3.3	MDR-TB	ERR14105244
1791	Bangkok	Central	2010	2.2.1	MDR-TB	ERR14105262
1792	Bangkok	Central	2011	2.2.1	Pre-XDR-TB	ERR14105100
1793	Ratchaburi	Central	2011	2.2.1	MDR-TB	ERR14105232
1794	Bangkok	Central	2011	2.2.1.2	MDR-TB	ERR14105149
1795	Nakhon Ratchasima	Northeastern	2011	2.1	MDR-TB	SRR11828967
1796	Buri Ram	Northeastern	2011	2.1	MDR-TB	SRR11828971
1797	Roi Et	Northeastern	2011	1.1.3.3	MDR-TB	ERR14105204
1798	Nakhon Ratchasima	Northeastern	2011	2.2.1	MDR-TB	ERR14105243
1799	Nakhon Ratchasima	Northeastern	2011	2.2.1	MDR-TB	ERR14105137
1800	Buri Ram	Northeastern	2011	2.2.1	MDR-TB	ERR14105261
1801	Satun	Southern	2011	2.2.2	MDR-TB	ERR14105198
1802	Saraburi	Central	2011	2.2.1	MDR-TB	ERR14105145
1803	Buri Ram	Northeastern	2011	2.2.1	MDR-TB	ERR14105179
1804	Phetchabun	Northern	2010	2.2.1	MDR-TB	ERR14105256
1805	Rayong	Central	2010	2.2.1	MDR-TB	ERR14105238
1806	Surat Thani	Southern	2010	2.1	MDR-TB	SRR11828960
1807	Nonthaburi	Central	2010	1.1.1	Sensitive	ERR14105233
1808	Suphan Buri	Central	2010	2.2.1	MDR-TB	ERR14105222
1809	Saraburi	Central	2010	2.2.1	MDR-TB	ERR14105188
1810	Nakhon Ratchasima	Northeastern	2010	1.1.1	MDR-TB	ERR14105089
1811	Nakhon Ratchasima	Northeastern	2010	2.2.1	MDR-TB	ERR14105135
1812	Rayong	Central	2004	2.2.1	Pre-XDR-TB	SRR5709933
1813	Chanthaburi	Central	2007	2.2.1	Pre-XDR-TB	SRR5709935
1814	Chachoengsao	Central	2007	2.2.1	Pre-XDR-TB	SRR5709902
1815	Phrae	Northern	2008	2.2.1	Pre-XDR-TB	SRR5709892
1816	Ratchaburi	Central	2008	2.2.1	Pre-XDR-TB	SRR5709890
1817	Chachoengsao	Central	2008	2.2.1	Pre-XDR-TB	SRR5709888
1818	Songkhla	Southern	2008	2.2.1	Pre-XDR-TB	SRR5709885

1819	Nakhon Ratchasima	Northeastern	2009	2.2.1	Pre-XDR-TB	SRR5709900
1820	Ratchaburi	Central	2011	2.2.1	Sensitive	SRR5709766
1821	Bangkok	Central	2011	2.2.1	Pre-XDR-TB	SRR5709765
1822	Suphan Buri	Central	2011	2.2.1	Pre-XDR-TB	SRR5709884
1823	Prachuap Khiri Khan	Central	2011	2.2.2	Pre-XDR-TB	SRR5709960
1824	Nonthaburi	Central	2012	2.2.1	Pre-XDR-TB	SRR5710028
1825	Yala	Southern	2004	2.2.1	Pre-XDR-TB	SRR5709919
1826	Ratchaburi	Central	2005	2.2.1	Pre-XDR-TB	SRR5709918
1827	Nakhon Ratchasima	Northeastern	2006	1.1.1	Pre-XDR-TB	SRR5709917
1828	Nakhon Ratchasima	Northeastern	2007	1.1.1	MDR-TB	SRR5709916
1829	Yala	Southern	2004	2.2.1	Pre-XDR-TB	SRR5709949
1830	Rayong	Central	2005	2.2.1	MDR-TB	SRR5709948
1831	Nakhon Ratchasima	Northeastern	2006	1.1.1	Pre-XDR-TB	SRR5709913
1832	Phitsanulok	Northern	2007	2.2.1	Pre-XDR-TB	SRR5709912
1833	Chon Buri	Central	2004	2.2.1	Pre-XDR-TB	SRR5837709
1834	Nonthaburi	Central	2005	2.2.1.2	Pre-XDR-TB	SRR5710030
1835	Kanchanaburi	Central	2006	4.4.2	Pre-XDR-TB	SRR5710013
1836	Bangkok	Central	2005	2.2.1	Pre-XDR-TB	SRR5709796
1837	Chumphon	Southern	2006	2.2.1	Pre-XDR-TB	SRR5709799
1838	Kanchanaburi	Central	2007	2.1	Pre-XDR-TB	SRR5709798
1839	Roi Et	Northeastern	2004	2.2.1	Pre-XDR-TB	SRR5709793
1840	Nakhon Ratchasima	Northeastern	2005	1.1	Pre-XDR-TB	SRR5709792
1841	Ratchaburi	Central	2007	2.2.1	Pre-XDR-TB	SRR5709795
1842	Nonthaburi	Central	2008	2.2.1	Pre-XDR-TB	SRR5709794
1843	Yala	Southern	2005	2.2.1	Pre-XDR-TB	SRR5709802
1844	Kanchanaburi	Central	2006	2.2.1	Pre-XDR-TB	SRR5709801
1845	Kanchanaburi	Central	2007	2.2.1	Pre-XDR-TB	SRR5709754
1846	Nakhon Ratchasima	Northeastern	2008	1.1.1	XDR-TB	SRR5709755
1847	Surat Thani	Southern	2005	2.2.1	Pre-XDR-TB	SRR5709756
1848	Rayong	Central	2006	2.2.1	Pre-XDR-TB	SRR5709757
1849	Yala	Southern	2007	1.1.1	Pre-XDR-TB	SRR5709758
1850	Nakhon Ratchasima	Northeastern	2008	2.2.2	Pre-XDR-TB	SRR5709759
1851	Prachin Buri	Central	2005	2.2.2	Pre-XDR-TB	SRR5709760
1852	Nonthaburi	Central	2006	2.2.1	Pre-XDR-TB	SRR5709761
1853	Buri Ram	Northeastern	2007	2.2.1	Pre-XDR-TB	SRR5709751
1854	Surat Thani	Southern	2008	4.2.1.1	Pre-XDR-TB	SRR5709752
1855	Bangkok	Central	2008	2.1	Pre-XDR-TB	SRR5710022
1856	Buri Ram	Northeastern	2008	2.1	Pre-XDR-TB	SRR5710021
1857	Bangkok	Central	2008	2.2.1	Pre-XDR-TB	SRR5710020
1858	Rayong	Central	2008	4.8	Pre-XDR-TB	SRR5710019
1859	Phatthalung	Southern	2009	1.1.1	Pre-XDR-TB	SRR5710018
1860	Surat Thani	Southern	2009	4.2.1.1	Pre-XDR-TB	SRR5710017
1861	Chachoengsao	Central	2009	2.2.1.1	Pre-XDR-TB	SRR5710016
1862	Bangkok	Central	2009	2.2.2	Pre-XDR-TB	SRR5710015
1863	Nakhon Ratchasima	Northeastern	2009	2.2.1	Pre-XDR-TB	SRR5710014
1864	Nakhon Ratchasima	Northeastern	2009	2.2.1	Pre-XDR-TB	SRR5709815
1865	Bangkok	Central	2009	2.2.2	Pre-XDR-TB	SRR5709988
1866	Bangkok	Central	2009	2.2.2	Pre-XDR-TB	SRR5709780
1867	Nakhon Ratchasima	Northeastern	2009	2.2.1	Pre-XDR-TB	SRR5709996
1868	Nakhon Ratchasima	Northeastern	2009	2.2.1	Pre-XDR-TB	SRR5709993
1869	Nonthaburi	Central	2009	2.2.1	MDR-TB	SRR5709989
1870	Bangkok	Central	2009	2.2.2	Pre-XDR-TB	SRR5709991

1871	Chachoengsao	Central	2010	2.2.1	Pre-XDR-TB	SRR5709870
1872	Nakhon Ratchasima	Northeastern	2010	2.2.1.1	Pre-XDR-TB	SRR5837710
1873	Nakhon Ratchasima	Northeastern	2010	2.2.1	Pre-XDR-TB	SRR5709990
1874	Suphan Buri	Central	2010	2.2.1.1	Other	SRR5709749
1875	Nakhon Ratchasima	Northeastern	2010	2.1	Pre-XDR-TB	SRR5709748
1876	Ratchaburi	Central	2011	2.2.1	MDR-TB	SRR5709871
1877	Nonthaburi	Central	2011	2.2.2	MDR-TB	SRR5837711
1878	Nakhon Ratchasima	Northeastern	2011	2.2.2	Pre-XDR-TB	SRR5709803
1879	Bangkok	Central	2011	2.2.1	Pre-XDR-TB	SRR5709903
1880	Prachuap Khiri Khan	Central	2011	2.2.1	RR-TB	SRR5709750
1881	Buri Ram	Northeastern	2012	2.1	Pre-XDR-TB	SRR5709883
1882	Nakhon Ratchasima	Northeastern	2012	2.2.1	Pre-XDR-TB	SRR5710011
1883	Bangkok	Central	2012	2.2.1.2	Pre-XDR-TB	SRR5710012
1884	Buri Ram	Northeastern	2012	2.1	Pre-XDR-TB	SRR5709897
1885	Buri Ram	Northeastern	2012	2.1	Pre-XDR-TB	SRR5709898
1886	Buri Ram	Northeastern	2012	1.1.1	Sensitive	SRR5710008
1887	Nong Khai	Northeastern	2012	2.2.1	Pre-XDR-TB	SRR5710024
1888	Phuket	Southern	2012	2.2.1	MDR-TB	SRR5709893
1889	Buri Ram	Northeastern	2012	2.2.1	Pre-XDR-TB	SRR5709894
1890	Nong Khai	Northeastern	2012	2.2.1	Pre-XDR-TB	SRR5709895
1891	Bangkok	Central	2012	1.1.1	Pre-XDR-TB	SRR5709896
1892	Nakhon Ratchasima	Northeastern	2012	2.2.1	Pre-XDR-TB	SRR5709816
1893	Bangkok	Central	2012	2.2.1	Pre-XDR-TB	SRR5709863
1894	Nakhon Ratchasima	Northeastern	2012	2.2.1	Pre-XDR-TB	SRR5709862
1895	Nakhon Ratchasima	Northeastern	2013	2.2.1	Pre-XDR-TB	SRR5709860
1896	Kanchanaburi	Central	2006	2.2.1	Pre-XDR-TB	SRR5709867
1897	Kanchanaburi	Central	2006	4.4.2	Pre-XDR-TB	SRR5709866
1898	Bangkok	Central	2006	2.2.1	Pre-XDR-TB	SRR5709865
1899	Rayong	Central	2006	2.2.1	Pre-XDR-TB	SRR5709864
1900	Phrae	Northern	2007	2.2.1	Pre-XDR-TB	SRR5709869
1901	Bangkok	Central	2007	1.1.1	Sensitive	SRR5709868
1902	Buri Ram	Northeastern	2007	2.2.1	Pre-XDR-TB	SRR5709825
1903	Suphan Buri	Central	2010	2.2.1	Pre-XDR-TB	SRR5709826
1904	Kanchanaburi	Central	2006	2.2.1	Pre-XDR-TB	SRR5709823
1905	Phetchabun	Northern	2007	2.1	MDR-TB	SRR5709824
1906	Phitsanulok	Northern	2009	4.4.2	MDR-TB	SRR5709821
1907	Buri Ram	Northeastern	2011	2.1	MDR-TB	SRR5709822
1908	Suphan Buri	Central	2006	2.2.1	MDR-TB	SRR5709817
1909	Kanchanaburi	Central	2006	2.2.1	MDR-TB	SRR5710007
1910	Phitsanulok	Northern	2007	2.2.1	MDR-TB	SRR5709901
1911	Buri Ram	Northeastern	2007	2.2.1	Pre-XDR-TB	SRR5710010
1912	Chachoengsao	Central	2007	2.2.1	MDR-TB	SRR5710009
1913	Bangkok	Central	2007	2.2.1	MDR-TB	SRR5709992
1914	Nakhon Ratchasima	Northeastern	2007	2.2.1	MDR-TB	SRR5837712
1915	Phrae	Northern	2007	1.1.3	MDR-TB	SRR5709782
1916	Bangkok	Central	2007	2.2.1	MDR-TB	SRR5710025
1917	Rayong	Central	2007	4.2.2	MDR-TB	SRR5709781
1918	Bangkok	Central	2007	2.2.1	MDR-TB	SRR5709837
1919	Phrae	Northern	2007	1.2.2.2	HR-TB	SRR5709924
1920	Surat Thani	Southern	2007	2.1	Sensitive	SRR5709925
1921	Yala	Southern	2008	2.2.1	MDR-TB	SRR5709927
1922	Suphan Buri	Central	2008	1.1.3.3	MDR-TB	SRR5709920

1923	Rayong	Central	2008	2.2.1	MDR-TB	SRR5709921
1924	Bangkok	Central	2008	2.2.1.1	MDR-TB	SRR5709922
1925	Bangkok	Central	2008	2.2.1	MDR-TB	SRR5709929
1926	Phetchabun	Northern	2008	2.2.1	MDR-TB	SRR5709964
1927	Roi Et	Northeastern	2008	2.2.1	MDR-TB	SRR5709963
1928	Rayong	Central	2008	2.2.1	MDR-TB	SRR5709962
1929	Rayong	Central	2009	2.2.1	MDR-TB	SRR5709961
1930	Phatthalung	Southern	2009	1.1.3.3	MDR-TB	SRR5709968
1931	Saraburi	Central	2009	2.2.1	MDR-TB	SRR5709966
1932	Kanchanaburi	Central	2009	2.2.1	MDR-TB	SRR5837706
1933	Kanchanaburi	Central	2009	2.2.1	MDR-TB	SRR5709965
1934	Saraburi	Central	2009	2.2.1	MDR-TB	SRR5709776
1935	Kanchanaburi	Central	2009	1.1.1	Sensitive	SRR5709777
1936	Narathiwat	Southern	2009	2.2.1	MDR-TB	SRR5709774
1937	Suphan Buri	Central	2009	2.2.1	MDR-TB	SRR5709775
1938	Bangkok	Central	2009	2.2.1	MDR-TB	SRR5709772
1939	Bangkok	Central	2009	2.2.1	MDR-TB	SRR5709773
1940	Phrae	Northern	2009	2.2.1.1	MDR-TB	SRR5709770
1941	Bangkok	Central	2009	2.2.1	Sensitive	SRR5709771
1942	Bangkok	Central	2009	2.2.1	MDR-TB	SRR5709768
1943	Kanchanaburi	Central	2009	2.2.1	MDR-TB	SRR5709769
1944	Kanchanaburi	Central	2009	2.2.1	MDR-TB	SRR5709812
1945	Kanchanaburi	Central	2009	2.2.1	MDR-TB	SRR5709811
1946	Nakhon Ratchasima	Northeastern	2009	2.1	MDR-TB	SRR5709814
1947	Phatthalung	Southern	2009	2.2.1	MDR-TB	SRR5709813
1948	Phrae	Northern	2009	2.2.1	MDR-TB	SRR5709808
1949	Kanchanaburi	Central	2009	2.2.1	MDR-TB	SRR5709807
1950	Buri Ram	Northeastern	2010	1.2.1.2.1	MDR-TB	SRR5709810
1951	Kanchanaburi	Central	2010	2.2.1	MDR-TB	SRR5709809
1952	Nakhon Ratchasima	Northeastern	2010	2.2.1	MDR-TB	SRR5709806
1953	Sukhothai	Northern	2010	2.2.1	MDR-TB	SRR5709805
1954	Bangkok	Central	2010	2.2.1	MDR-TB	SRR5709842
1955	Saraburi	Central	2010	2.2.1	Pre-XDR-TB	SRR5709843
1956	Nakhon Ratchasima	Northeastern	2010	2.2.1	MDR-TB	SRR5709844
1957	Kanchanaburi	Central	2010	2.2.1	MDR-TB	SRR5837707
1958	Buri Ram	Northeastern	2010	4.4.2	Sensitive	SRR5709845
1959	Nonthaburi	Central	2010	4.5	Sensitive	SRR5709846
1960	Suphan Buri	Central	2010	2.2.1	Pre-XDR-TB	SRR5709847
1961	Bangkok	Central	2010	1.2.1.2	Sensitive	SRR5709848
1962	Buri Ram	Northeastern	2010	1.2.1.2.1	MDR-TB	SRR5709849
1963	Bangkok	Central	2010	2.2.1	MDR-TB	SRR5709840
1964	Nakhon Ratchasima	Northeastern	2010	4.2.2	MDR-TB	SRR5709841
1965	Satun	Southern	2010	2.2.1	MDR-TB	SRR5709880
1966	Kanchanaburi	Central	2010	2.2.1	MDR-TB	SRR5709879
1967	Bangkok	Central	2010	2.2.1	MDR-TB	SRR5709878
1968	Ratchaburi	Central	2010	2.2.1	MDR-TB	SRR5709877
1969	Chachoengsao	Central	2010	2.2.1	MDR-TB	SRR5709876
1970	Satun	Southern	2010	2.2.1	MDR-TB	SRR5709875
1971	Suphan Buri	Central	2010	2.2.1	MDR-TB	SRR5709874
1972	Songkhla	Southern	2010	2.2.1	MDR-TB	SRR5709873
1973	Satun	Southern	2012	2.2.1	MDR-TB	SRR5709882
1974	Nakhon Ratchasima	Northeastern	2012	2.2.1	MDR-TB	SRR5709881

1975	Phrae	Northern	2012	2.2.2	MDR-TB	SRR5709906
1976	Roi Et	Northeastern	2012	2.2.1	MDR-TB	SRR5709907
1977	Buri Ram	Northeastern	2012	4.2.2	MDR-TB	SRR5709904
1978	Bangkok	Central	2012	2.2.1	MDR-TB	SRR5709905
1979	Buri Ram	Northeastern	2012	2.2.1	MDR-TB	SRR5709910
1980	Satun	Southern	2012	2.2.1.1	Pre-XDR-TB	SRR5709911
1981	Buri Ram	Northeastern	2012	2.2.1	MDR-TB	SRR5709908
1982	\akhon Ratchasima	Northeastern	2012	2.2.1	Pre-XDR-TB	SRR5709909
1983	Roi Et	Northeastern	2012	4.9	Sensitive	SRR5709914
1984	Phuket	Southern	2013	4.4.2	MDR-TB	SRR5709915
1985	Kanchanaburi	Central	2006	2.2.1	MDR-TB	SRR5709951
1986	Songkhla	Southern	2007	2.2.1	MDR-TB	SRR5709950
1987	Kamphaeng Phet	Northern	2008	2.2.1	MDR-TB	SRR5709953
1988	Kanchanaburi	Central	2008	2.2.1	MDR-TB	SRR5709952
1989	\akhon Ratchasima	Northeastern	2007	2.2.1.1	Sensitive	SRR5709954
1990	Roi Et	Northeastern	2007	1.1.1	Sensitive	SRR5709957
1991	Bangkok	Central	2007	2.2.1.1	Sensitive	SRR5709956
1992	Bangkok	Central	2007	2.2.1.1	Sensitive	SRR5709959
1993	Bangkok	Central	2007	2.2.1	Sensitive	SRR5709958
1994	\akhon Ratchasima	Northeastern	2007	1.1.1	Sensitive	SRR5709944
1995	Bangkok	Central	2007	1.2.1.2.1	Sensitive	SRR5709945
1996	Bangkok	Central	2007	1.1.1	Sensitive	SRR5709946
1997	Bangkok	Central	2008	4.5	Sensitive	SRR5709947
1998	Surat Thani	Southern	2009	2.2.1	Sensitive	SRR5709940
1999	Bangkok	Central	2009	1.1.1	Other	SRR5709941
2000	Bangkok	Central	2009	1.1.1.1	Sensitive	SRR5709942
2001	Bangkok	Central	2009	2.2.1	Sensitive	SRR5709943
2002	Suphan Buri	Central	2009	2.2.1	Sensitive	SRR5709936
2003	\akhon Ratchasima	Northeastern	2009	1.1.1	Pre-XDR-TB	SRR5709937
2004	Nonthaburi	Central	2009	1.1.1	Sensitive	SRR5709982
2005	Ratchaburi	Central	2009	4.4.2	Sensitive	SRR5709981
